# Euchrestifolines A–O, fifteen novel carbazole alkaloids with potent anti-ferroptotic activity from *Murraya euchrestifolia*

**DOI:** 10.1007/s13659-024-00483-7

**Published:** 2025-01-02

**Authors:** Yue-Mei Chen, Nan-Kai Cao, Si-Si Zhu, Meng Ding, Hai-Zhen Liang, Ming-Bo Zhao, Ke-Wu Zeng, Peng-Fei Tu, Yong Jiang

**Affiliations:** State Key Laboratory of Natural and Biomimetic Drugs, School of Pharmaceutical Sciences, Peking University, Beijing, 100191 People’s Republic of China

**Keywords:** *Murraya euchrestifolia*, Carbazole, Benzopyranocarbazole, Anti-ferroptosis, NO inhibition, Cytotoxicity

## Abstract

Fifteen novel carbazole alkaloids, euchrestifolines A–O (**1**–**15**), were obtained from *Murraya euchrestifolia*. Their structures were elucidated by spectroscopic analysis, Mosher’s ester, calculated ECD, and transition metal complex ECD methods. Notably, euchrestifolines A–C (**1**–**3**) are the first naturally occurring pyrrolidone carbazoles to be identified, while euchrestifolines D–F (**4**–**6**) represent rare carbazole alkaloids containing a phenylpropanyl moiety; euchrestifoline G (**7**) features a unique benzopyranocarbazole skeleton. More importantly, these compounds exhibited significant anti-ferroptotic activity, along with inhibitory effects of nitric oxide (NO) production and notable cytotoxicity. This study marks the first disclosure of carbazole's inhibitory effects against ferroptosis, and the EC_50_ values of some carbazoles ranging from 0.04 to 1 μM, substantially lower than the positive control, ferrostatin-1. In sum, this research not only enhances our understanding of carbazole alkaloids but also opens new avenues for the discovery of ferroptosis-related leading compounds.

**Graphical abstract:**

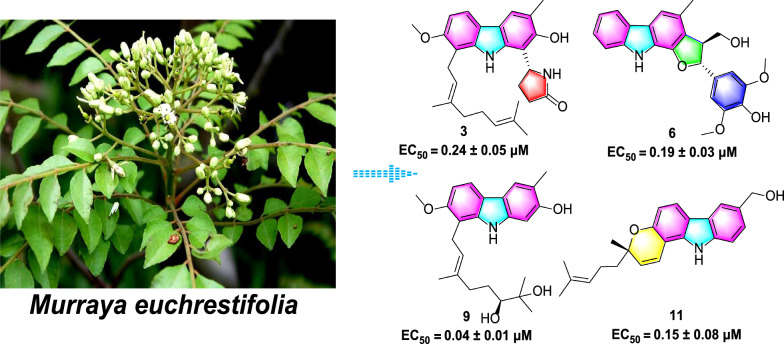

**Supplementary Information:**

The online version contains supplementary material available at 10.1007/s13659-024-00483-7.

## Introduction

*Murraya euchrestifolia* Hayata, an evergreen tree, is widely distributed in Chinese Guangdong, Guangxi, and Hainan provinces [1]. Their leaves and twigs have been widely used by local people for the treatment of inflammation and pain [2]. Previous phytochemical investigations indicated that there were abundant carbazole alkaloids [3–6] and essential oil [2] in *M. euchrestifolia*. It has been reported that the carbazole alkaloids in *Murraya* plants possess unique structures and demonstrate effective anticancer, anti-diabetic, pain-relieving, anti-inflammatory, and antimicrobial properties [7–10].

**Fig. 1:**
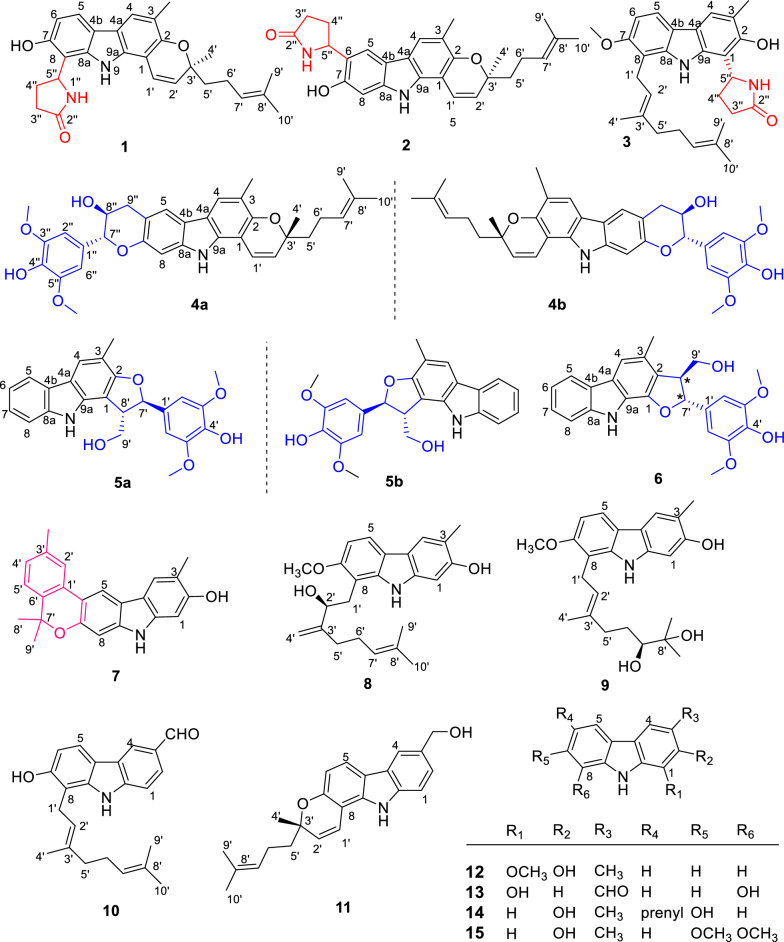
Structures of compounds **1**–**15**

In order to discover more structurally novel and biologically active carbazole alkaloids from *Murraya* species [8, 11–13], an investigation was conducted on the ethanolic extract of the leaves and twigs of *M. euchrestifolia* to yield 15 novel carbazole alkaloids, designated as euchrestifolines A–O (**1**–**15**) (Fig. 1). Among them, euchrestifolines A–C (**1**–**3**) are pyrrolidone carbazoles obtained firstly from nature; euchrestifolines D–F (**4**–**6**) are carbazoles containing a rare phenylpropanyl, and euchrestifoline G (**7**) possesses a novel benzopyranocarbazole skeleton. Compounds **4**–**6** are three racemates, resolved by chiral-phase HPLC to afford their enantiomers. In this study, we reported the isolation process and structural illustration of 15 new carbazole alkaloids, and evaluated their potential effects on anti-ferroptosis in PC12 cells, their inhibition on nitric oxide (NO) production in RAW 264.7 macrophage cells, and their cytotoxicity against HepG2 cells.

## Results and discussion

### Structural explanation

Euchrestifoline A (**1**) was isolated in the form of a brown, non-crystalline solid, [*α*]_D_^25^ + 20 (*c* 0.06, MeOH). The molecular formula C_27_H_30_N_2_O_3_ was established based on HRESIMS data (*m*/*z* 429.2169 [M − H]^–^, calcd for C_27_H_29_N_2_O_3_, 429.2178) and supported by ^13^C NMR findings. The UV spectrum showed peak absorptions at 221, 241, 296, and 312 nm, indicating the presence of a typical pyranocarbazole structure [14, 15]. The IR spectrum revealed absorption bands corresponding to hydroxy (3365 cm^−1^), carbonyl (1713 cm^−1^), olefinic, and aromatic (1648, 1612, 1517, and 1453 cm^−1^) functionalities.

The ^1^H NMR data (Table 1) presented a pair of *ortho*-coupled aromatic doublets [*δ*_H_ 6.76 (1H, d, *J* = 8.3 Hz, H-6), 7.66 (1H, d, *J* = 8.3 Hz, H-5)]. An aromatic singlet was observed at *δ*_H_ 7.57 (H-4), and a methyl group resonance appeared as a singlet at *δ*_H_ 2.28 (3-CH_3_). Additionally, signals related to 2,2-dimethyl-2*H*-pyran moiety were observed at *δ*_H_ 6.95 (1H, d, *J* = 9.8 Hz, H-1ʹ), 5.72 (1H, d, *J* = 9.8 Hz, H-2′), 1.42 (3H, s, H-4′), and 1.75 (2H, m, H-5ʹ), along with a set of prenyl signals at *δ*_H_ 2.18 (2H, m, H-6ʹ), 5.12 (1H, t, *J* = 7.4 Hz, H-7ʹ), 1.56 (3H, s, H-9ʹ), and 1.63 (3H, s, H-10ʹ).

**Table 1:** ^1^H (500 MHz) and ^13^C (125 MHz) NMR data of **1**–**6** (*δ*_H_ in ppm, *J* in Hz)

No	**1**^*a*^		**2**^*a*^		**3**^*a*^		**4**^*b*^		**5**^*a*^		**6**^*a*^	
	*δ*_H_ (*J* in Hz)	*δ*_C_, type	*δ*_H_ (*J* in Hz)	*δ*_C_, type	*δ*_H_ (*J* in Hz)	*δ*_C_, type	*δ*_H_ (*J* in Hz)	*δ*_C_, type	*δ*_H_ (*J* in Hz)	*δ*_C_, type	*δ*_H_ (*J* in Hz)	*δ*_C_, type
1		105.5, C		105.3, C		111.5, C		104.4, C		109.4, C		145.1, C
2		149.6, C		149.4, C		151.6, C		149.4, C		158.7, C		122.4, C
3		118.0, C		117.5, C		118.2, C		118.3, C		112.5, C		126.4, C
4	7.57, s	120.7, CH	7.60, s	121.0, CH	7.65, s	120.6, CH	7.54, s	120.7, CH	7.75, s	121.4, CH	7.44, s	113.7, CH
4a		117.9, C		117.8, C		118.74, C		116.5, C		118.7, C		127.1, C
4b		118.5, C		117.2, C		118.68, C		119.0, C		124.8, C		124.2, C
5	7.66, d (8.3)	119.4, CH	7.77, s	116.8, CH	7.73, d (8.5)	117.8, CH	7.60, s	119.9, CH	7.96, d (7.7)	119.7, CH	8.03, d (7.8)	120.8, CH
6	6.76, d (8.3)	109.7, CH		123.3, C	6.86, d (8.5)	105.4, CH		112.7, C	7.10, t (7.7)	119.6, CH	7.14, t (7.8)	119.6, CH
7		153.8, C		153.4, C		155.9, C		152.2, C	7.25, t (7.7)	124.8, C	7.36, t (7.8)	126.2, C
8		111.0, C	6.94, s	97.6, CH		112.3, C	6.90, s	97.7, CH	7.44, d (7.7)	111.6, CH	7.51, d (8.8)	112.1, CH
9	9.63, br s		10.00, br s		8.79, br s		7.75, br s		9.84, br s		10.26, br s	
8a		140.3, C		141.3, C		141.0, C		139.7, C		140.9, C		141.5, C
9a		136.1, C		136.2, C		138.7, C		135.4, C		136.8, C		135.3, C
1′	6.95, d (9.8)	119.3, CH	6.89, d (9.8)	119.1, CH	3.60, m	24.3, CH_2_	6.62, d (9.8)	117.7, CH		133.4, C		134.3, C
2′	5.72, d (9.8)	128.9, CH	5.73, d (9.8)	129.0, CH	5.28, t (7.1)	123.1, CH	5.65, d (9.8)	128.8, CH	6.83, s	104.8, CH	6.74, s	104.0, CH
3′		78.6, C		78.7, C		136.6, C		78.2, C		148.8, C		148.7, C
4′	1.42, s	26.1, CH_3_	1.43, s	26.2, CH_3_	1.86, s	16.5, CH_3_	1.44, s	26.0, CH_3_		136.8, C		136.4, C
5′	1.75, m	41.4, CH_2_	1.75, t (8.3)	41.6, CH_2_	2.02, m	40.4, CH_2_	1.76, t (8.4)	40.9, CH_2_		148.8, C		148.7, C
6′	2.18, m	23.5, CH_2_	2.18, m	23.5, CH_2_	2.08, m	27.5, CH_2_	2.16, m	22.9, CH_2_	6.83, s	104.8, CH	6.74, s	104.0, CH
7′	5.12, t (7.4)	125,2, CH	5.14, m	125.2, CH	5.07, t (6.8)	125,2, CH	5.11, t (7.3)	124.4, CH	5.44, d (7.7)	87.7, CH	5.89, d (6.0)	88.5, CH
8′		131.9, C		131.9, C		131.7, C		131.8, C	3.88, m	54.7, CH	3.71, m	55.6, CH
9′	1.56, s	17.6, CH_3_	1.56, s	17.6, CH_3_	1.54, s	17.7, CH_3_	1.58, s	17.7, CH_3_	4.01, m 4.07, m	64.8 CH_2_	3.73, m 4.05, d (8.1)	63.9, CH_2_
10′	1.63, s	25.8, CH_3_	1.63, s	25.8, CH_3_	1.58, s	25.8, CH_3_	1.66, s	25.8, CH_3_				
1′′	6.92, br s		6.88, br s		6.98, br s			129.2, C				
2′′		178.6, C		177.9, C		178.7, C	6.74, s	104.1, CH				
3′′	2.36, m 2.49, m	28.1, CH_2_	2.28, m	30.4, CH_2_	2.42, m	31.8, CH_2_		147.4, C				
4′′	2.37, m 2.47, m	31.7, CH_2_	1,99, m 2.61, m	30.5, CH_2_	2.22, m 2.53, m	28.8, CH_2_		135.2, C				
5′′	5.48, t (7.3)	51.8, CH	5.14, m	53.6, CH	5.53, t (7.9)	52.5, CH		147.4, C				
6′′							6.74, s	104.1, CH				
7′′							4.73, d (8.4)	82.8, CH				
8′′							4.17, m	68.9, CH				
9′′							3.09, dd (15.4, 9.4) 3.31, dd (15.4, 5.6)	33.8, CH_2_				
3-CH_3_	2.28, s	16.2, CH_3_	2.29, s	16.2, CH_3_	2.39, s	17.1, CH_3_	2.32, s	16.2, CH_3_	2.37, s	15.8, CH_3_	2.44, s	19.1, CH_3_
7-OCH_3_					3.89, s	56.9, CH_3_						
3′-OCH_3_									3.82, s	56.8, CH_3_	3.73, s	56.7, CH_3_
3′′-OCH_3_							3.90, s	56.9, CH_3_				
5′-OCH_3_									3.82, s	56.8, CH_3_	3.73, s	56.7, CH_3_
5′′-OCH_3_							3.90, s	56.9, CH_3_				

^a^Measured in acetone-*d*_6_

^b^Measured in CDCl_3_

**Fig. 2:**
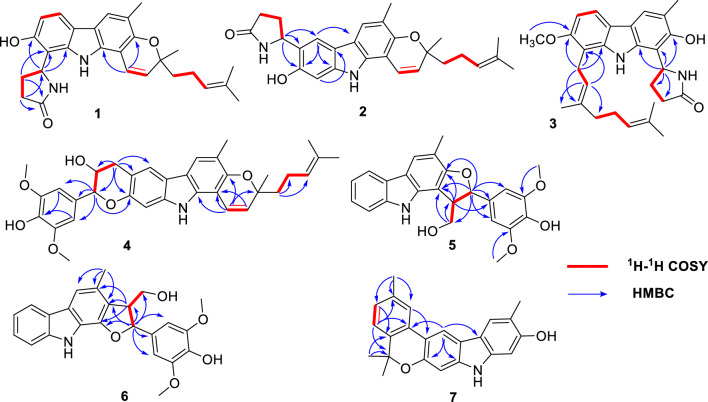
Key HMBC and ^1^H-^1^H COSY correlations of **1**–**7**

The ^1^H and ^13^C NMR data of compound **1** closely resembled those of mahanine [16], with a group of signals corresponding to an additional pyrrolidone unit appearing at *δ*_H_ 2.36 (1H, m, H-3ʹʹa), 2.37 (1H, m, H-4ʹʹa), 2.49 (1H, m, H-3ʹʹb), 2.47 (1H, m, H-4ʹʹb), 5.48 (1H, t, *J* = 7.3 Hz, H-5ʹʹ), and 6.92 (1H, brs, 1ʹʹ-*N*H), with carbon shifts at *δ*_C_ 28.1 (C-3ʹʹ), 31.7 (C-4ʹʹ), 51.8 (C-5ʹʹ), and 178.6 (C-2ʹʹ) [17]. The HMBC data (Fig. 2) revealed correlations between H-4ʹʹ and C-8, as well as between H-5ʹʹ and multiple carbons, namely C-7, C-8a, and C-8. These correlations strongly indicate that the pyrrolidone unit is linked to C-8 of pyranocarbazole. Compound **1** is the first reported natural carbazole alkaloid with a pyrrolidone unit.

**Fig. 3:**
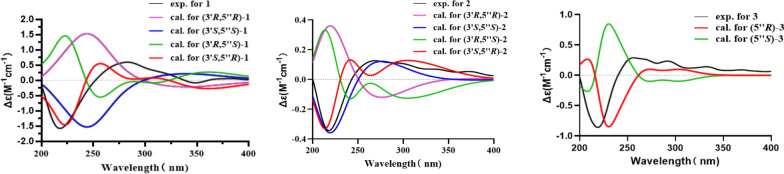
Experimental and calculated ECD spectra of compounds **1** (left), **2** (middle), and **3** (right)

There are two chiral carbons in **1**, thus, the ECD data of four possible configurations were calculated (Fig. 3), and from these data we found that the ECD curve of **1** was mainly contributed by the 3ʹ*S* configuration (Fig. 3), also supported by the similar ECD data with (3ʹ*S*)-mahanine (Fig. S13, Supporting Information), the biogenetic precursor of **1**. However, due to the similarity in the trend of the calculated ECD curves for (3ʹ*S*, 5ʹʹ*R*) and (3ʹ*S*, 5ʹʹ*S*), it is difficult to give a definite answer to the conformation of C-5ʹʹ. Therefore, the quantum chemical calculations of the NMR data of (3ʹ*S*, 5ʹʹ*R*)-**1** and (3ʹ*S*, 5ʹʹ*S*)-**1** were performed. However, the results were still not satisfied to distinguish these two isomers (data not shown). Thus, only the 3ʹ configuration of **1** was defined as *S*, while the 5ʹʹ configuration was undetermined.

Euchrestifoline B (**2**) was obtained as a brown, non-crystalline solid, [*α*]_D_^25^ + 7 (*c* 0.09, MeOH). Its molecular formula, C_27_H_30_N_2_O_3_, matched that of compound **1**, as confirmed by ^13^C NMR and HRESIMS data (*m*/*z* 429.2168 [M − H]^–^, calcd for C_27_H_29_N_2_O_3_, 429.2178). The NMR, UV, and IR characteristics are similar to those of **1**, but a notable distinction was the change from a pair of *ortho*-coupled aromatic doublets in **1** to two aromatic singlets [*δ*_H_ 7.77 (H-5) and *δ*_H_ 6.94 (H-8)] in **2**, indicating a shift in the pyrrolidone unit's attachment within the pyranocarbazole framework. The HMBC spectrum provided crucial correlations from H-5ʹʹ to C-5, C-6, and C-7 (Fig. 2). These correlations unambiguously positioned the pyrrolidone unit at the C-6 position. By comparing the calculated ECD curves of the four configurations of Euchrestifoline B (**2**) with its experimental curve (Fig. 3), the same question in **1** also existed in stereo configuration of **2**, and thus only the 3ʹ*S* configuration of **2** was determined.

Euchrestifoline C (**3**) was afforded as a brown, non-crystalline solid, [*α*]_D_^25^ + 9 (*c* 0.12, MeOH). HRESIMS analysis revealed a molecular ion at* m*/*z* 445.2488 [M − H]^–^ (calcd for C_28_H_33_N_2_O_3_, 445.2491), indicating that the molecular formula of **3** is C_28_H_34_N_2_O_3_. The NMR data of **3** (Table 1) bore resemblance to those of **1** and **2**, with the key distinction being a geranyl moiety [*δ*_H_ 1.58 (H_3_-10ʹ), 1.54 (H_3_-9ʹ), 5.07 (H-7ʹ), 2.08 (H_2_-6ʹ), 2.02 (H_2_-5ʹ), 1.86 (H_3_-4ʹ), 5.28 (H-2ʹ), and 3.60 (H_2_-1ʹ)] replacing the 2-methyl-2-(4-methylpent-3-enyl)-2*H*-pyran moiety in **1** and **2**. Additionally, the presence of a methoxy group in **3** was confirmed by NMR data (*δ*_H_ 3.89; *δ*_C_ 56.9). The HMBC correlations from the methoxy protons to C-7, from H-1ʹ to C-7/C-8a/C-8, and from H-2ʹ to C-8 (Fig. 2) indicated that the methoxy and the geranyl moieties are attached at C-7 and C-8, respectively. Similarly, the localization of the pyrrolidone unit at the C-1 position was deduced through the correlations observed between H-5ʹʹ and the carbon atoms C-1, C-2, and C-9a. The ECD spectrum computed for (5ʹʹ*R*)-**3** exhibited a favorable correspondence with the experimental data (Fig. 3), conclusively confirming the chiral configuration at the C-5ʹʹ position.

Euchrestifoline D (**4**) was isolated as a brown, non-crystalline solid with a specific rotation of [*α*]_D_^25^ + 4 (*c* 0.08, MeOH). The molecular formula C_34_H_37_NO_6_ was confirmed by HRESIMS, which revealed a deprotonated molecular ion at *m*/*z* 554.2550 [M − H]^−^ (calcd for C_34_H_36_NO_6_, 554.2543), and its ^13^C NMR data offered further support for the molecular formula. Comparison of the NMR data of **4** with those of mahanine [16], a set of ABX-coupled aromatic protons found in mahanine were substituted by two singlet signals at *δ*_H_ 7.60 (H-5) and *δ*_H_ 6.90 (H-8) in** 4**. Additionally, the ^1^H NMR spectrum displayed a 3,5-dimethoxy-4-hydroxyphenyl group [*δ*_H_ 6.74 (2H, s, H-2ʹʹ, H-6ʹʹ), 3.90 (6H, s, 3ʹʹ-OCH_3_, 5ʹʹ-OCH_3_)], alongside two methylene protons [*δ*_H_ 3.09 (1H, dd *J* = 15.4, 9.4 Hz, H-9ʹʹa), 3.31 (1H, dd *J* = 15.4, 5.6 Hz, H-9ʹʹb)], and two oxygenated methine protons [*δ*_H_ 4.17 (1H, m, H-8ʹʹ), 4.73 (1H, d *J* = 8.4 Hz, H-7ʹʹ)]. The ^13^C NMR data (Table 1) indicated a total of 34 distinct carbon signals, comprising 23 carbon signals from the mahanine unit, two from methoxy groups, six from aromatic carbons, and three from aliphatic carbons. By analyzing the ^1^H‒^1^H COSY relationships of H-9ʹʹ/H-8ʹʹ/H-7ʹʹ (Fig. 2) and their respective chemical shifts, a “–CH_2_–CHOH–CHR–O–” fragment was identified. The HMBC correlations of H-7ʹʹ with C-1ʹʹ/C-2ʹʹ/C-6ʹʹ/C-8ʹʹ/C-9ʹʹ, and of the methoxy protons (*δ*_H_ 3.90) with C-3ʹʹ/C-5ʹʹ (*δ*_C_ 147.4), confirmed the presence of a 4-hydroxy-3,5-dimethoxyphenylpropanyl unit in the structure. Given that **4** has 17 degrees of hydrogen deficiency, the existence of another ring was anticipated. In the HMBC spectrum, the correlations (Fig. 2) from H-9ʹʹ to C-5/C-6/C-7/C-7ʹʹ/C-8ʹʹ and from H-7ʹʹ to C-7 further established the attachment of the phenylpropanyl moiety to the mahanine unit via C-6 and C-7 to form a pyran ring. Ultimately, the 2D structure of **4** was characterized, marking it as the first phenylpropanyl-substituted pyranocarbazole to feature a new fused pyran ring.

**Fig. 4:**
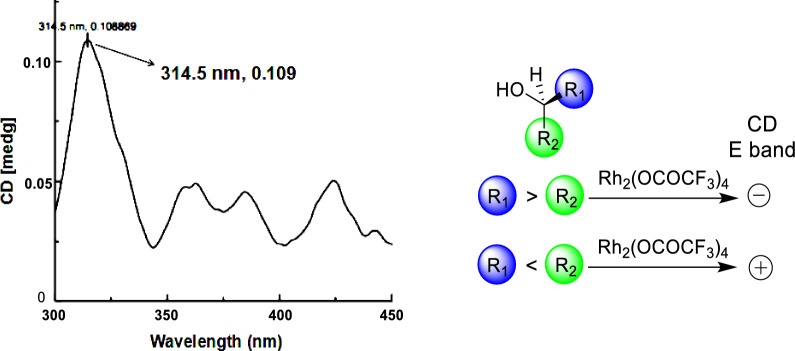
The Rh_2_(OCOCF_3_)_4_ induced CD spectrum of compound **4a** in CHCl_3_

Compound **4** was characterized as a racemic mixture, with its specific rotation value being nearly zero. Furthermore, the ECD spectrum exhibited minimal Cotton effects, also indicating the presence of a racemic composition. Subsequently, it was separated into enantiomers, **4a** and **4b** using chiral-phase HPLC with a mobile phase of *n*-hexane–isopropanol (70:30, *v*/*v*), in a ratio of approximately 1:1 (see Fig. S2, Supporting Information). The specific rotations of compounds **4a** and **4b** were completely opposite (**4a**: + 27; **4b**: − 27) as were their Cotton effects (Fig. S2, Supporting Information). The *trans*-configuration of H-7ʹʹ relative to H-8ʹʹ was inferred from the larger coupling constant, *J*_H-7ʹʹ‒H-8ʹʹ_ (8.4 Hz) [18, 19]. The (8ʹʹ*S*) absolute configuration of **4a** was validated by the observation of a pronounced positive Cotton effect at 314 nm in the ECD spectrum of its Rh_2_(OCOCF_3_)_4_ complex, dissolved in CH_2_Cl_2_ (Fig. 4) [20]. The stereochemistry at the C-3ʹ position in **4a** was determined by comparing the theoretical ECD curves of (3ʹ*R*,7ʹʹ*R*,8ʹʹ*S*-**4**) and (3ʹ*S*,7ʹʹ*S*,8ʹʹ*R*-**4**) with the experimental data, as shown in Fig. S2 of Supporting Information. Thus, the absolute configuration of (+)-euchrestifoline D (**4a**) was designated as (3ʹ*R*,7ʹʹ*R*,8ʹʹ*S*), while (−)-euchrestifoline D (**4b**) was defined as (3ʹ*S*,7ʹʹ*S*,8ʹʹ*R*).

Euchrestifoline E (**5**) was a brown, non-crystalline solid, with a molecular formula of C_24_H_23_NO_5_ determined from its HRESIMS data (*m*/*z* 404.1491 [M − H]^−^, calcd for C_24_H_22_NO_5_, 404.1498) and ^13^C NMR data. In the ^1^H NMR data (Table 1), characteristic signals were observed for *ortho*-disubstituted phenyl protons [*δ*_H_ 7.96 (H-5), 7.10 (H-6), 7.25 (H-7), 7.44 (H-8)] alongside an aromatic singlet at *δ*_H_ 7.75 (H-4) and a methyl singlet at* δ*_H_ 2.37 (3-CH_3_) attributed to the carbazole nucleus. The remaining ^1^H NMR signals were found to be similar to those in the phenylpropanyl group observed in **4**. Through analysis of the ^1^H‒^1^H COSY correlations and the chemical shifts of H-7ʹ (*δ*_H_ 5.44), H-8ʹ (*δ*_H_ 3.88), and H-9ʹ (*δ*_H_ 4.01, 4.07), a “‒OCH_2_‒CHR_1_‒CHR_2_‒O‒” fragment was constructed. HMBC correlations (Fig. 2) from H-7ʹ to C-1/C-2/C-1ʹ/C-2ʹ/C-6ʹ/C-8ʹ/C-9ʹ, from H-8ʹ to C-1/C-2/C-1ʹ/C-7ʹ/C-9ʹ, and from H_2_-9ʹ to C-1/C-7ʹ/C-8ʹ suggested a “C-8ʹ‒C-1, C-7ʹ‒O‒C-2” linkage, forming a furan ring at C-1/C-2 of carbazole. This aligned with the hydrogen deficiency index of 14 of **5**. Consequently, compound** 5**, a phenylpropanyl-substituted carbazole alkaloid, was characterized as described.

Similar to compound **4**,** 5** also existed as a racemate, and compounds **5a** and **5b** were separated by chiral-phase HPLC, displaying opposite specific rotations and Cotton effects (Fig. S3, Supporting Information). The configuration of H-7ʹ relative to H-8ʹ was determined to be *trans* based on their larger coupling constant, *J*_H-7ʹ‒H-8ʹ_ (7.7 Hz) [18, 19], supported by NOE interactions observed between H-7ʹ and H-9ʹ. Subsequent to a comparison of the calculated and experimental ECD spectra of **5a** and **5b** (Fig. S3, Supporting Information), their absolute configurations were deduced as (7′*R*,8′*S*) and (7′*S*,8′*R*), respectively.

Euchrestifoline F (**6**) was obtained as a brown oil. The HRESIMS identified a molecular ion at *m*/*z* 404.1490 [M − H]^−^ (calcd for C_24_H_22_NO_5_, 404.1498), confirming the molecular formula of C_24_H_23_NO_5_, which was the same as that of **5**. Assessment of UV, IR, and NMR data revealed structural similarities between **6** and** 5**. However, the key difference resides in the manner of attachment of the furan ring to the carbazole group. The HMBC analysis indicated C-8ʹ‒C-2 and C-7ʹ‒O‒C-1 linkages for **6**, derived from correlations of H-7ʹ to C-1/C-2, and of H-8′ to C-1/C-2/C-3 (Fig. 2). Thus, the 2D structure of **6** was articulated as shown.

A *trans*-position of H-7ʹ and H-8ʹ was concluded based on their coupling constant, *J*_H-7ʹ‒H-8ʹ_ (6.0 Hz) [18, 19], along with the observed NOE correlation between H-7ʹ and H_2_-9ʹ. Similar to compounds **4** and **5**, the zero specific rotation and chiral-phase HPLC analysis revealed that compound **6** is also a racemic mixture, with a pair of enantiomers **6a** and **6b** in a ratio of approximately 1:1 (Fig. S56, Supporting Information). Unfortunately, due to the small amount remaining, compound** 6** was not subjected to preparative separation.

Euchrestifoline G (**7**) was obtained as a brown, non-crystalline solid. Its molecular formula was confirmed as C_23_H_21_NO_2_ by analysis of a deprotonated molecular ion detected at* m*/*z* 342.1492 [M − H]^−^ (calcd for C_23_H_20_NO_2_, 342.1494) in the HRESIMS, along with the ^13^C NMR data. Its ^1^H NMR data (Table 2) revealed distinct signals characteristic of a carbazole structure, including four aromatic singlets [*δ*_H_ 6.80 (H-1), 7.77 (H-4), 8.24 (H-5), and 6.91 (H-8)], one methyl singlet [*δ*_H_ 2.41 (3-CH_3_)], and an active hydrogen proton [*δ*_H_ 7.76 (H-9)]. Additionally, ABX coupled phenyl signals were observed at *δ*_H_ 7.69 (H-2ʹ), 7.08 (H-4ʹ), and 7.16 (H-5ʹ), alongside three methyl singlets [*δ*_H_ 2.45 (3ʹ-CH_3_), 1.64 (H_3_-8ʹ and H_3_-9ʹ)]. The ^13^C NMR data (Table 2) displayed 23 carbon resonances that included four methyl carbons, one *sp*^3^ quaternary carbon, and 18 aromatic carbons, indicating the presence of three benzene rings in compound **7**.

**Table 2:** ^1^H (500 MHz) and ^13^C (125 MHz) NMR data of **7**–**11** (*δ*_H_ in ppm, *J* in Hz)

No	**7**^a^		**8**^a^		**9**^b^		**10**^a^		**11**^a^	
	*δ*_H_ (*J* in Hz)	*δ*_*C*_, type	*δ*_H_ (*J* in Hz)	*δ*_*C*_, type	*δ*_H_ (*J* in Hz)	*δ*_C_, type	*δ*_H_ (*J* in Hz)	*δ*_C_, type	*δ*_H_ (*J* in Hz)	*δ*_C_, type
1	6.80, s	96.8, CH	6.77, s	97.1, CH	6.93, s	97.4, CH	7.46, d (8.3)	110.8, CH	7.38, d (8.0)	110.6, CH
2		152.5, C		152.4, C		154.7, C	7.89, d (8.3)	126.5, CH	7.35, d (8.0)	124.4, CH
3		116.3, C		116.1, C		117.3, C		129.4, C		132.6, C
4	7.77, s	121.3, CH	7.66, s	121.2, CH	7.65, s	121.5, CH	8.48, s	123.0, CH	7.94, s	118.7, CH
4a		117.8, C		118.1, C		117.6, C		124.4, C		124.3, C
4b		118.7, C		118.2, C		119.1, C		117.3, C		117.5, C
5	8.24, s	113.6, CH	7.73, d (8.4)	117.9, CH	7.70, d (8.4)	117.5, CH	7.83, d (8.3)	119.3, CH	7.77, d (8.4)	120.7, CH
6		116.1, C	6.81, d (8.4)	104.1, CH	6.82, d (8.4)	105.0, CH	6.83, d (8.3)	110.7, CH	6.74, d (8.4)	110.0, CH
7		151.6, C		155.4, C		155.6, C		153.3, C		152.1, C
8	6.91, s	99.3, CH		109.4, C		112.3, C		109.2, C		104.8, C
9	7.76, br s		8.48, br s		9.55, br s		8.29, br s		7.96, br s	
8a		140.9, C		141.8, C		141.0, C		140.9, C		136.8, C
9a		139.7, C		140.2, C		141.4, C		143.6, C		139.3, C
1′		129.9, C	2.91, dd (14.3, 8.4) 3.34, dd (14.3, 2.5)	32.4, CH_2_	3.61, d (6.6)	24.6, CH_2_	3.65, d (7.0)	24.4, CH_2_	6.66, d (9.8)	117.3, CH
2′	7.69, br s	122.8, CH	4.41, dd (8.4, 2.5)	76.1, CH	5.36, d (6.6)	123.4, CH	5.39, t (7.0)	121.2, CH	5.68, d (9.8)	129.0, CH
3′		137.4, C		152.3, C		136.3, C		139.2, C		78.6, C
4′	7.08, d (7.9)	127.6, CH	4.91, s; 5.13, s	108.9, CH_2_	1.83, s	16.5, CH_3_	1.91, s	16.7, CH_3_	1.46, s	26.2, CH_3_
5′	7.16, d (7.9)	123.2, CH	2.23, m	32.6, CH_2_	2.01, m; 2.35, m	37.8, CH_2_	2.10‒2.15, m	39.8, CH_2_	1.76, m	41.0, CH_2_
6′		136.5, C	2.23, m	26.8, CH_2_	1.31, m; 1.67, m	30.8, CH_2_	2.10‒2.15, m	26.6, CH_2_	2.17, m	22.9, CH_2_
7′		77.8, C	5.18, t (6.6)	124.2, CH	3.23, d (10.9)	78.6, CH	5.06, m	123.8, CH	5.11, t (7.5)	124.2, CH
8′	1.64, s	27.7, CH_3_		132.1, C		72.8, C		132.3, C		131.9, C
9′	1.64, s	27.7, CH_3_	1.64, s	17.9, CH_3_	1.08, s	25.2, CH_3_	1.59, s	17.9, CH_3_	1.58, s	17.8, CH_3_
10′			1.71, s	25.9, CH_3_	1.08, s	25.9, CH_3_	1.64, s	25.8, CH_3_	1.66, s	25.8, CH_3_
3-CH_3_	2.41, s	16.3, CH_3_	2.38, s	16.3, CH_3_	2.32, s	16.7, CH_3_				
3′-CH_3_	2.45, s	21.6, CH_3_								
3-CHO							10.07, s	192.2, CH		
3-CH_2_OH									4.83, s	66.3, CH_2_
7-OCH_3_			3.90, s	56.5, CH_3_	3.87, s	56.9, CH_3_				

^a^Measured in CDCl_3_

^b^Measured in acetone-*d*_6_

In HMBC spectrum, the correlations (Fig. 2) from 3ʹ-CH_3_ to C-2ʹ/C-3ʹ/C-4ʹ and from 8ʹ-CH_3_/9ʹ-CH_3_ to C-6ʹ (*δ*_C_ 136.5) and C-7ʹ (*δ*_C_ 77.8) suggested that a methyl group was attached to C-3ʹ and an isopropyl group was presented at C-6ʹ of the phenyl segment. Furthermore, there were HMBC correlations between H-5 and C-1ʹ, and H-2ʹ and C-6, suggesting a linkage between the phenyl group and the carbazole core via C-1ʹ–C-6. Additionally, the formation of a pyran ring was inferred due to a C-7ʹ–*O*–C-7 linkage, identified by the hydrogen deficiency index of 14 for **7** and the lack of a proton at H-7. Thus, the structure of euchrestifoline G (**7**), a novel benzopyranocarbazole alkaloid, was characterized as depicted.

**Fig. 5:**
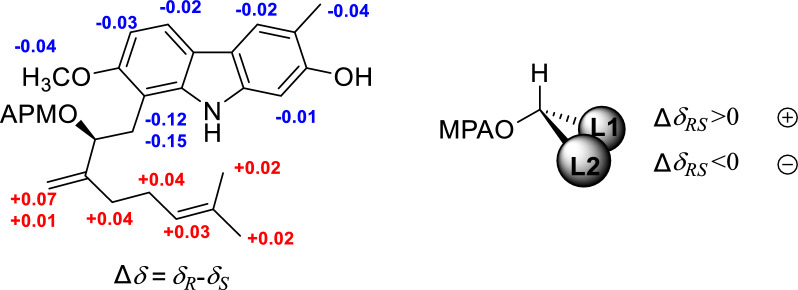
Δ*δ* = *δ*_*R*_*-δ*_*S*_ values obtained from the ^1^H NMR data of the MPA esters of **8**

Euchrestifoline H (**8**) was similarly obtained as a brown, non-crystalline solid, [*α*]_D_^25^ + 11 (*c* 0.14, MeOH). Its molecular formula was determined to be C_24_H_29_NO_3_, confirmed by a quasimolecular ion at *m*/*z* 378.2072 [M − H]^−^ (calcd for C_24_H_28_NO_3_, 378.2069). Analysis of the UV, IR, and NMR data (Table 2) revealed that the structure of **8** closely resembled that of euchrestine B [21]. The distinction lied in the oxidation of H-2′ of the geranyl group in euchrestine B to a hydroxy group in **8**, alongside a shift of the olefinic double bond from C-2ʹ–C-3′ to C-3ʹ–C-4′ [*δ*_H_ 4.41 (H-2ʹ), 4.91 (H-4ʹa), 5.13 (H-4ʹb); *δ*_C_ 76.1 (C-2ʹ), 152.3 (C-3ʹ), 108.9 (C-4ʹ)]. The HMBC correlations from H-1ʹ to C-7/C-8/C-8a/C-2ʹ/C-3ʹ, from H-2ʹ to C-8/C-1ʹ/C-4ʹ/C-5ʹ, and from H-4ʹ to C-2ʹ/C-5ʹ (Fig. S1, Supporting Information) further supported this conclusion. The (2ʹ*S*) absolute configuration was established through Mosher ester analysis (Fig. 5) [22]. Ultimately, the structure of euchrestifoline H (**8**) was confirmed as illustrated.

**Fig. 6:**
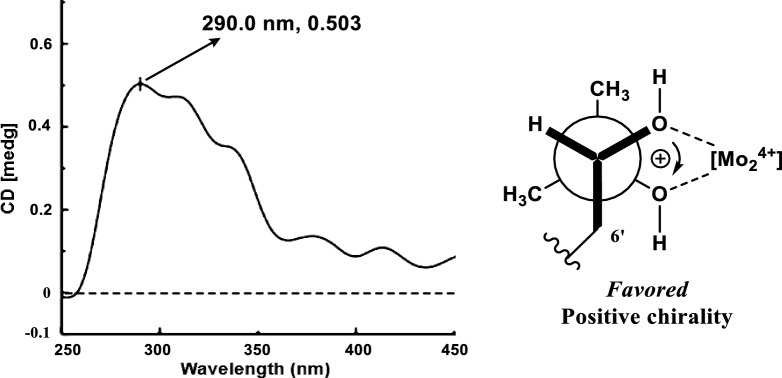
Mo_2_(OAc)_4_-induced CD spectrum for **9** and Newman projection of the diol moiety of **9**, with the helicity rule applied

Euchrestifoline I (**9**) was also obtained as a brown, non-crystalline solid, [α]_D_^25^ + 15 (*c* 0.14, MeOH). Its molecular formula was defined as C_24_H_31_NO_4_ based on a deprotonated molecular ion at *m*/*z* 396.2167 [M − H]^–^ (calcd for C_24_H_30_NO_4_, 396.2175) in the negative-ion HRESIMS and ^13^C NMR data. Comparison of NMR data of **9** (Table 2) with those of euchrestine B [21] indicated an oxidation of the double bond between C-7ʹ and C-8ʹ in euchrestine B to a dihydroxy group [*δ*_H_ 3.23 (H-7ʹ); *δ*_C_ 78.6 (C-7ʹ), 72.8 (C-8ʹ)] in **9**. The 2D configuration of **9** was established as depicted, supported by relevant HMBC correlations (Fig. S1, Supporting Information). In the ECD spectrum of its Mo_2_(OAc)_4_ complex in DMSO, a significant positive Cotton effect was observed at 290 nm, from which the absolute configuration of **9** was deduced to be 7ʹ*S* (Fig. 6) [23].

Euchrestifoline J (**10**) was isolated as a brown, non-crystalline solid. The HRESIMS analysis presented a deprotonated molecular ion at *m*/*z* 346.1807 [M − H]^−^ (calcd for C_23_H_24_NO_2_, 346.1807), which corresponded to a molecular formula of C_23_H_25_NO_2_, corroborated by the ^13^C NMR data. The NMR characteristics of compound **10** (Table 2) closely mirrored those of euchrestine C [24], with notable differences such as the absence of a 2-OH group and the substitution of a 3-CH_3_ with a 3-CHO group (*δ*_H_ 10.07; *δ*_C_ 192.2). The structure of euchrestifoline J was thereby confirmed alongside the HMBC correlations (Fig. S1, Supporting Information).

Euchrestifoline K (**11**) was similarly purified as a brown, non-crystalline solid. Its molecular formula, C_23_H_25_NO_2_, was supported by HRESIMS revealing a deprotonated molecular ion at *m*/*z* 346.1802 [M − H]^−^ (calcd for C_23_H_24_NO_2_, 346.1807). The NMR data (Table 2) exhibited high similarities to the structure of mahanimbicine [25], with the distinction that a methyl singlet in mahanimbicine was replaced by a hydroxymethyl group (*δ*_H_ 4.83, *δ*_C_ 66.3) in **11**. Additional 2D NMR investigations further confirmed the structure of euchrestifoline K as depicted. By comparison with (3ʹ*S*)-pyrayafoline D [26], the (3ʹ*S*) absolute configuration of **11** was established by its corresponding optical rotation and similar ECD curve (Fig. S95, Supporting Information).

Euchrestifoline L (**12**) was isolated as a brown, non-crystalline solid. The HRESIMS revealed a deprotonated molecular ion at *m*/*z* 226.0867 [M − H]^−^ (calcd for C_14_H_12_NO_2_, 226.0868), which matched the calculated formula for C_14_H_13_NO_2_. A comparison of its NMR data (Table 3) with that of 2-hydroxy-3-methylcarbazole [27] indicated that a methoxy group (*δ*_H_ 4.01) in compound **12** substituted the H-1 proton of 2-hydroxy-3-methylcarbazole. This substitution was also supported by HMBC correlations (Fig. S1, Supporting Information) from the methoxy protons to C-1 (*δ*_C_ 131.3). Consequently, euchrestifoline L (**12**) was elucidated as 2-hydroxy-1-methoxy-3-methylcarbazole.

**Table 3:** ^1^H (500 MHz) and ^13^C (125 MHz) NMR data of **12**–**15** (*δ*_H_ in ppm, *J* in Hz)

No	**12**^a^		**13**^b^		**14**^b^		**15**^b^	
	*δ*_H_ (*J* in Hz)	*δ*_*C*_, type	*δ*_H_ (*J* in Hz)	*δ*_*C*_, type	*δ*_H_ (*J* in Hz)	*δ*_C_, type	*δ*_H_ (*J* in Hz)	*δ*_C_, type
1		131.3, C		144.6, C	6.85, s	97.0, CH	6.96, s	97.4, CH
2		145.4, C	7.42, s	108.6, CH		154.0, C		154.9, C
3		118.0, C		131.3, C		116.8, C		117.5, C
4	7.57, s	117.1, CH	8.22, s	119.3, CH	7.60, s	121.1, CH	7.66, s	121.7, CH
4a		118.1, C		125.8, C		117.5, C		117.5, C
4b		124.2, C		126.4, C		117.5, C		120.7, C
5	7.94, d (7.7)	119.7, CH	7.70, d (7.7)	112.7, CH	7.60, s	120.1, CH	7.56, d (8.4)	114.7, CH
6	7.20, t (7.7)	119.8, CH	7.11, t (7.7)	121.8, CH		120.7, C	6.84, d (8.4)	106.9, CH
7	7.33, t (7.7)	124.7, C	6.96, d (7.7)	112.1, CH		153.6, C		150.2, C
8	7.40, d (7.7)	110.7, C		144.4, C	6.86, s	97.1, CH		135.0, C
9	7.94, br s		10.46, br s				9.82, br s	
8a		139.8, C		130.8, C		140.6, C		135.1, C
9a		131.1, C		134.5, C		140.4, C		141.3, C
1′					3.43, d (7.4)	29.5, CH_2_		
2′					5.44, t (7.4)	125.1, CH		
3′						131.5, C		
4′					1.74, s	26.0, CH_3_		
5′					1.76, s	17.9, CH_3_		
3-CH_3_	2.43, s	16.3, CH_3_			2.31, s	16.7, CH_3_	2.32, s	16.7, CH_3_
3-CHO			10.01, s	191.8, CH				
1-OCH_3_	4.01, s	60.9, CH_3_						
7-OCH_3_							3.90, s	60.7, CH_3_
8-OCH_3_							3.92, s	57.3, CH_3_

^a^Measured in CDCl_3_

^b^Measured in acetone-*d*_6_

Euchrestifoline M (**13**), also a brown, non-crystalline solid, was identified with a molecular formula of C_13_H_9_NO_3_ based on the data from ^13^C NMR and HRESIMS (*m*/*z* 226.0507 [M − H]^−^, calcd for C_13_H_8_NO_3_, 226.0504). A comparison of the MS and NMR data for **13** (Table 3) with those of *O*-demethylmurrayanine [28] revealed that **13** has a mass increase of 16 Da and *ortho*-disubstituted phenyl protons in *O*-demethylmurrayanine shifts to *ortho*-trisubstituted phenyl signals at *δ*_H_ 7.70 (H-5), 7.11 (H-6), and 6.96 (H-7) in **13**, indicating the presence of an additional hydroxy group. Based on the corresponding HMBC correlations (Fig. S1, Supporting Information), the hydroxy group was determined to be at C-8. Therefore, euchrestifoline M (**13**) was characterized as 1,8-dihydroxy-3-formylcarbazole.

The molecular formula of euchrestifoline N (**14**) was determined to be C_18_H_19_NO_2_ based on analysis of the ^13^C NMR and the negative-ion HRESIMS data (*m*/*z* 280.1337 [M − H]^−^, calcd for C_18_H_18_NO_2_, 280.1337). The NMR data of **14** (Table 3) displayed similarities to those of euchrestine A [24], with the exception that the prenyl group shifted from C-8 to C-6, as inferred from the presence of two aromatic singlets [*δ*_H_ 7.60, 6.86] in **14** and supported by HMBC correlations (Fig. S1, Supporting Information). Thus, the structure of euchrestifoline N (**14**) was designated as 2,7-dihydroxy-3-methyl-6-prenylcarbazole.

Euchrestifoline O (**15**) was isolated as a brown, non-crystalline solid. Its HRESIMS data revealed a deprotonated molecular ion at *m*/*z* 226.0507 [M − H]^−^ (calcd for C_13_H_8_NO_3_, 226.0504), consistent with the molecular formula of C_13_H_9_NO_3_, further confirmed by the ^13^C NMR data. The NMR characteristics of **15** showed strong resemblance to 2-hydroxy-3-methylcarbazole [27], with the notable addition of two methoxy groups (*δ*_H_ 3.92, 3.90) in **15**. The presence of *ortho*-coupled aromatic doublets [*δ*_H_ 6.84 (H-6), 7.56 (H-5)] in the ^1^H NMR spectrum, along with HMBC correlations, identified these two methoxy groups at C-7 and C-8, respectively (Fig. S1, Supporting Information). Therefore, the structure of euchrestifoline O (**15**) was identified as 2-hydroxy-7,8-dimethoxy-3-methylcarbazole.

**Scheme 1:**
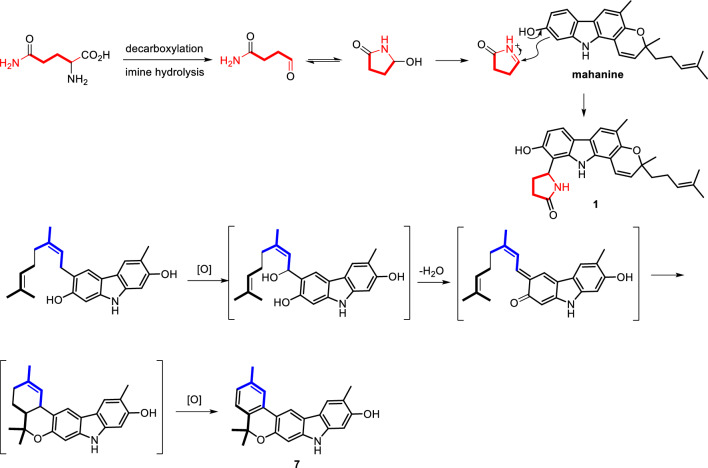
Putative biosynthetic pathways for **1** and **7**

Among the new compounds, **1**–**3** represent a distinctive group of carbazole alkaloids incorporating a pyrrolidone unit. In Scheme 1, the proposed biosynthetic pathways for compounds **1** and **7** are illustrated. It is theorized that compound **1** is derived from mahanine, a well-known carbazole alkaloid found in *Murraya* species, in conjunction with a pyrrolidone iminium ion moiety that is generated from glutamine through decarboxylation, imine hydrolysis, and subsequent cyclization. Additionally, as depicted in Scheme 1, a new benzopyranocarbazole alkaloid structure, represented by compound **7**, is believed to be formed via an intramolecular hetero-Diels–Alder reaction between the in situ generated *ortho*-quinomethide and an adjacent double bond, followed by oxidative aromatization.

### Investigation of NO inhibitory activity

Taking into account the well-established anti-inflammatory and pain-relieving properties of *M. euchrestifolia*, compounds **1**–**15** were assessed for their capacity to inhibit NO production stimulated by LPS in RAW 264.7 cells. As summarized in Table 4, compounds **4a**, **4b**, **6**, and **11**–**14** exhibited strong inhibitory effects, with IC_50_ values below 20 μM. Meanwhile, compounds **1**, **5a**, **5b**, **8**–**10**, and **15** showed moderate inhibitory activity, with their IC_50_ values in the range of 21.6 to 32.5 μM. It is worth noting that the position of the pyrrolidone substituent may impact anti-inflammatory activity (for instance, comparing compounds **1** and** 2**), while isomerization appeared to have a minimal effect on activity (e.g., **4a** compared to **4b** and **5a** compared to **5b**). No isolates presented significant cytotoxicity at 50 μM.

**Table 4:** Various activity screening of **1–15**

Compd	IC_50_ (μM)^a^		EC_50_ (µM)
	NO inhibition	Cytotoxicity	Anti-ferroptosis
**1**	27.7 ± 1.9	18.5 ± 2.6	1.21 ± 0.09
**2**	> 50	30.5 ± 0.8	0.46 ± 0.06
**3**	> 50	34.0 ± 2.7	0.24 ± 0.05
**4a**	18.6 ± 1.1	2.6 ± 0.5	2.20 ± 0.18
**4b**	16.0 ± 0.1	15.0 ± 2.1	
**5a**	21.7 ± 1.2	3.4 ± 0.7	0.42 ± 0.04
**5b**	24.6 ± 1.3	3.4 ± 0.6	
**6**	13.0 ± 1.7	1.2 ± 1.1	0.19 ± 0.03
**7**	> 50	15.9 ± 2.4	9.18 ± 0.40
**8**	32.5 ± 0.7	2.7 ± 0.2	0.23 ± 0.06
**9**	21.6 ± 3.2	25.7 ± 2.0	0.04 ± 0.01
**10**	23.0 ± 0.9	30.0 ± 2.9	17.09 ± 4.09
**11**	19.0 ± 2.8	> 50	0.15 ± 0.08
**12**	16.0 ± 2.1	> 50	4.23 ± 0.26
**13**	12.7 ± 0.8	> 50	1.18 ± 0.17
**14**	19.7 ± 0.1	41.4 ± 2.0	0.63 ± 0.05
**15**	26.3 ± 1.9	> 50	4.91 ± 0.14
**Dexamethasone**^b^	10.1 ± 0.4		
**Taxol**^b^		0.032 ± 0.014	
**Ferrostatin-1**^b^			1.33 ± 0.16

^a^IC_50_ values are presented as mean ± SD (*n* = 3)

^b^Positive control

### Evaluations of cytotoxic activity

The cytotoxic effects of the isolated carbazole alkaloids were evaluated, drawing from findings in the literature [29]. The data presented in Table 4 illustrated the cytotoxic effects of compounds **1**–**10** and **14** on HepG2 cells, with IC_50_ values ranging from 1.2 to 41.4 μM. Notably, compounds **4a**, **5a**, **5b**, **6**, and **8** exhibited considerable cytotoxic effects on HepG2 cells, each with IC_50_ values below 4.0 μM. The presence of a phenylpropanyl substituent appears to enhance the cytotoxicity of these carbazole derivatives, as all phenylpropanyl-substituted carbazoles (**4**–**6**) demonstrated superior cytotoxic abilities compared to other carbazole compounds. For the phenylpropanyl-substituted pyranocarbazole, isomerization may significantly influence cytotoxicity, as seen from **4a** versus **4b**.

### Evaluations of anti-ferroptosis effects

Carbazole alkaloids have been reported to present potent neuro-protection activities [30], and recently anti-ferroptosis has been disclosed to be an important pathway for neuroprotection [31], thus these compounds were assessed for their anti-ferroptosis effects against erastin-induced ferroptosis in PC12 cells. As summarized in Table 4, all compounds provided notable protection, exhibiting EC_50_ values spanning from 0.04 to 17.09 μM, most surpassing the positive control, ferrostatin-1 (EC_50_: 1.33 μM). Compound **9** exhibited the highest potency, with an EC_50_ of 40 nM, suggesting that the *ortho*-dihydroxy group on the geranyl derivative is crucial for its activity. Conversely, the presence of an aldehyde group or a rigid ring structure, such as a benzopyranocarbazole, appears to diminish activity, as illustrated by compounds **10** (EC_50_: 17.09 μM) and** 7** (EC_50_: 9.18 μM).

## Materials and methods

### General experimental procedures

The reagents and instruments used for isolation, purification and structural elucidation of compounds were in accordance with those used in the literature [26]. The analytical grade solvents were utilized for CC, while those of chromatography grade for HPLC.

### Plant materials

The dried leaves and twigs of the plant were collected in May 2016 in Jingxi County, Guangxi Province of China. Prof. P.-F. Tu, a co-author of this study identified them as *Murraya euchrestifolia* Hayata. A reference sample, designated as No. DYJLX201605, was deposited in the Herbarium of Modern Research Center for Traditional Chinese Medicine, Peking University.

### Extraction and isolation processes

Air-dried leaves and twigs of *M. euchrestifolia* (10 kg) were pulverised and extracted with 95% ethanol (100 L) thrice, and each for 2 h. The dried extract (450 g) was obtained under reduced pressure, and then, it was dissolved in water and extracted with CH_2_Cl_2_ to give an extract of 150 g. This extract was processed through silica gel CC, employing a stepwise elution of petroleum ether-acetone (10:1, 5:1, 1:1, and 0:1, *v*/*v*) to yield six distinct fractions (Frs. 1–6).

Fr. 4 (42 g) was treated with Sephadex LH-20 CC eluting with CH_2_Cl_2_–MeOH (1:1, *v*/*v*) and five subfractions, Frs. 4a–4e, were obtained. Fr. 4d was divided into seven subfractions (Frs. 4d1–4d7) by ODS CC (gradient MeOH–H_2_O, 50:50–100:0, *v*/*v*). Fr. 4d3 underwent purification via semi-preparative HPLC with MeCN–H_2_O (47:53, *v*/*v*), eluting at 3 mL/min to obtain **12** (4.0 mg, *t*_R_ 14.2 min). Fr. 4d4 was subjected to a similar semi-preparative HPLC process with a different eluent composition of MeCN–H_2_O (75:25, *v*/*v*, 3 mL/min) to give **8** (2.3 mg, *t*_R_ 7.1 min) and **10** (3.1 mg, *t*_R_ 9.2 min).

Fr. 5 (35 g) was purified by Sephadex LH-20 CC (CH_2_Cl_2_–MeOH, 1:1, *v*/*v*) to yield Frs. 5a–5f. Fr. 5b underwent a gradient elution process utilizing MCI CC with a methanol–water solvent system ranging from 30:70 to 100:0 (*v*/*v*), which led to the acquisition of six subfractions (Frs. 5b1–5b6). Fr. 5b5 was further purified by using MeCN–H_2_O (45:55, *v*/*v*, 3 mL/min) as eluent on a semi-preparative HPLC to afford compounds **15** (3.0 mg, *t*_R_ 6.2 min) and **7** (3.2 mg, *t*_R_ 13.8 min). Fractions (5c1–5c5) were obtained from subfraction 5c by ODS CC, utilizing a gradient elution of methanol and water (*v*/*v*) from 50:50 to 100:0. Compounds **3** (3.4 mg, *t*_R_ 9.2 min) and **4** (3.7 mg, *t*_R_ 12.6 min) were purified from Fr. 5c2 by semi-preparative HPLC, employing a solvent system of acetonitrile and water in 80:20 at a flow rate of 3 mL/min. Fr. 5c3 was further purified with a mobile phase of MeCN–H_2_O (45:55, *v*/*v*, 3 mL/min) to obtain **5** (2.3 mg, *t*_R_ 6.9 min), **6** (2.4 mg, *t*_R_ 7.7 min), and **9** (3.4 mg, *t*_R_ 8.3 min). Fr. 5c5 was further treated with semi-preparative HPLC (MeCN–H_2_O, 55:45, *v*/*v*) at a flow rate of 3 mL/min to obtain **11** (1.6 mg, *t*_R_ 4.0 min), **13** (3.2 mg, *t*_R_ 4.7 min), and **14** (3.7 mg, *t*_R_ 5.1 min).

Fr. 6 (19 g) was treated with Sephadex LH-20 under the same conditions as Fr. 4 and Fr. 5 to obtain six subfractions, 6a–6f. By ODS CC, subfraction 6c was treated with MeOH–H_2_O gradient elution (30:70–100:0, *v*/*v*) to obtain six fractions (6c1–6c6). Based on semi-preparative HPLC, Frs. 6c4 and 6c6 were purified with different gradients of MeCN–H_2_O (60:40 and 70:30, respectively, *v*/*v*, 3 mL/min) to obtain **1** (3.0 mg, *t*_R_ 12.2 min) and **2** (1.5 mg, *t*_R_ 14.0 min), respectively. A Chiralpak AD-H column was used on the semi-preparative HPLC for the enantioseparation of **4a**/**4b** and **5a**/**5b**. Under the chromatographic conditions of *n*-hexane–*i*PrOH (70:30, *v*/*v*, 1 mL/min) and 238 nm detection wavelength, compounds **4a** (1.6 mg, *t*_R_ 20.7 min) and **4b** (1.7 mg, *t*_R_ 24.5 min), and **5a** (1.0 mg, *t*_R_ 13.3 min) and **5b** (1.1 mg, *t*_R_ 15.7 min) were obtained, respectively.

Euchrestifoline A (**1**): brown, non-crystalline solid; [*α*]_D_^25^ + 20 (*c* 0.06, MeOH); UV (MeOH) *λ*_max_ (log *ε*) 221 (4.39), 241 (4.40), 296 (4.16), 312 (3.94) nm; ECD (MeOH) *λ*_max_ (Δ*ε*) 217 (− 0.68), 282 (+ 0.26) nm; IR (KBr) *ν*_max_ 3365, 2976, 2931, 2154, 1713, 1648, 1612, 1517, 1453, 1367, 1252, 1164, 1032, 1019, 929, 857, 762, 578 cm^–1^; ^1^H and ^13^C NMR data, see Table 1; HRESIMS *m*/*z* 429.2169 [M − H]^−^ (calcd for C_27_H_29_N_2_O_3_, 429.2178).

Euchrestifoline B (**2**): brown, non-crystalline solid; [*α*]_D_^25^ + 7 (*c* 0.09, MeOH); UV (MeOH) *λ*_max_ (log *ε*) 225 (4.28), 240 (4.33), 297 (4.05) nm; ECD (MeOH) *λ*_max_ (Δ*ε*) 220 (− 0.84), 256 (+ 0.29) nm; IR (KBr) *ν*_max_ 3364, 2975, 2929, 2154, 1713, 1517, 1367, 1253, 1163, 1021, 9230, 767, 576 cm^–1^; ^1^H and ^13^C NMR data, see Table 1; HRESIMS *m*/*z* 429.2168 [M − H]^−^ (calcd for C_27_H_29_N_2_O_3_, 429.2178).

Euchrestifoline C (**3**): brown, non-crystalline solid; [*α*]_D_^25^ + 9 (*c* 0.12, MeOH); UV (MeOH) *λ*_max_ (log *ε*) 218 (4.21), 239 (4.27), 264 (4.05), 309 (3.83) nm; ECD (MeOH) *λ*_max_ (Δ*ε*) 220 (− 0.62), 268 (+ 0.23) nm; IR (KBr) *ν*_max_ 3376, 2970, 2922, 2859, 1737, 1722, 1616, 1457, 1367, 1217, 1176, 1052, 1032, 1018, 884, 578 cm^–1^; ^1^H and ^13^C NMR data, see Table 1; HRESIMS *m*/*z* 445.2488 [M − H]^−^ (calcd for C_28_H_33_N_2_O_3_, 445.2491).

Euchrestifoline D (**4**): brown, non-crystalline solid; UV (MeOH) *λ*_max_ (log *ε*) 209 (4.39), 241 (4.34), 299 (4.03), 357 (3.46) nm; IR (KBr) *ν*_max_ 3385, 2922, 2852, 1700, 1618, 1464, 1300, 1273, 1153,1025, 835, 575 cm^–1^; ^1^H and ^13^C NMR data, see Table 1; HRESIMS *m*/*z* 554.2550 [M − H]^−^ (calcd for C_34_H_36_NO_6_, 554.2543).

(+)-Euchrestifoline D (**4a**): [*α*]_D_^25^ + 27 (*c* 0.01, MeOH); ECD (MeOH) *λ*_max_ (Δ*ε*) 221 (‒0.88), 247 (+ 1.08), 289 (‒0.35), 330 (+ 0.29) nm.

(−)-Euchrestifoline D (**4b**): [*α*]_D_^25^ − 27 (*c* 0.01, MeOH); ECD (MeOH) *λ*_max_ (Δ*ε*) 222 (+ 1.77), 252 (− 0.57), 231 (+ 0.66), 330 (− 0.13) nm.

Euchrestifoline E (**5**): brown, non-crystalline solid; UV (MeOH) *λ*_max_ (log *ε*) 209 (4.34), 241 (4.28), 256 (4.10), 306 (3.80) nm; IR (KBr) *ν*_max_ 3413, 3004, 2917, 2849, 1713, 1422, 1362, 1222, 1029, 530 cm^–1^; ^1^H and ^13^C NMR data, see Table 1; HRESIMS *m*/*z* 404.1491 [M − H]^−^ (calcd for C_24_H_22_NO_5_, 404.1498).

(+)-Euchrestifoline E (**5a**): [*α*]_D_^25^ + 46 (*c* 0.05, MeOH); ECD (MeOH) *λ*_max_ (Δ*ε*) 201 (‒17.09), 252 (+ 6.57), 231 (+ 2.27) nm, 245 (+ 7.21), 304 (+ 3.44) nm.

(−)-Euchrestifoline E (**5b**): [*α*]_D_^25^ − 46 (*c* 0.07, MeOH); ECD (MeOH) *λ*_max_ (Δ*ε*) 200 (+ 13.60), 252 (− 7.03), 231 (− 2.75) nm, 245 (− 5.20), 304 (− 2.44) nm.

Euchrestifoline F (**6**): brown oil; UV (MeOH) *λ*_max_ (log *ε*) 208 (4.26), 248 (4.24), 294 (3.74) nm; ECD (MeOH) *λ*_max_ (Δ*ε*) 216 (− 2.32) nm; IR (KBr) *ν*_max_ 3385, 2922, 2851, 1706, 1613, 1517, 1222, 1160, 1021, 529 cm^–1^; ^1^H and ^13^C NMR data, see Table 1; HRESIMS *m*/*z* 404.1490 [M − H]^−^ (calcd for C_24_H_22_NO_5_, 404.1498).

Euchrestifoline G (**7**): brown, non-crystalline solid; UV (MeOH) *λ*_max_ (log *ε*) 208 (4.12), 244 (4.15), 306 (4.16), 358 (3.76) nm; IR (KBr) *ν*_max_ 3375, 2921, 2852, 1706, 1613, 1454, 1294, 1221, 1154, 1122, 1018, 578 cm^–1^; ^1^H and ^13^C NMR data, see Table 2; HRESIMS *m*/*z* 342.1492 [M − H]^−^ (calcd for C_23_H_20_NO_2_, 342.1494).

Euchrestifoline H (**8**): brown, non-crystalline solid; [*α*]_D_^25^ + 11 (*c* 0.14, MeOH); UV (MeOH) *λ*_max_ (log *ε*) 214 (4.23), 238 (4.29), 265 (4.07), 309 (3.88) nm; ECD (MeOH) *λ*_max_ (Δ*ε*) 216 (− 0.16) nm; IR (KBr) *ν*_max_ 3386, 2920, 2851, 2154, 1714, 1613, 1452, 1383, 1366, 1162, 1138, 1020, 578 cm^–1^; ^1^H and ^13^C NMR data, see Table 2; HRESIMS *m*/*z* 378.2072 [M − H]^−^ (calcd for C_24_H_28_NO_3_, 378.2069).

Euchrestifoline I (**9**): brown, non-crystalline solid; [*α*]_D_^25^ + 15 (*c* 0.14, MeOH); UV (MeOH) *λ*_max_ (log *ε*) 213 (4.31), 238 (4.40), 309 (3.92) nm; ECD (MeOH) *λ*_max_ (Δ*ε*) 211 (− 1.33), 255 (0.37) nm; IR (KBr) *ν*_max_ 3381, 2923, 2852, 1705, 1617, 1454, 1262, 1223, 1162, 1021, 577 cm^–1^; ^1^H and ^13^C NMR data, see Table 2; HRESIMS *m*/*z* 396.2167 [M − H]^−^ (calcd for C_24_H_30_NO_4_, 396.2175).

Euchrestifoline J (**10**): brown, non-crystalline solid; UV (MeOH) *λ*_max_ (log *ε*) 205 (3.90), 242 (3.99), 293 (4.07) nm; IR (KBr) *ν*_max_ 3356, 2970, 2920, 2851, 1737, 1722, 1366, 1228, 1216, 1038, 1025, 577 cm^–1^; ^1^H and ^13^C NMR data, see Table 2; HRESIMS *m*/*z* 346.1807 [M − H]^−^ (calcd for C_23_H_24_NO_2_, 346.1807).

Euchrestifoline K (**11**): brown, non-crystalline solid; [*α*]_D_^25^ − 12 (*c* 0.05, MeOH); UV (MeOH) *λ*_max_ (log *ε*) 239 (4.26), 288 (4.13) nm; ECD (MeOH) *λ*_max_ (Δ*ε*) 229 (− 0.81) nm; IR (KBr) *ν*_max_ 3420, 2921, 1706, 1611, 1453, 1163, 1021, 579, 448 cm^–1^; ^1^H and ^13^C NMR data, see Table 2; HRESIMS *m*/*z* 346.1802 [M − H]^−^ (calcd for C_23_H_24_NO_2_, 346.1807).

Euchrestifoline L (**12**): brown, non-crystalline solid; UV (MeOH) *λ*_max_ (log *ε*) 216 (3.93), 238 (4.04), 300 (3.65) nm; IR (KBr) *ν*_max_ 3385, 2919, 2850, 1704, 1637, 1614, 1463, 1316, 1198, 1074, 1019, 1007, 742 cm^–1^; ^1^H and ^13^C NMR data, see Table 3; HRESIMS *m*/*z* 226.0867 [M − H]^−^ (calcd for C_14_H_12_NO_2_, 226.0868).

Euchrestifoline M (**13**): brown, non-crystalline solid; UV (MeOH) *λ*_max_ (log *ε*) 208 (3.81), 236 (4.02), 253 (3.87), 271 (3.95), 291 (3.66), 340 (3.55) nm; IR (KBr) *ν*_max_ 3363, 2973, 2925, 1744, 1710, 1514, 1367, 1253, 1161, 1023, 901, 856, 579 cm^–1^; ^1^H and ^13^C NMR data, see Table 3; HRESIMS *m*/*z* 226.0507 [M − H]^−^ (calcd for C_13_H_8_NO_3_, 226.0504).

Euchrestifoline N (**14**): brown, non-crystalline solid; UV (MeOH) *λ*_max_ (log *ε*) 212 (4.07), 236 (4.21), 267 (3.83), 314 (3.76), 329 (3.75) nm; IR (KBr) *ν*_max_ 3401, 2965, 2917, 1700, 1622, 1469, 1294, 1207, 1140, 1007, 873, 830, 464 cm^–1^; ^1^H and ^13^C NMR data, see Table 3; HRESIMS *m*/*z* 280.1337 [M − H]^−^ (calcd for C_18_H_18_NO_2_, 280.1337).

Euchrestifoline O (**15**): brown, non-crystalline solid; UV (MeOH) *λ*_max_ (log *ε*) 212 (4.03), 236 (4.20), 258 (3.90), 309 (3.69), 330 (3.46) nm; IR (KBr) *ν*_max_ 3385, 2924, 2853, 1701, 1621, 1513, 1466, 1366, 1274, 1162, 1039, 1008, 579 cm^–1^; ^1^H and ^13^C NMR data, see Table 3; HRESIMS *m*/*z* 226.0507 [M − H]^−^ (calcd for C_13_H_8_NO_3_, 226.0504).

### ECD calculations

The stereochemistry of **1–5** was preliminarily determined based on their NOE correlations and relevant coupling constants. Subsequently, Sybyl-X 2.0 software was utilized for their stochastic conformational search within a 6 kcal/mol energy window with the MMFF94s force field. The geometry was optimized using DFT at the B3LYP/6-31G(d) computational level. TDDFT ECD calculations of **1–5** were performed at either B3LYP/6-31+G(d) or B3LYP/6-311+G(d) level with the PCM (methanol). The ECD spectra were synthesized by fitting all conformational results according to the Boltzmann-calculated contribution in SpecDis v1.51 software with 0.3 eV as the half-bandwidth [32]. The calculated ECD spectra of the relevant diastereomers and enantiomers of **1**–**5** were directly compared with their experimental ECD spectra. The calculation software is Gaussian 09 [33].

### Preparation of the (R)- and (S)-MPA esters of 8

Weighed 1.0 mg of compound **8** and completely dissolved in 0.5 mL of CDCl_3_. Subsequently, a series of reagents were introduced in sequence: 4-dimethylaminopyridine (0.5 mg), dicyclohexylcarbodiimide (2 mg), and (*R*)-(+)-*α*-methyl-*α*-(trifluoromethyl)-phenylacetyl (MPA) (1.0 mg), and stirred vigorously at room temperature for 16 h to ensure the reaction was complete. Following this, the reaction products were isolated using semipreparative HPLC, with a MeCN-H_2_O solvent ratio of 70:30 (*v*/*v*) at a flow rate of 1.0 mL/min, which allowed for the collection of the (*R*)-MPA ester (**8**r) at 12.5 min. Following a similar procedure, the reaction of compound **8** (1.0 mg) with (*S*)-MPA led to the acquisition of the (*S*)-MPA ester (**8**s), also under the identical HPLC conditions, with the retention time of 12.8 min.

### Anti-inflammatory activity assay

RAW 264.7 cells line was sourced from Peking Union Medical College (Beijing, China). The procedures for cell cultivation, experimental techniques, and the subsequent analysis and interpretation of data adhere to the methods previously detailed [34]. The positive control was dexamethasone.

### Cytotoxicity assay

The cytotoxicity assay was performed in HepG2 cells sourced from Peking Union Medical College (Beijing, P.R. China). The assessment of cytotoxicity was performed utilizing the MTT assay. The experimental manipulations and data analysis were carried out with the protocols reported in the literature [35], and taxol was adopted as a positive control.

### Anti-ferroptosis in PC12 cell

PC12 cells were inoculated into 96-well microplates at a concentration of 1 × 10^4^/well and treated with a concentration of 2 μM erastin to induce ferroptosis. The isolates were then added to the cells. After 24 h, the culture medium was removed, and 0.5 g/L MTT was added and incubated in an incubator for 4 h. After addition of DMSO, the optical density was recorded using a microplate spectrophotometer at 570 nm.

## Conclusions

In summary, the chemical study of *M. euchrestifolia* resulted in the identification of 15 novel carbazole alkaloids labelled as euchrestifolines A–O. In a series of activity screens, these compounds showed different biological activities. Especially, compounds **2**,** 3**, **5**, **6**,** 8**,** 9**, **11**, and **14** exhibited neuroprotective effects superior to that of the positive control ferrostatin-1 against erastin-induced ferroptosis in PC12 cells, with EC_50_ values below 1 μM. Moreover, compounds **4a**, **4b**,** 6**, and **11**–**14** showed inhibition of LPS-induced NO production in RAW 264.7 cells with IC_50_ values spanning from 12.7 to 19.7 μM. For cytotoxicity, the IC_50_ values of compounds **4a**, **5a**, **5b**,** 6**, and** 8** were below 4.0 μM in HepG2 cells. These results deepen our understanding of the chemical and bioactivity diversity of carbazole alkaloids from *Murraya* species, and their significant anti-ferroptosis effects suggest a promising future in neuroprotection.

## Supplementary Information

Supplementary Material 1.

## Data Availability

All data generated or analyzed during this study are included in this published article and its supplementary information files.
