# Supplementary material for: Euchrestifolines A–O, fifteen novel carbazole alkaloids with potent anti-ferroptotic activity from Murraya euchrestifolia
Source: Nat Prod Bioprospect. 2025 Jan 2;15(1):5. doi: 10.1007/s13659-024-00483-7 (PMC11695541; doi:10.1007/s13659-024-00483-7)
Supplement: Supplementary file 1 — Supplementary Material 1. [file 13659_2024_483_MOESM1_ESM.doc]

**Supporting Information**

**Euchrestifolines A–O, fifteen novel carbazole alkaloids with potent anti-ferroptotic activity from *Murraya euchrestifolia***

Yue-Mei Chen^#^, Nan-Kai Cao^#^, Si-Si Zhu, Meng Ding, Hai-Zhen Liang, Ke-Wu Zeng, Ming-Bo Zhao, Peng-Fei Tu, Yong Jiang^*^

State Key Laboratory of Natural and Biomimetic Drugs, School of Pharmaceutical Sciences, Peking University, Beijing 100191, People’s Republic of China

*Corresponding author.

1. mail: [yongjiang@bjmu.edu.cn](mailto:yongjiang@bjmu.edu.cn). (Y. Jiang).

**List of Contents**

**Table S1.** Conformational analysis of (3ʹ*S*, 5ʹʹ*S*)-**1**

**Table S2.** Conformational analysis of (3ʹ*S*, 5ʹʹ*R*)-**1**

**Table S3.** Conformational analysis of (3ʹ*S*, 5ʹʹ*S*)-**2**

**Table S4.** Conformational analysis of (3ʹ*S*, 5ʹʹ*R*)-**2**

**Table S5.** Conformational analysis of (5ʹʹ*R*)-**3**

**Table S6** Coordinates of (3ʹ*S*, 5ʹʹ*R*)-**1**

**Table S7** Coordinates of (3ʹ*S*, 5ʹʹ*S*)-**1**

**Table S8** Coordinates of (3ʹ*S*, 5ʹʹ*R*)-**2**

**Table S9** Coordinates of (3ʹ*S*, 5ʹʹ*S*)-**2**

**Table S10** Coordinates of (5ʹʹ*R*)-**3**

The NMR data for (*R*)-MPA ester (**8r**).

The NMR data for (*S*)-MPA ester (**8s**).

Determinations of ECD data of the *in situ* formed transition metal complexes of **4** and **9**.

**Figure S1**. The key HMBC correlations of compounds **8**–**15**.

**Figure S2**. The chiral HPLC separation and experimental and calculated ECD data of compounds **4a** and **4b**.

**Figure S3.** The chiral HPLC separation and experimental and calculated ECD data of compounds **5a** and **5b**.

**Figure S4.** The chiral HPLC analysis of compound **6**

**Figure S5.** HRESIMS spectrum for **1**.

**Figure S6.** UV spectrum for **1**.

**Figure S7.** IR spectrum for **1**.

**Figure S8.** ^1^H NMR spectrum for **1** in acetone-*d*_6_.

**Figure S9.** ^13^C NMR spectrum for **1** in acetone-*d*_6_.

**Figure S10.** ^1^H-^1^H gCOSY spectrum for **1** in acetone-*d*_6_.

**Figure S11.** HSQC spectrum for **1** in acetone-*d*_6_.

**Figure S12.** HMBC spectrum for **1** in acetone-*d*_6_.

**Figure S13**. ECD spectrum of **1** and its precursor of mahanine in MeOH.

**Figure S14.** HRESIMS spectrum for **2**.

**Figure S15.** UV spectrum for **2**.

**Figure S16.** IR spectrum for **2**.

**Figure S17.** ^1^H NMR spectrum for **2** in acetone-*d*_6_.

**Figure S18.** ^13^C NMR spectrum for **2** in acetone-*d*_6_.

**Figure S19.** ^1^H-^1^H gCOSY spectrum for **2** in acetone-*d*_6_

**Figure S20.** HSQC spectrum for **2** in acetone-*d*_6_.

**Figure S21.** HMBC spectrum for **2** in acetone-*d*_6_.

**Figure S22.** ECD spectrum for **2** in MeOH.

**Figure S23.** HRESIMS spectrum for **3**.

**Figure S24.** UV spectrum for **3**.

**Figure S25.** IR spectrum for **3**.

**Figure S26.** ^1^H NMR spectrum for **3** in acetone-*d*_6_.

**Figure S27.** ^13^C NMR spectrum for **3** in acetone-*d*_6_.

**Figure S28.** ^1^H-^1^H gCOSY spectrum for **3** in acetone-*d*_6_.

**Figure S29.** HSQC spectrum for **3** in acetone-*d*_6_.

**Figure S30.** HMBC spectrum for **3** in acetone-*d*_6_.

**Figure S31.** ECD spectrum for **3** in MeOH.

**Figure S32.** HRESIMS spectrum for **4**.

**Figure S33.** UV spectrum for **4**.

**Figure S34.** IR spectrum for **4**.

**Figure S35.** ^1^H NMR spectrum for **4** in CDCl_3_.

**Figure S36.** ^13^C NMR spectrum for **4** in CDCl_3_.

**Figure S37.** ^1^H-^1^H gCOSY spectrum for **4** in CDCl_3_.

**Figure S38.** HSQC spectrum for **4** in CDCl_3_.

**Figure S39.** HMBC spectrum for **4** in CDCl_3_.

**Figure S40.** ECD spectra for **4a** and **4b** in MeOH.

**Figure S41.** HRESIMS spectrum for **5**.

**Figure S42.** UV spectrum for **5**.

**Figure S43.** IR spectrum for **5**.

**Figure S44.** ^1^H NMR spectrum for **5** in acetone-*d*_6_.

**Figure S45.** ^13^C NMR spectrum for **5** in acetone-*d*_6_.

**Figure S46.** HSQC spectrum for **5** in acetone-*d*_6_.

**Figure S47.** HMBC spectrum for **5** in acetone-*d*_6_.

**Figure S48.** ECD spectra for **5a** and **5b** in MeOH.

**Figure S49.** HRESIMS spectrum for **6**.

**Figure S50.** UV spectrum for **6**.

**Figure S51.** IR spectrum for **6**.

**Figure S52.** ^1^H NMR spectrum for **6** in acetone-*d*_6_.

**Figure S53.** ^13^C NMR spectrum for **6** in acetone-*d*_6_.

**Figure S54.** HSQC spectrum for **6** in acetone-*d*_6_.

**Figure S55.** HMBC spectrum for **6** in acetone-*d*_6_.

**Figure S56.** ECD spectrum for **6** in MeOH.

**Figure S57.** HRESIMS spectrum for **7**.

**Figure S58.** UV spectrum for **7**.

**Figure S59.** IR spectrum for **7**.

**Figure S60.** ^1^H NMR spectrum for **7** in CDCl_3_.

**Figure S61.** ^13^C NMR spectrum for **7** in CDCl_3_.

**Figure S62.** ^1^H-^1^H gCOSY spectrum for **7** in CDCl_3_

**Figure S63.** HSQC spectrum for **7** in CDCl_3_.

**Figure S64.** HMBC spectrum for **7** in CDCl_3_.

**Figure S65.** HRESIMS spectrum for **8**.

**Figure S66.** UV spectrum for **8**.s

**Figure S67.** IR spectrum for **8**.

**Figure S68.** ^1^H NMR spectrum for **8** in CDCl_3_.

**Figure S69.** ^13^C NMR spectrum for **8** in CDCl_3_.

**Figure S70.** HSQC spectrum for **8** in CDCl_3_.

**Figure S71.** HMBC spectrum for **8** in CDCl_3_.

**Figure S72.** ECD spectrum for **8** in MeOH.

**Figure S73.** HRESIMS spectrum for **9**.

**Figure S74.** UV spectrum for **9**.

**Figure S75.** IR spectrum for **9**.

**Figure S76.** ^1^H NMR spectrum for **9** in acetone-*d*_6_.

**Figure S77.** ^13^C NMR spectrum for **9** in acetone-*d*_6_.

**Figure S78.** HSQC spectrum for **9** in acetone-*d*_6_.

**Figure S79.** HMBC spectrum for **9** in acetone-*d*_6_.

**Figure S80.** ECD spectrum for **9** in MeOH.

**Figure S81.** HRESIMS spectrum for **10**.

**Figure S82.** UV spectrum for **10**.

**Figure S83.** IR spectrum for **10**.

**Figure S84.** ^1^H NMR spectrum for **10** in CDCl_3_.

**Figure S85.** ^13^C NMR spectrum for **10** in CDCl_3_.

**Figure S86.** HSQC spectrum for **10** in CDCl_3_.

**Figure S87** HMBC spectrum for **10** in CDCl_3_.

**Figure S88.** HRESIMS spectrum for **11**.

**Figure S89.** UV spectrum for **11**.

**Figure S90.** IR spectrum for **11**.

**Figure S91.** ^1^H NMR spectrum for **11** in CDCl_3_.

**Figure S92.** ^13^C NMR spectrum for **11** in CDCl_3_.

**Figure S93.** HSQC spectrum for **11** in CDCl_3_.

**Figure S94.** HMBC spectrum for **11** in CDCl_3_.

**Figure S95.** ECD spectrum for **11** in MeOH.

**Figure S96.** HRESIMS spectrum for **12**.

**Figure S97.** UV spectrum for **12**.

**Figure S98.** IR spectrum for **12**.

**Figure S99.** ^1^H NMR spectrum for **12** in CDCl_3_.

**Figure S100.** ^13^C NMR spectrum for **12** in CDCl_3_.

**Figure S101.** HSQC spectrum for **12** in CDCl_3_.

**Figure S102.** HMBC spectrum for **12** in CDCl_3_.

**Figure S103.** HRESIMS spectrum for **13**.

**Figure S104.** UV spectrum for **13**.

**Figure S105.** IR spectrum for **13**.

**Figure S106.** ^1^H NMR spectrum for **13** in acetone-*d*_6_.

**Figure S107.** ^13^C NMR spectrum for **13** in acetone-*d*_6_.

**Figure S108.** HSQC spectrum for **13** in acetone-*d*_6_.

**Figure S109.** HMBC spectrum for **13** in acetone-*d*_6_.

**Figure S110.** HRESIMS spectrum for **14**.

**Figure S111.** UV spectrum for **14**.

**Figure S112.** IR spectrum for **14**.

**Figure S113.** ^1^H NMR spectrum for **14** in acetone-*d*_6_.

**Figure S114.** ^13^C NMR spectrum for **14** in acetone-*d*_6_.

**Figure S115.** HSQC spectrum for **14** in acetone-*d*_6_.

**Figure S116.** HMBC spectrum for **14** in acetone-*d*_6_.

**Figure S117.** HRESIMS spectrum for **15**.

**Figure S118.** UV spectrum for **15**.

**Figure S119.** IR spectrum for **15**.

**Figure S120.** ^1^H NMR spectrum for **15** in acetone-*d*_6_.

**Figure S121.** ^13^C NMR spectrum for **15** in acetone-*d*_6_.

**Figure S122.** HSQC spectrum for **15** in acetone-*d*_6_.

**Figure S123.** HMBC spectrum for **15** in acetone-*d*_6_.

**Table S1.** Conformational analysis of (3ʹ*S*, 5ʹʹ*S*)-**1**

| Conformers of (3ʹ*S*, 5ʹʹ*S*)-**1** | Gibbs Free Energy (Hartree) | Relative Gibbs Free Energy (kcal/mol) | Population (%) |
| --- | --- | --- | --- |
| 7 | -1382.4647187 | 0 | 32.96 |
| 5 | -1382.4644251 | 0.000294 | 24.14 |
| 12 | -1382.4643665 | 0.000352 | 22.69 |
| 11 | -1382.4642577 | 0.000461 | 20.22 |

**Table S2. Conformational analysis of (3ʹ*S*, 5ʹʹ*R*)-1**

| Conformers of (3ʹ*S*, 5ʹʹ*R*)-**1** | Gibbs Free Energy (Hartree) | Relative Gibbs Free Energy (kcal/mol) | Population (%) |
| --- | --- | --- | --- |
| 2 | -1382.4659805 | 0 | 44.22 |
| 1 | -1382.4659357 | 4.48E-05 | 46.37 |
| 6 | -1382.4634789 | 0.002502 | 3.27 |
| 4 | -1382.4634435 | 0.002537 | 3.15 |
| 3 | -1382.463397 | 0.002584 | 3.00 |

**Table S3.** Conformational analysis of (3ʹ*S*, 5ʹʹ*S*)-**2**

| Conformers of (3ʹ*S*, 5ʹʹ*S*)-**2** | Gibbs Free Energy (Hartree) | Relative Gibbs Free Energy (kcal/mol) | Population (%) |
| --- | --- | --- | --- |
| 13 | -1382.5509047 | 0 | 36.94 |
| 43 | -1382.5507804 | 0.000124 | 32.38 |
| 23 | -1382.5496553 | 0.001249 | 9.82 |
| 58 | -1382.5493962 | 0.001509 | 7.46 |
| 34 | -1382.5493645 | 0.00154 | 7.22 |
| 75 | -1382.5492161 | 0.001689 | 6.17 |

**Table S4.** Conformational analysis of (3ʹ*S*, 5ʹʹ*R*)-**2**

| Conformers of (3ʹ*S*, 5ʹʹ*R*)-**1** | Gibbs Free Energy (Hartree) | Relative Gibbs Free Energy (kcal/mol) | Population (%) |
| --- | --- | --- | --- |
| 13 | -1382.5508887 | 0 | 35.51 |
| 41 | -1382.5508042 | 0.0000845 | 32.47 |
| 23 | -1382.5496701 | 0.0012186 | 9.76 |
| 33 | -1382.5494225 | 0.0014662 | 7.50 |
| 58 | -1382.5494064 | 0.0014823 | 7.38 |
| 22 | -1382.5494063 | 0.001482 | 7.38 |

**Table S5. Conformational analysis of (5ʹʹ*R*)-3**

| Conformers of (5ʹʹ*R*)-**3** | Gibbs Free Energy (Hartree) | Relative Gibbs Free Energy (kcal/mol) | Population (%) |
| --- | --- | --- | --- |
| 1 | -1423.0339472 | 0 | 29.64 |
| 28 | -1423.0338939 | 0.0000533 | 28.01 |
| 23 | -1423.033172 | 0.0007752 | 13.03 |
| 30 | -1423.0330896 | 0.0008576 | 11.94 |
| 25 | -1423.0329928 | 0.0009544 | 10.77 |
| 16 | -1423.0325322 | 0.001415 | 6.61 |

**Table S6 Coordinates of (3ʹ*S*, 5ʹʹ*R*)-1**

|  | 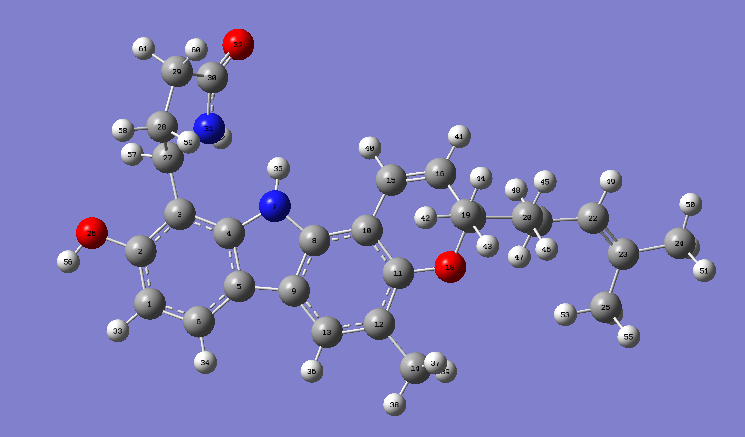  Conformer (3ʹ*S*, 5ʹʹ*R*)-**1** (2) | **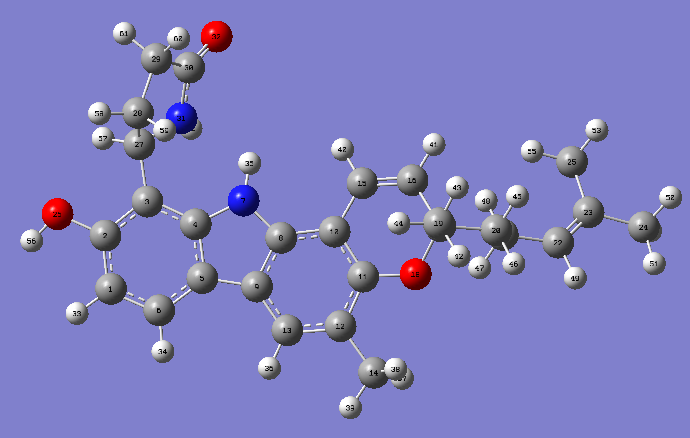**  Conformer (3ʹ*S*, 5ʹʹ*R*)-**1** (1) | **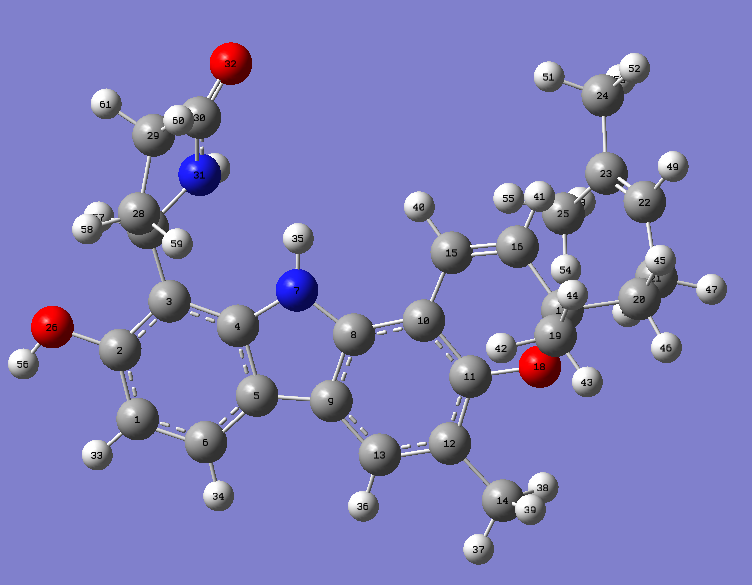**  **Conformer (3ʹ*S*, 5ʹʹ*R*)-1 (6)** |
| --- | --- | --- | --- |
| | 1 | | --- | | 2 | | 3 | | 4 | | 5 | | 6 | | 7 | | 8 | | 9 | | 10 | | 11 | | 12 | | 13 | | 14 | | 15 | | 16 | | 17 | | 18 | | 19 | | 20 | | 21 | | 22 | | 23 | | 24 | | 25 | | 26 | | 27 | | 28 | | 29 | | 30 | | 31 | | 32 | | 33 | | 34 | | 35 | | 36 | | 37 | | 38 | | 39 | | 40 | | 41 | | 42 | | 43 | | 44 | | 45 | | 46 | | 47 | | 48 | | 49 | | 50 | | 51 | | 52 | | 53 | | 54 | | 55 | | 56 | | 57 | | 58 | | 59 | | 60 | | 61 | | 62 | | | -4.71034 | -2.79834 | -0.7031 | | --- | --- | --- | | -5.17419 | -1.47401 | -0.71954 | | -4.32929 | -0.38339 | -0.48207 | | -2.98605 | -0.69525 | -0.22409 | | -2.49166 | -2.02606 | -0.20241 | | -3.37418 | -3.07813 | -0.44448 | | -1.93366 | 0.162988 | 0.053847 | | -0.76698 | -0.57157 | 0.222734 | | -1.07327 | -1.94238 | 0.084894 | | 0.531951 | -0.12134 | 0.492074 | | 1.520096 | -1.10824 | 0.641065 | | 1.255592 | -2.48682 | 0.501254 | | -0.05016 | -2.88544 | 0.223894 | | 2.375105 | -3.48578 | 0.644875 | | 0.937184 | 1.274168 | 0.574596 | | 2.163617 | 1.59683 | 0.999964 | | 3.124999 | 0.534471 | 1.482034 | | 2.822026 | -0.75171 | 0.858134 | | 3.008987 | 0.365765 | 3.005109 | | 4.577926 | 0.839696 | 1.085123 | | 4.824386 | 0.984275 | -0.42625 | | 6.248783 | 1.359613 | -0.73079 | | 7.176675 | 0.656164 | -1.3932 | | 8.566884 | 1.207457 | -1.59684 | | 6.957806 | -0.70988 | -1.99305 | | -6.50188 | -1.1922 | -0.95712 | | -4.88263 | 1.021839 | -0.46402 | | -5.15945 | 1.595027 | 0.953472 | | -5.0175 | 3.113146 | 0.777794 | | -4.09622 | 3.277946 | -0.42962 | | -3.95723 | 2.033062 | -1.00533 | | -3.57806 | 4.300781 | -0.82952 | | -5.4091 | -3.60978 | -0.89059 | | -3.03082 | -4.1075 | -0.43381 | | -2.0022 | 1.163467 | -0.03216 | | -0.26627 | -3.9449 | 0.11912 | | 2.870284 | -3.40123 | 1.616521 | | 1.998918 | -4.50606 | 0.543318 | | 3.150623 | -3.33582 | -0.11241 | | 0.245245 | 2.052459 | 0.26493 | | 2.488387 | 2.631214 | 1.056714 | | 1.994621 | 0.070273 | 3.28274 | | 3.706036 | -0.39844 | 3.359436 | | 3.236948 | 1.306457 | 3.514179 | | 4.8784 | 1.759557 | 1.601393 | | 5.21486 | 0.042766 | 1.482045 | | 4.533223 | 0.058421 | -0.92305 | | 4.15865 | 1.761801 | -0.82319 | | 6.549131 | 2.335649 | -0.34584 | | 8.685383 | 2.196926 | -1.1499 | | 9.323963 | 0.545776 | -1.15875 | | 8.808305 | 1.288479 | -2.66368 | | 5.956727 | -1.10477 | -1.82232 | | 7.128533 | -0.68978 | -3.07616 | | 7.674929 | -1.43099 | -1.58301 | | -6.97856 | -2.00598 | -1.15627 | | -5.81233 | 1.030822 | -1.04033 | | -6.13695 | 1.279816 | 1.319407 | | -4.40681 | 1.217201 | 1.65035 | | -4.61408 | 3.637265 | 1.645277 | | -5.97039 | 3.592399 | 0.527136 | | -3.6246 | 1.978592 | -1.95924 | | | -4.99158 | -2.36024 | -1.01332 | | --- | --- | --- | | -5.29818 | -1.00167 | -0.84164 | | -4.33053 | -0.05745 | -0.47852 | | -3.03119 | -0.55322 | -0.29428 | | -2.69498 | -1.92238 | -0.46238 | | -3.69547 | -2.82374 | -0.82399 | | -1.88449 | 0.133011 | 0.073823 | | -0.81017 | -0.74644 | 0.116784 | | -1.27476 | -2.04156 | -0.19732 | | 0.533836 | -0.48884 | 0.416235 | | 1.401123 | -1.59349 | 0.41008 | | 0.977252 | -2.90075 | 0.091678 | | -0.36757 | -3.10568 | -0.20862 | | 1.974095 | -4.03116 | 0.0748 | | 1.099263 | 0.826732 | 0.677174 | | 2.357402 | 0.946052 | 1.115179 | | 3.19255 | -0.27479 | 1.427392 | | 2.736752 | -1.42177 | 0.645836 | | 3.071253 | -0.63326 | 2.916849 | | 4.667645 | -0.08578 | 1.039355 | | 4.916185 | 0.243409 | -0.44252 | | 6.384105 | 0.313616 | -0.76271 | | 7.099268 | 1.356884 | -1.2039 | | 8.579221 | 1.223738 | -1.46937 | | 6.541974 | 2.729849 | -1.48436 | | -6.58554 | -0.53997 | -1.01013 | | -4.71626 | 1.386332 | -0.25959 | | -4.91508 | 1.78922 | 1.22796 | | -4.59673 | 3.29043 | 1.255358 | | -3.67067 | 3.51102 | 0.060585 | | -3.68269 | 2.348875 | -0.68067 | | -3.03862 | 4.512224 | -0.20946 | | -5.78121 | -3.05329 | -1.29269 | | -3.47421 | -3.87749 | -0.95916 | | -1.83636 | 1.137185 | 0.122761 | | -0.70605 | -4.10918 | -0.45051 | | 2.758848 | -3.86692 | -0.66989 | | 2.479245 | -4.13908 | 1.03903 | | 1.481932 | -4.97789 | -0.1582 | | 0.500455 | 1.713989 | 0.490994 | | 2.800469 | 1.91891 | 1.304637 | | 3.676895 | -1.51351 | 3.148961 | | 3.412626 | 0.19738 | 3.540943 | | 2.031705 | -0.84496 | 3.177399 | | 5.084691 | 0.708783 | 1.668594 | | 5.204055 | -1.00283 | 1.306392 | | 4.452168 | -0.54232 | -1.04907 | | 4.403344 | 1.171035 | -0.70674 | | 6.921261 | -0.62257 | -0.6026 | | 9.156768 | 1.929887 | -0.86043 | | 8.945091 | 0.217184 | -1.25541 | | 8.816246 | 1.453063 | -2.51545 | | 7.06328 | 3.48859 | -0.88846 | | 6.698132 | 3.004636 | -2.53444 | | 5.475948 | 2.818552 | -1.27594 | | -7.15528 | -1.25945 | -1.30511 | | -5.64223 | 1.58017 | -0.80874 | | -5.92066 | 1.542321 | 1.569517 | | -4.20793 | 1.235349 | 1.850863 | | -4.12833 | 3.640787 | 2.176161 | | -5.48821 | 3.906541 | 1.093632 | | -3.36516 | 2.386981 | -1.6405 | | | -4.52441 | -2.48373 | -1.04273 | | --- | --- | --- | | -4.86208 | -1.15287 | -0.75284 | | -3.91174 | -0.21619 | -0.3296 | | -2.59657 | -0.69007 | -0.21067 | | -2.22888 | -2.03105 | -0.49862 | | -3.21328 | -2.92596 | -0.9162 | | -1.4608 | -0.006 | 0.192564 | | -0.36343 | -0.85675 | 0.142929 | | -0.80089 | -2.13331 | -0.27061 | | 0.97882 | -0.58738 | 0.438862 | | 1.873227 | -1.66358 | 0.323534 | | 1.477824 | -2.95081 | -0.09641 | | 0.132836 | -3.16773 | -0.38812 | | 2.503268 | -4.04807 | -0.22537 | | 1.515952 | 0.716897 | 0.799366 | | 2.77953 | 0.834289 | 1.222889 | | 3.640479 | -0.39046 | 1.430766 | | 3.209131 | -1.47244 | 0.548728 | | 3.509537 | -0.87575 | 2.884722 | | 5.124381 | -0.17181 | 1.086066 | | 5.447208 | 0.234595 | -0.36546 | | 5.225927 | 1.690381 | -0.67752 | | 4.488453 | 2.240521 | -1.65066 | | 4.417303 | 3.738945 | -1.81361 | | 3.662682 | 1.468007 | -2.6461 | | -6.164 | -0.71406 | -0.85929 | | -4.32874 | 1.194013 | 0.014631 | | -4.49916 | 1.472059 | 1.533888 | | -4.21887 | 2.974768 | 1.673456 | | -3.32986 | 3.314723 | 0.478449 | | -3.33145 | 2.215071 | -0.35235 | | -2.73079 | 4.351226 | 0.274219 | | -5.30162 | -3.17243 | -1.36449 | | -2.96806 | -3.95851 | -1.14272 | | -1.4364 | 0.991343 | 0.325435 | | -0.18442 | -4.15654 | -0.70705 | | 2.031664 | -4.98471 | -0.5305 | | 3.270332 | -3.80039 | -0.96542 | | 3.028216 | -4.22374 | 0.718113 | | 0.891933 | 1.600232 | 0.69602 | | 3.202694 | 1.798902 | 1.478295 | | 2.469805 | -1.11685 | 3.116602 | | 4.121049 | -1.76729 | 3.048478 | | 3.836895 | -0.09721 | 3.57951 | | 5.526225 | 0.573871 | 1.782542 | | 5.641671 | -1.11001 | 1.31113 | | 6.51043 | 0.011454 | -0.52858 | | 4.898633 | -0.41764 | -1.04603 | | 5.770504 | 2.37376 | -0.02269 | | 4.754113 | 4.046168 | -2.81107 | | 3.386094 | 4.099139 | -1.71284 | | 5.030718 | 4.262273 | -1.07674 | | 3.983438 | 1.689684 | -3.6711 | | 3.70955 | 0.388702 | -2.50761 | | 2.608841 | 1.764033 | -2.58185 | | -6.72053 | -1.4218 | -1.20354 | | -5.27329 | 1.405449 | -0.4949 | | -5.48887 | 1.171246 | 1.878519 | | -3.7622 | 0.89045 | 2.093753 | | -3.73617 | 3.264083 | 2.607954 | | -5.12988 | 3.576651 | 1.582404 | | -3.03743 | 2.336379 | -1.3127 | |

|  | 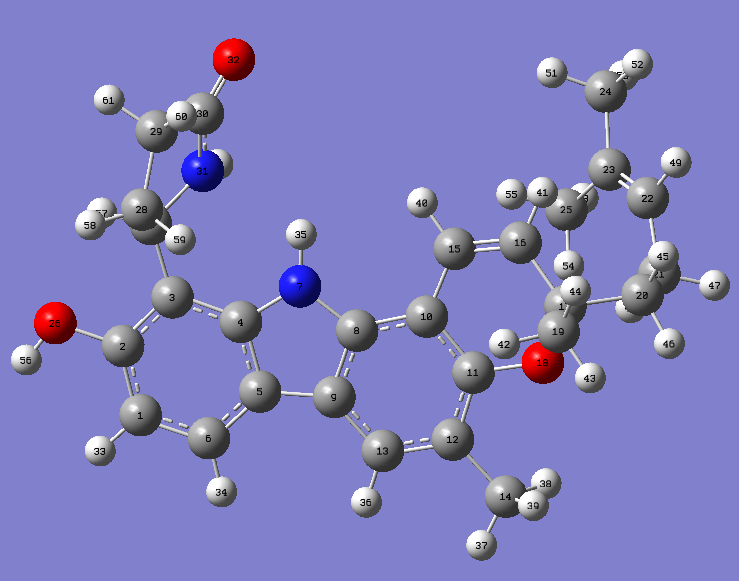  Conformer (3ʹ*S*, 5ʹʹ*R*)-**1** (6) | 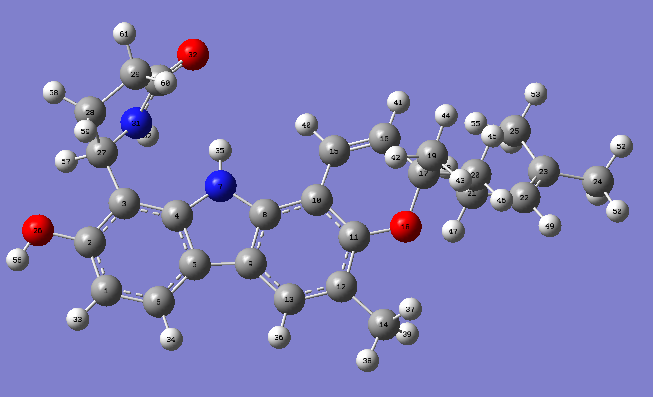  **Conformer (3ʹ*S*, 5ʹʹ*R*)-1 (3)** |
| --- | --- | --- |
| | 1 | | --- | | 2 | | 3 | | 4 | | 5 | | 6 | | 7 | | 8 | | 9 | | 10 | | 11 | | 12 | | 13 | | 14 | | 15 | | 16 | | 17 | | 18 | | 19 | | 20 | | 21 | | 22 | | 23 | | 24 | | 25 | | 26 | | 27 | | 28 | | 29 | | 30 | | 31 | | 32 | | 33 | | 34 | | 35 | | 36 | | 37 | | 38 | | 39 | | 40 | | 41 | | 42 | | 43 | | 44 | | 45 | | 46 | | 47 | | 48 | | 49 | | 50 | | 51 | | 52 | | 53 | | 54 | | 55 | | 56 | | 57 | | 58 | | 59 | | 60 | | 61 | | 62 | | | 5.065335 | 2.232806 | -0.97457 | | --- | --- | --- | | 5.340516 | 0.860869 | -0.90741 | | 4.360813 | -0.09652 | -0.61119 | | 3.068562 | 0.402964 | -0.37594 | | 2.766695 | 1.79339 | -0.44 | | 3.778907 | 2.701737 | -0.74211 | | 1.893927 | -0.26751 | -0.0541 | | 0.853721 | 0.641765 | 0.079106 | | 1.35469 | 1.93849 | -0.15129 | | -0.49225 | 0.403238 | 0.38631 | | -1.32445 | 1.531208 | 0.467015 | | -0.86468 | 2.843927 | 0.228568 | | 0.481567 | 3.028642 | -0.07751 | | -1.82667 | 4.001923 | 0.30231 | | -1.09068 | -0.91041 | 0.576198 | | -2.34616 | -1.01932 | 1.024249 | | -3.14456 | 0.201541 | 1.420052 | | -2.66106 | 1.385449 | 0.713213 | | -3.00621 | 0.458271 | 2.928961 | | -4.62644 | 0.079824 | 1.030399 | | -4.89036 | -0.14722 | -0.46791 | | -6.36078 | -0.15418 | -0.78406 | | -7.10941 | -1.14894 | -1.2786 | | -8.58554 | -0.95625 | -1.52839 | | -6.59554 | -2.52073 | -1.63705 | | 6.615442 | 0.381411 | -1.12373 | | 4.78369 | -1.55811 | -0.5575 | | 5.333027 | -2.02828 | 0.827518 | | 4.167719 | -2.782 | 1.487414 | | 3.244465 | -3.16498 | 0.333991 | | 3.691012 | -2.5038 | -0.775 | | 2.25343 | -3.87401 | 0.381139 | | 5.866856 | 2.92898 | -1.20867 | | 3.572521 | 3.765686 | -0.79592 | | 1.79142 | -1.26431 | 0.020923 | | 0.847762 | 4.035677 | -0.25612 | | -2.31492 | 4.06024 | 1.279432 | | -1.30909 | 4.94712 | 0.124603 | | -2.62635 | 3.910864 | -0.43903 | | -0.51792 | -1.80225 | 0.337614 | | -2.8122 | -1.98952 | 1.166448 | | -1.95992 | 0.624396 | 3.195447 | | -3.58655 | 1.3373 | 3.222398 | | -3.36646 | -0.40219 | 3.499716 | | -5.06332 | -0.74124 | 1.610148 | | -5.13517 | 0.992859 | 1.358481 | | -4.40573 | 0.661576 | -1.02609 | | -4.40569 | -1.07064 | -0.79284 | | -6.86837 | 0.787499 | -0.56904 | | -8.91945 | 0.04763 | -1.25724 | | -8.83351 | -1.11998 | -2.58423 | | -9.18231 | -1.677 | -0.95617 | | -7.13786 | -3.29491 | -1.08109 | | -6.76359 | -2.73246 | -2.69984 | | -5.53212 | -2.65322 | -1.43888 | | 7.205881 | 1.109496 | -1.34826 | | 5.550466 | -1.7066 | -1.3185 | | 6.176184 | -2.70282 | 0.661469 | | 5.695302 | -1.18617 | 1.415592 | | 3.597165 | -2.15789 | 2.182593 | | 4.468441 | -3.67548 | 2.036587 | | 3.195461 | -2.5783 | -1.65217 | | | 5.065335 | 2.232806 | -0.97457 | | --- | --- | --- | | 5.340516 | 0.860869 | -0.90741 | | 4.360813 | -0.09652 | -0.61119 | | 3.068562 | 0.402964 | -0.37594 | | 2.766695 | 1.79339 | -0.44 | | 3.778907 | 2.701737 | -0.74211 | | 1.893927 | -0.26751 | -0.0541 | | 0.853721 | 0.641765 | 0.079106 | | 1.35469 | 1.93849 | -0.15129 | | -0.49225 | 0.403238 | 0.38631 | | -1.32445 | 1.531208 | 0.467015 | | -0.86468 | 2.843927 | 0.228568 | | 0.481567 | 3.028642 | -0.07751 | | -1.82667 | 4.001923 | 0.30231 | | -1.09068 | -0.91041 | 0.576198 | | -2.34616 | -1.01932 | 1.024249 | | -3.14456 | 0.201541 | 1.420052 | | -2.66106 | 1.385449 | 0.713213 | | -3.00621 | 0.458271 | 2.928961 | | -4.62644 | 0.079824 | 1.030399 | | -4.89036 | -0.14722 | -0.46791 | | -6.36078 | -0.15418 | -0.78406 | | -7.10941 | -1.14894 | -1.2786 | | -8.58554 | -0.95625 | -1.52839 | | -6.59554 | -2.52073 | -1.63705 | | 6.615442 | 0.381411 | -1.12373 | | 4.78369 | -1.55811 | -0.5575 | | 5.333027 | -2.02828 | 0.827518 | | 4.167719 | -2.782 | 1.487414 | | 3.244465 | -3.16498 | 0.333991 | | 3.691012 | -2.5038 | -0.775 | | 2.25343 | -3.87401 | 0.381139 | | 5.866856 | 2.92898 | -1.20867 | | 3.572521 | 3.765686 | -0.79592 | | 1.79142 | -1.26431 | 0.020923 | | 0.847762 | 4.035677 | -0.25612 | | -2.31492 | 4.06024 | 1.279432 | | -1.30909 | 4.94712 | 0.124603 | | -2.62635 | 3.910864 | -0.43903 | | -0.51792 | -1.80225 | 0.337614 | | -2.8122 | -1.98952 | 1.166448 | | -1.95992 | 0.624396 | 3.195447 | | -3.58655 | 1.3373 | 3.222398 | | -3.36646 | -0.40219 | 3.499716 | | -5.06332 | -0.74124 | 1.610148 | | -5.13517 | 0.992859 | 1.358481 | | -4.40573 | 0.661576 | -1.02609 | | -4.40569 | -1.07064 | -0.79284 | | -6.86837 | 0.787499 | -0.56904 | | -8.91945 | 0.04763 | -1.25724 | | -8.83351 | -1.11998 | -2.58423 | | -9.18231 | -1.677 | -0.95617 | | -7.13786 | -3.29491 | -1.08109 | | -6.76359 | -2.73246 | -2.69984 | | -5.53212 | -2.65322 | -1.43888 | | 7.205881 | 1.109496 | -1.34826 | | 5.550466 | -1.7066 | -1.3185 | | 6.176184 | -2.70282 | 0.661469 | | 5.695302 | -1.18617 | 1.415592 | | 3.597165 | -2.15789 | 2.182593 | | 4.468441 | -3.67548 | 2.036587 | | 3.195461 | -2.5783 | -1.65217 | |

**Table S7 Coordinates of (3ʹ*S*, 5ʹʹ*S*)-1**

|  | 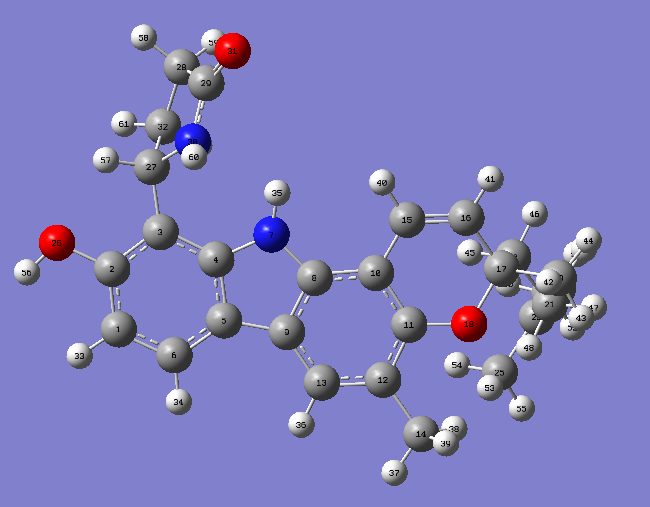  Conformer (3ʹ*S*, 5ʹʹ*S*)-**1** (5) | 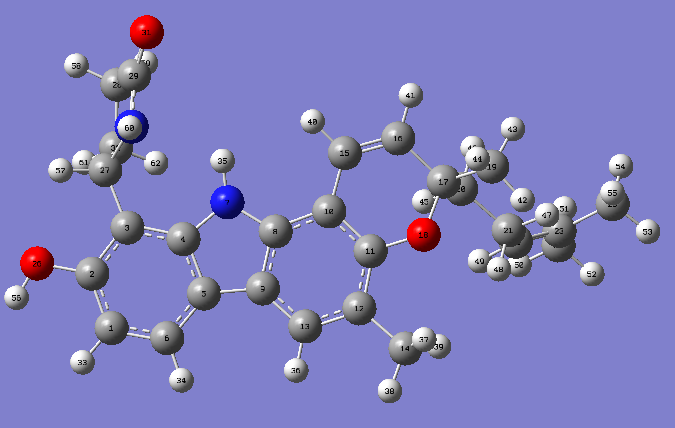  Conformer (3ʹ*S*, 5ʹʹ*S*)-**1** (7) | 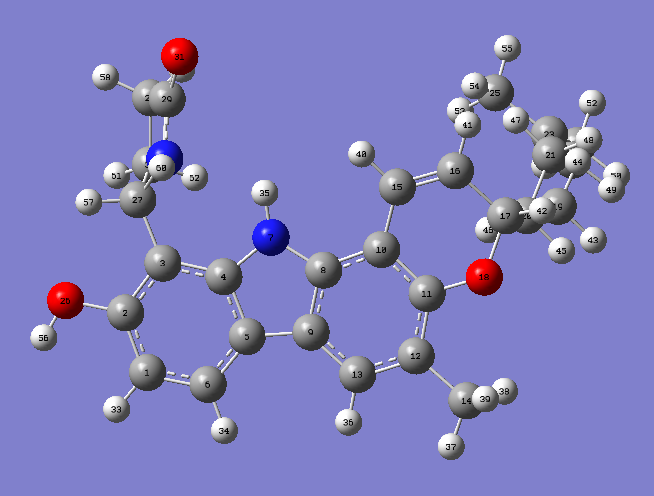  **Conformer (3ʹ*S*, 5ʹʹ*S*)-1 (11)** |
| --- | --- | --- | --- |
| | 1 | | --- | | 2 | | 3 | | 4 | | 5 | | 6 | | 7 | | 8 | | 9 | | 10 | | 11 | | 12 | | 13 | | 14 | | 15 | | 16 | | 17 | | 18 | | 19 | | 20 | | 21 | | 22 | | 23 | | 24 | | 25 | | 26 | | 27 | | 28 | | 29 | | 30 | | 31 | | 32 | | 33 | | 34 | | 35 | | 36 | | 37 | | 38 | | 39 | | 40 | | 41 | | 42 | | 43 | | 44 | | 45 | | 46 | | 47 | | 48 | | 49 | | 50 | | 51 | | 52 | | 53 | | 54 | | 55 | | 56 | | 57 | | 58 | | 59 | | 60 | | 61 | | 62 | | | 4.003419 | -3.19395 | 0.139774 | | --- | --- | --- | | 4.646439 | -1.97374 | 0.398286 | | 3.992922 | -0.74255 | 0.268659 | | 2.650763 | -0.80223 | -0.13528 | | 1.977271 | -2.02336 | -0.40261 | | 2.672518 | -3.22337 | -0.25822 | | 1.757447 | 0.238401 | -0.33566 | | 0.535177 | -0.27177 | -0.75337 | | 0.625232 | -1.67961 | -0.79653 | | -0.63968 | 0.410739 | -1.09354 | | -1.74328 | -0.3791 | -1.455 | | -1.68692 | -1.78686 | -1.52888 | | -0.49248 | -2.42046 | -1.19265 | | -2.89351 | -2.56639 | -1.98516 | | -0.77956 | 1.857538 | -1.16839 | | -1.9851 | 2.41076 | -1.33983 | | -3.23405 | 1.559091 | -1.37234 | | -2.91539 | 0.210793 | -1.84014 | | -4.2323 | 2.094092 | -2.3989 | | -3.82507 | 1.476391 | 0.055705 | | -5.12554 | 0.665387 | 0.210206 | | -5.60002 | 0.646488 | 1.638395 | | -5.68324 | -0.39151 | 2.480922 | | -6.18351 | -0.20349 | 3.89247 | | -5.30574 | -1.81117 | 2.14148 | | 5.960482 | -1.94003 | 0.811605 | | 4.712228 | 0.542977 | 0.602197 | | 4.623466 | 2.627947 | 1.859042 | | 4.406464 | 2.901571 | 0.371829 | | 4.332813 | 1.677665 | -0.25814 | | 4.318626 | 3.981981 | -0.17558 | | 4.40128 | 1.116965 | 2.012327 | | 4.555744 | -4.12274 | 0.258757 | | 2.188539 | -4.17497 | -0.45272 | | 2.029578 | 1.207366 | -0.32934 | | -0.43931 | -3.50434 | -1.24275 | | -2.6862 | -3.63863 | -1.98055 | | -3.76137 | -2.38742 | -1.34354 | | -3.195 | -2.28475 | -2.99848 | | 0.106521 | 2.4845 | -1.1208 | | -2.10878 | 3.485787 | -1.42669 | | -3.74932 | 2.211557 | -3.37092 | | -5.07394 | 1.410951 | -2.51903 | | -4.61918 | 3.06769 | -2.08534 | | -3.05793 | 1.054612 | 0.713087 | | -3.98733 | 2.503237 | 0.406225 | | -5.91097 | 1.115239 | -0.40899 | | -4.97113 | -0.34267 | -0.17727 | | -5.90051 | 1.622277 | 2.023231 | | -5.42772 | -0.5118 | 4.624945 | | -6.44794 | 0.835849 | 4.098899 | | -7.06927 | -0.82124 | 4.084334 | | -4.92538 | -1.92997 | 1.127396 | | -4.53583 | -2.18062 | 2.829041 | | -6.16711 | -2.48015 | 2.25637 | | 6.324516 | -2.83256 | 0.818007 | | 5.788233 | 0.364401 | 0.519041 | | 5.651732 | 2.922156 | 2.096922 | | 3.96405 | 3.249985 | 2.465859 | | 4.472014 | 1.642782 | -1.25961 | | 5.029005 | 0.650691 | 2.772149 | | 3.359552 | 0.906075 | 2.267665 | | | -4.41756 | 2.837043 | 1.004427 | | --- | --- | --- | | -4.94724 | 1.53995 | 0.925396 | | -4.18974 | 0.449271 | 0.482283 | | -2.86362 | 0.7317 | 0.122146 | | -2.30393 | 2.034707 | 0.192902 | | -3.09998 | 3.08787 | 0.641741 | | -1.88489 | -0.13647 | -0.3356 | | -0.71825 | 0.572005 | -0.59256 | | -0.93232 | 1.927853 | -0.26393 | | 0.507713 | 0.109972 | -1.0878 | | 1.531859 | 1.062123 | -1.21816 | | 1.352433 | 2.428075 | -0.91508 | | 0.111274 | 2.84235 | -0.43626 | | 2.483341 | 3.401861 | -1.1266 | | 0.776734 | -1.24989 | -1.53171 | | 2.025864 | -1.62956 | -1.82245 | | 3.191124 | -0.68708 | -1.62159 | | 2.744761 | 0.699983 | -1.73678 | | 4.224658 | -0.85115 | -2.73563 | | 3.789043 | -0.9103 | -0.21173 | | 5.02111 | -0.05634 | 0.141985 | | 5.376591 | -0.17101 | 1.599274 | | 6.490452 | -0.66493 | 2.155996 | | 6.656622 | -0.69647 | 3.655914 | | 7.665574 | -1.22168 | 1.392193 | | -6.24745 | 1.282435 | 1.301874 | | -4.78832 | -0.93719 | 0.458077 | | -4.49854 | -3.26325 | 1.121426 | | -4.27982 | -3.11502 | -0.38325 | | -4.32294 | -1.76682 | -0.66745 | | -4.10539 | -4.00084 | -1.19537 | | -4.40785 | -1.83255 | 1.669784 | | -5.04772 | 3.649012 | 1.358637 | | -2.70424 | 4.095998 | 0.710013 | | -2.07195 | -1.09183 | -0.59121 | | -0.03681 | 3.89137 | -0.19602 | | 2.807816 | 3.421987 | -2.17118 | | 2.180174 | 4.413328 | -0.84775 | | 3.36373 | 3.137876 | -0.53305 | | -0.04822 | -1.94566 | -1.65752 | | 2.245886 | -2.63107 | -2.17893 | | 5.002465 | -0.09 | -2.66185 | | 4.697064 | -1.83565 | -2.6765 | | 3.74733 | -0.75552 | -3.7128 | | 2.99272 | -0.72313 | 0.516966 | | 4.039617 | -1.97295 | -0.11324 | | 5.865393 | -0.33502 | -0.4914 | | 4.795704 | 0.991236 | -0.08955 | | 4.603872 | 0.195301 | 2.276575 | | 5.787738 | -0.28409 | 4.173188 | | 6.809698 | -1.72093 | 4.016452 | | 7.538339 | -0.12418 | 3.969063 | | 8.577213 | -0.65439 | 1.615139 | | 7.867384 | -2.25655 | 1.693287 | | 7.527495 | -1.21239 | 0.311109 | | -6.68832 | 2.105829 | 1.540223 | | -5.87694 | -0.83915 | 0.414891 | | -5.49341 | -3.69965 | 1.263744 | | -3.77829 | -3.96224 | 1.548794 | | -4.47934 | -1.48193 | -1.62552 | | -5.06315 | -1.64038 | 2.519739 | | -3.38515 | -1.60495 | 1.981421 | | | 4.576285 | -2.05253 | 1.693423 | | --- | --- | --- | | 4.89004 | -0.79916 | 1.14599 | | 3.963003 | -0.05224 | 0.409451 | | 2.695871 | -0.63346 | 0.252519 | | 2.35338 | -1.89982 | 0.795934 | | 3.312617 | -2.60435 | 1.522408 | | 1.585914 | -0.13076 | -0.40827 | | 0.546032 | -1.0485 | -0.33512 | | 0.978167 | -2.16069 | 0.418827 | | -0.74262 | -0.97255 | -0.87838 | | -1.60092 | -2.05248 | -0.61395 | | -1.20269 | -3.18646 | 0.124056 | | 0.093065 | -3.22159 | 0.634906 | | -2.16915 | -4.32374 | 0.334113 | | -1.23175 | 0.090904 | -1.74333 | | -2.53014 | 0.161887 | -2.05882 | | -3.52513 | -0.80986 | -1.46372 | | -2.85913 | -2.07421 | -1.14817 | | -4.60045 | -1.19759 | -2.47723 | | -4.12892 | -0.27759 | -0.14206 | | -4.87692 | 1.06603 | -0.23908 | | -5.47035 | 1.468916 | 1.084232 | | -5.14774 | 2.508929 | 1.864057 | | -5.85946 | 2.742278 | 3.174386 | | -4.08321 | 3.528878 | 1.547442 | | 6.135697 | -0.23893 | 1.328514 | | 4.331571 | 1.312949 | -0.12131 | | 3.681324 | 3.656389 | -0.26306 | | 3.467884 | 2.969801 | -1.61096 | | 3.724483 | 1.628109 | -1.4266 | | 3.140703 | 3.486877 | -2.65993 | | 3.828933 | 2.504376 | 0.740343 | | 5.332044 | -2.59043 | 2.260305 | | 3.084616 | -3.57398 | 1.953034 | | 1.61363 | 0.695757 | -0.9819 | | 0.409153 | -4.08774 | 1.209374 | | -1.71616 | -5.10976 | 0.942226 | | -3.08254 | -3.9902 | 0.835217 | | -2.48103 | -4.76857 | -0.61566 | | -0.52848 | 0.803564 | -2.16565 | | -2.90457 | 0.92334 | -2.73521 | | -4.14268 | -1.64776 | -3.36012 | | -5.29282 | -1.9212 | -2.04081 | | -5.17131 | -0.32325 | -2.79721 | | -4.80199 | -1.04985 | 0.247944 | | -3.31636 | -0.17577 | 0.584263 | | -4.20268 | 1.836122 | -0.61972 | | -5.69022 | 0.979499 | -0.97053 | | -6.25704 | 0.803719 | 1.442716 | | -6.62335 | 1.985873 | 3.366547 | | -5.15501 | 2.732163 | 4.014829 | | -6.34607 | 3.725031 | 3.191115 | | -3.332 | 3.561823 | 2.34538 | | -3.56049 | 3.338439 | 0.610395 | | -4.51467 | 4.535411 | 1.490404 | | 6.705767 | -0.85588 | 1.8013 | | 5.421188 | 1.365904 | -0.20213 | | 4.595457 | 4.254909 | -0.34453 | | 2.864662 | 4.346987 | -0.04829 | | 3.911291 | 1.057391 | -2.24078 | | 4.518074 | 2.713895 | 1.558842 | | 2.860356 | 2.250824 | 1.179012 | |

|  | 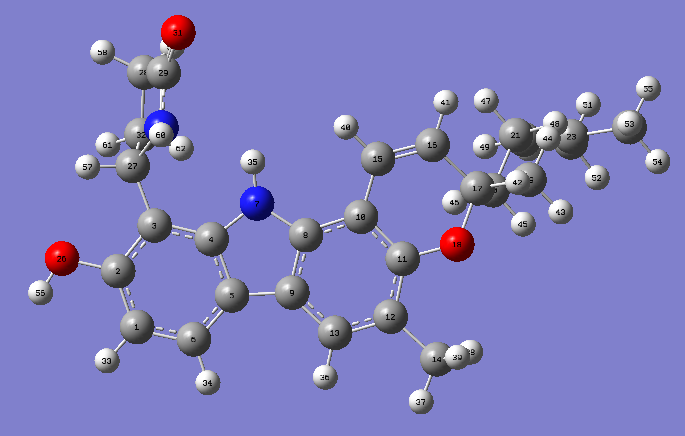  **Conformer (3ʹ*S*, 5ʹʹ*S*)-1 (12)** |
| --- | --- |
| | 1 | | --- | | 2 | | 3 | | 4 | | 5 | | 6 | | 7 | | 8 | | 9 | | 10 | | 11 | | 12 | | 13 | | 14 | | 15 | | 16 | | 17 | | 18 | | 19 | | 20 | | 21 | | 22 | | 23 | | 24 | | 25 | | 26 | | 27 | | 28 | | 29 | | 30 | | 31 | | 32 | | 33 | | 34 | | 35 | | 36 | | 37 | | 38 | | 39 | | 40 | | 41 | | 42 | | 43 | | 44 | | 45 | | 46 | | 47 | | 48 | | 49 | | 50 | | 51 | | 52 | | 53 | | 54 | | 55 | | 56 | | 57 | | 58 | | 59 | | 60 | | 61 | | 62 | | | -4.75275 | 2.189711 | 1.523663 | | --- | --- | --- | | -5.08698 | 0.884956 | 1.129903 | | -4.17547 | 0.043724 | 0.480887 | | -2.90226 | 0.585013 | 0.248505 | | -2.53916 | 1.901725 | 0.636562 | | -3.48345 | 2.700131 | 1.280866 | | -1.80416 | -0.00738 | -0.35532 | | -0.75193 | 0.89813 | -0.39747 | | -1.16371 | 2.097334 | 0.222622 | | 0.531588 | 0.74087 | -0.93562 | | 1.405657 | 1.832789 | -0.80597 | | 1.027687 | 3.051485 | -0.20501 | | -0.26344 | 3.164185 | 0.30632 | | 2.010056 | 4.192509 | -0.13723 | | 1.000531 | -0.42358 | -1.67197 | | 2.295805 | -0.54939 | -1.98375 | | 3.306961 | 0.471674 | -1.51113 | | 2.659802 | 1.77374 | -1.34679 | | 4.38379 | 0.720049 | -2.5658 | | 3.909691 | 0.092366 | -0.13713 | | 4.664368 | -1.2494 | -0.08447 | | 5.093742 | -1.58985 | 1.316984 | | 6.333591 | -1.72751 | 1.804755 | | 6.558534 | -2.06892 | 3.257683 | | 7.601369 | -1.56846 | 1.003851 | | -6.3383 | 0.36745 | 1.385077 | | -4.56562 | -1.36924 | 0.116867 | | -3.94576 | -3.72169 | 0.244242 | | -3.73857 | -3.20023 | -1.17666 | | -3.97563 | -1.8426 | -1.14789 | | -3.43 | -3.84074 | -2.16114 | | -4.06853 | -2.45844 | 1.107371 | | -5.49705 | 2.801056 | 2.027777 | | -3.23957 | 3.710507 | 1.592717 | | -1.84732 | -0.89517 | -0.82756 | | -0.56393 | 4.096276 | 0.776482 | | 1.571995 | 5.050765 | 0.376911 | | 2.923421 | 3.90825 | 0.393405 | | 2.319435 | 4.517994 | -1.13501 | | 0.285004 | -1.17097 | -2.00395 | | 2.654631 | -1.38963 | -2.56894 | | 3.929776 | 1.07249 | -3.49389 | | 5.091144 | 1.476701 | -2.21851 | | 4.936998 | -0.19635 | -2.78319 | | 4.580074 | 0.902291 | 0.170136 | | 3.091409 | 0.070995 | 0.590803 | | 4.002755 | -2.04539 | -0.44977 | | 5.520591 | -1.22631 | -0.76183 | | 4.266728 | -1.72845 | 2.01451 | | 5.618954 | -2.1771 | 3.803606 | | 7.119174 | -3.00576 | 3.362227 | | 7.153274 | -1.29564 | 3.758787 | | 7.429219 | -1.30316 | -0.03914 | | 8.24075 | -0.79334 | 1.442681 | | 8.188273 | -2.49465 | 1.018597 | | -6.89634 | 1.043382 | 1.786134 | | -5.65655 | -1.41702 | 0.052267 | | -4.86784 | -4.3135 | 0.241897 | | -3.13522 | -4.39321 | 0.530715 | | -4.16401 | -1.36836 | -2.02147 | | -4.75149 | -2.56165 | 1.950982 | | -3.0923 | -2.16848 | 1.504604 | |

**Table S8 Coordinates of (3ʹ*S*, 5ʹʹ*R*)-2**

|  | 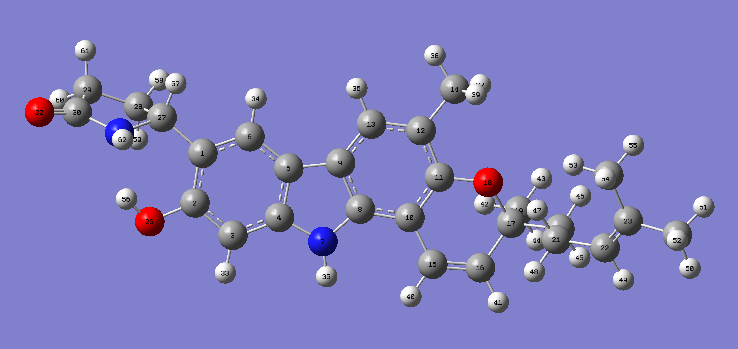  Conformer (3ʹ*S*, 5ʹʹ*R*)-**2** (13) | | 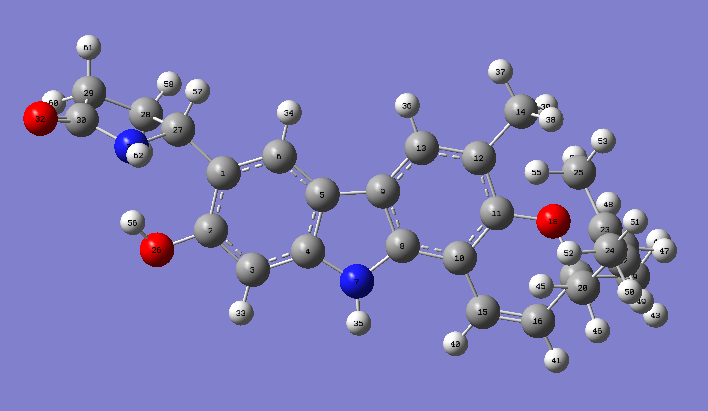  Conformer (3ʹ*S*, 5ʹʹ*R*)-**2** (22) | 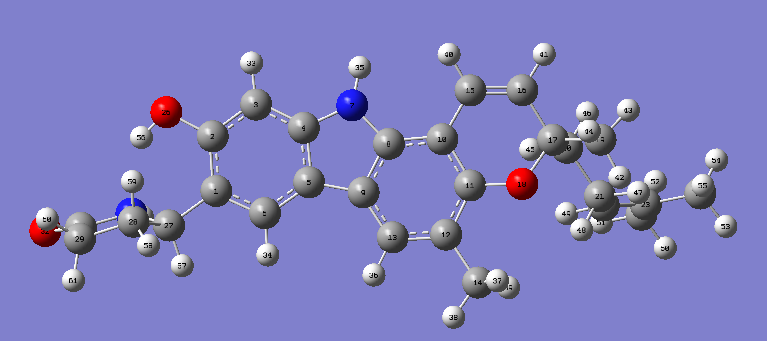  **Conformer (3ʹ*S*, 5ʹʹ*R*)-2 (23)** |
| --- | --- | --- | --- | --- |
| | 1 | | --- | | 2 | | 3 | | 4 | | 5 | | 6 | | 7 | | 8 | | 9 | | 10 | | 11 | | 12 | | 13 | | 14 | | 15 | | 16 | | 17 | | 18 | | 19 | | 20 | | 21 | | 22 | | 23 | | 24 | | 25 | | 26 | | 27 | | 28 | | 29 | | 30 | | 31 | | 32 | | 33 | | 34 | | 35 | | 36 | | 37 | | 38 | | 39 | | 40 | | 41 | | 42 | | 43 | | 44 | | 45 | | 46 | | 47 | | 48 | | 49 | | 50 | | 51 | | 52 | | 53 | | 54 | | 55 | | 56 | | 57 | | 58 | | 59 | | 60 | | 61 | | 62 | | | | 4.479357 | 0.147992 | -0.15492 | | --- | --- | --- | | 4.489506 | -1.13702 | -0.75569 | | 3.318096 | -1.87089 | -0.92182 | | 2.125113 | -1.31002 | -0.47936 | | 2.072071 | -0.02248 | 0.110223 | | 3.260578 | 0.691049 | 0.256243 | | 0.83797 | -1.82091 | -0.53033 | | -0.04776 | -0.90311 | 0.021696 | | 0.68117 | 0.23653 | 0.428704 | | -1.4362 | -0.99475 | 0.18074 | | -2.07376 | 0.099198 | 0.789834 | | -1.38399 | 1.259914 | 1.195366 | | -0.00406 | 1.309213 | 1.006514 | | -2.14007 | 2.409798 | 1.81034 | | -2.26367 | -2.09815 | -0.28306 | | -3.5538 | -2.16902 | 0.063337 | | -4.16126 | -1.15774 | 1.008735 | | -3.43268 | 0.106978 | 0.941639 | | -4.0919 | -1.67429 | 2.454275 | | -5.60938 | -0.80639 | 0.632363 | | -5.79186 | -0.22137 | -0.77873 | | -7.24058 | 0.014077 | -1.10852 | | -7.87326 | 1.177011 | -1.31345 | | -9.34804 | 1.210265 | -1.63252 | | -7.22036 | 2.535007 | -1.25537 | | 5.646507 | -1.72483 | -1.19064 | | 5.753527 | 0.91633 | 0.112171 | | 6.645622 | 0.363113 | 1.256949 | | 8.05624 | 0.857999 | 0.907025 | | 8.028592 | 1.076545 | -0.60403 | | 6.71163 | 0.914043 | -1.01595 | | 8.958534 | 1.349058 | -1.3295 | | 3.370462 | -2.84501 | -1.39564 | | 3.250017 | 1.681681 | 0.70339 | | 0.600927 | -2.7454 | -0.84727 | | 0.53811 | 2.196446 | 1.321281 | | -2.69303 | 2.099847 | 2.701758 | | -1.45719 | 3.212442 | 2.096975 | | -2.87707 | 2.826755 | 1.117251 | | -1.83363 | -2.84948 | -0.94003 | | -4.19051 | -2.97565 | -0.28694 | | -3.05533 | -1.85223 | 2.749485 | | -4.53222 | -0.94694 | 3.141619 | | -4.63682 | -2.61755 | 2.551223 | | -6.21235 | -1.71606 | 0.740468 | | -5.99176 | -0.09522 | 1.371433 | | -5.20262 | 0.692097 | -0.86133 | | -5.36882 | -0.92013 | -1.51214 | | -7.84096 | -0.89471 | -1.17615 | | -9.78285 | 0.208988 | -1.66876 | | -9.90201 | 1.792516 | -0.88626 | | -9.53337 | 1.692889 | -2.59982 | | -6.16113 | 2.497675 | -1.00268 | | -7.31697 | 3.053221 | -2.21701 | | -7.71751 | 3.170843 | -0.51319 | | 6.331619 | -1.04304 | -1.2939 | | 5.478688 | 1.952346 | 0.348937 | | 6.286969 | 0.701933 | 2.22963 | | 6.606772 | -0.72855 | 1.254053 | | 8.860877 | 0.173794 | 1.179967 | | 8.285792 | 1.823703 | 1.370636 | | 6.450245 | 1.318352 | -1.90714 | | | 4.073408 | 0.090153 | -0.01488 | | --- | --- | --- | | 4.122476 | -0.67685 | -1.20732 | | 2.994359 | -1.32701 | -1.70025 | | 1.805174 | -1.20497 | -0.99006 | | 1.71085 | -0.42887 | 0.191815 | | 2.85672 | 0.212951 | 0.658906 | | 0.558955 | -1.74973 | -1.25676 | | -0.34687 | -1.32885 | -0.29015 | | 0.333061 | -0.50891 | 0.637734 | | -1.70947 | -1.62495 | -0.16315 | | -2.38384 | -1.03717 | 0.920504 | | -1.7349 | -0.23644 | 1.882455 | | -0.37379 | 0.017478 | 1.722741 | | -2.50709 | 0.303111 | 3.058983 | | -2.45348 | -2.54322 | -1.01213 | | -3.78563 | -2.6209 | -0.91805 | | -4.55031 | -1.6953 | 0.001951 | | -3.70877 | -1.30385 | 1.131583 | | -5.73709 | -2.41638 | 0.639929 | | -4.96639 | -0.43105 | -0.78789 | | -5.78052 | 0.621826 | -0.01228 | | -6.09366 | 1.819507 | -0.86791 | | -5.67321 | 3.083511 | -0.72773 | | -6.09662 | 4.152123 | -1.70588 | | -4.76717 | 3.575215 | 0.372542 | | 5.278385 | -0.82905 | -1.92385 | | 5.311631 | 0.719257 | 0.582299 | | 6.31994 | -0.26799 | 1.23193 | | 7.666996 | 0.464791 | 1.15508 | | 7.512633 | 1.444025 | -0.00616 | | 6.181392 | 1.401576 | -0.40195 | | 8.366417 | 2.143843 | -0.5028 | | 3.075994 | -1.89916 | -2.61795 | | 2.814387 | 0.812763 | 1.564325 | | 0.32688 | -2.26591 | -2.08817 | | 0.134823 | 0.638052 | 2.455099 | | -1.8648 | 0.913851 | 3.697058 | | -3.35371 | 0.919303 | 2.742737 | | -2.92302 | -0.50286 | 3.671011 | | -1.9143 | -3.19875 | -1.69088 | | -4.3582 | -3.32316 | -1.51603 | | -6.21303 | -1.79197 | 1.396852 | | -6.48306 | -2.66945 | -0.11859 | | -5.40576 | -3.33914 | 1.120194 | | -4.05599 | 0.034626 | -1.17877 | | -5.53384 | -0.76153 | -1.66668 | | -6.72623 | 0.177558 | 0.320954 | | -5.23916 | 0.902521 | 0.892343 | | -6.74284 | 1.604845 | -1.71819 | | -6.75385 | 3.758984 | -2.48455 | | -6.62627 | 4.966897 | -1.19752 | | -5.22732 | 4.607234 | -2.19567 | | -5.25969 | 4.360242 | 0.958843 | | -4.45463 | 2.791885 | 1.062307 | | -3.86116 | 4.027773 | -0.04723 | | 5.906675 | -0.13343 | -1.66624 | | 4.989363 | 1.443849 | 1.341353 | | 6.01441 | -0.52587 | 2.246604 | | 6.348867 | -1.19341 | 0.65202 | | 8.530489 | -0.18283 | 0.99685 | | 7.871276 | 1.056803 | 2.053975 | | 5.829686 | 2.189297 | -0.9328 | | | 4.305493 | 0.207734 | -0.03436 | | --- | --- | --- | | 4.329221 | -0.41061 | -1.31082 | | 3.188947 | -0.99379 | -1.85697 | | 2.014042 | -0.95638 | -1.11414 | | 1.945673 | -0.32955 | 0.154997 | | 3.102434 | 0.250412 | 0.672977 | | 0.76116 | -1.46449 | -1.41925 | | -0.12248 | -1.16882 | -0.3878 | | 0.577967 | -0.46603 | 0.617628 | | -1.48061 | -1.4863 | -0.26507 | | -2.12809 | -1.04613 | 0.901528 | | -1.46014 | -0.35807 | 1.934987 | | -0.10444 | -0.07704 | 1.77389 | | -2.20724 | 0.040204 | 3.18196 | | -2.2447 | -2.2875 | -1.20949 | | -3.57295 | -2.39015 | -1.08929 | | -4.31827 | -1.61745 | -0.02439 | | -3.44312 | -1.35681 | 1.116326 | | -5.46207 | -2.45122 | 0.552465 | | -4.79784 | -0.26855 | -0.6121 | | -5.61888 | 0.625519 | 0.33619 | | -5.83147 | 1.998637 | -0.24042 | | -6.97894 | 2.59331 | -0.59289 | | -6.98237 | 3.990367 | -1.16434 | | -8.34826 | 1.973768 | -0.46785 | | 5.471176 | -0.47931 | -2.06195 | | 5.556727 | 0.756526 | 0.612542 | | 6.568611 | -0.306 | 1.122243 | | 7.918219 | 0.425444 | 1.115599 | | 7.753034 | 1.537897 | 0.082674 | | 6.415862 | 1.548707 | -0.2957 | | 8.603678 | 2.288824 | -0.339 | | 3.250724 | -1.45173 | -2.83812 | | 3.079799 | 0.736527 | 1.644848 | | 0.51231 | -1.88301 | -2.29921 | | 0.419109 | 0.456029 | 2.562522 | | -2.62539 | -0.82977 | 3.6969 | | -1.54673 | 0.559807 | 3.879461 | | -3.04866 | 0.702656 | 2.957741 | | -1.72294 | -2.83526 | -1.98977 | | -4.15887 | -3.00611 | -1.76444 | | -5.9202 | -1.94862 | 1.405199 | | -6.23283 | -2.61931 | -0.20503 | | -5.09186 | -3.42144 | 0.88945 | | -3.91091 | 0.277064 | -0.9523 | | -5.3832 | -0.47885 | -1.51495 | | -6.57105 | 0.146857 | 0.573035 | | -5.07634 | 0.716059 | 1.284489 | | -4.91184 | 2.565558 | -0.39148 | | -7.58331 | 4.669327 | -0.54723 | | -5.97517 | 4.406088 | -1.23727 | | -7.42856 | 4.007528 | -2.16608 | | -8.98979 | 2.578636 | 0.184147 | | -8.84775 | 1.940351 | -1.44339 | | -8.33434 | 0.9598 | -0.06822 | | 6.109102 | 0.176755 | -1.73401 | | 5.25028 | 1.385828 | 1.458125 | | 6.276015 | -0.68473 | 2.102265 | | 6.584569 | -1.15365 | 0.433292 | | 8.776114 | -0.20161 | 0.868678 | | 8.137686 | 0.90421 | 2.076279 | | 6.061212 | 2.396456 | -0.72184 | |

|  | 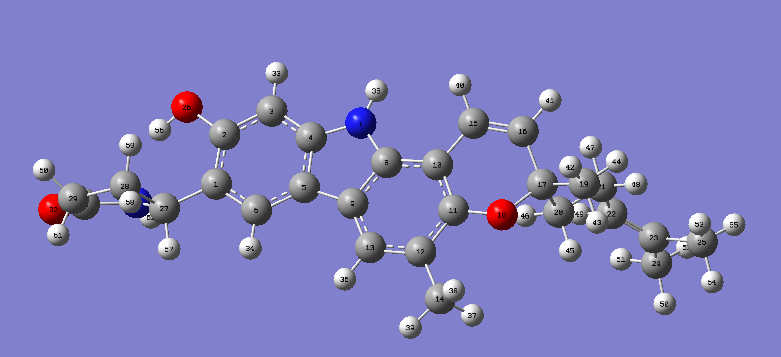  Conformer (3ʹ*S*, 5ʹʹ*R*)-**2** (33) | 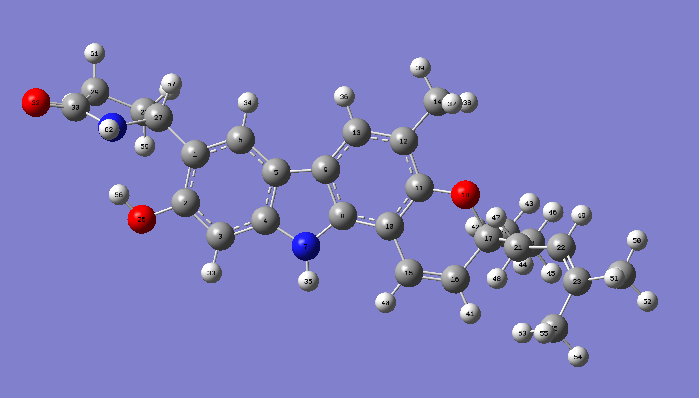  Conformer (3ʹ*S*, 5ʹʹ*R*)-**2** (41) | 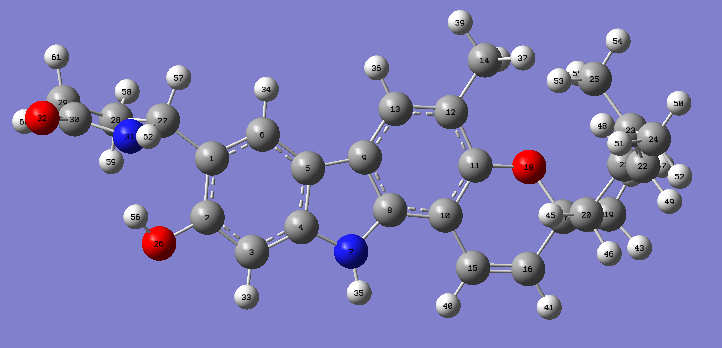  **Conformer (3ʹ*S*, 5ʹʹ*R*)-2 (58)** |
| --- | --- | --- | --- |
| | 1 | | --- | | 2 | | 3 | | 4 | | 5 | | 6 | | 7 | | 8 | | 9 | | 10 | | 11 | | 12 | | 13 | | 14 | | 15 | | 16 | | 17 | | 18 | | 19 | | 20 | | 21 | | 22 | | 23 | | 24 | | 25 | | 26 | | 27 | | 28 | | 29 | | 30 | | 31 | | 32 | | 33 | | 34 | | 35 | | 36 | | 37 | | 38 | | 39 | | 40 | | 41 | | 42 | | 43 | | 44 | | 45 | | 46 | | 47 | | 48 | | 49 | | 50 | | 51 | | 52 | | 53 | | 54 | | 55 | | 56 | | 57 | | 58 | | 59 | | 60 | | 61 | | 62 | | | -4.34235 | 0.201171 | -0.06076 | | --- | --- | --- | | -4.23532 | 0.690696 | 1.266389 | | -3.03582 | 0.626477 | 1.970145 | | -1.93286 | 0.066756 | 1.334912 | | -1.99433 | -0.41258 | 0.002759 | | -3.20846 | -0.33171 | -0.67716 | | -0.64319 | -0.11862 | 1.807864 | | 0.138892 | -0.68705 | 0.809255 | | -0.66766 | -0.89376 | -0.33199 | | 1.492281 | -1.04415 | 0.838002 | | 2.023596 | -1.59734 | -0.3394 | | 1.247985 | -1.83544 | -1.49205 | | -0.09837 | -1.4757 | -1.4686 | | 1.878728 | -2.47114 | -2.70436 | | 2.362127 | -0.9581 | 2.001171 | | 3.6809 | -1.14858 | 1.875962 | | 4.30095 | -1.37989 | 0.515517 | | 3.326062 | -2.01188 | -0.37343 | | 5.446303 | -2.3881 | 0.590903 | | 4.732193 | -0.05454 | -0.1573 | | 5.776366 | 0.778108 | 0.610094 | | 6.037156 | 2.09833 | -0.06331 | | 7.174364 | 2.553695 | -0.60509 | | 7.232396 | 3.918784 | -1.24626 | | 8.479009 | 1.798324 | -0.64111 | | -5.30334 | 1.235788 | 1.926346 | | -5.66054 | 0.17765 | -0.80016 | | -6.69016 | -0.87421 | -0.30436 | | -8.04954 | -0.29409 | -0.71932 | | -7.81615 | 1.21141 | -0.82016 | | -6.45039 | 1.42337 | -0.67506 | | -8.64178 | 2.078115 | -1.00043 | | -2.99746 | 1.016893 | 2.981227 | | -3.28543 | -0.69353 | -1.69914 | | -0.30439 | 0.221123 | 2.691792 | | -0.70387 | -1.64739 | -2.35412 | | 2.729996 | -1.88869 | -3.06846 | | 2.259819 | -3.47256 | -2.48256 | | 1.154628 | -2.55838 | -3.51731 | | 1.926003 | -0.78612 | 2.981788 | | 4.337272 | -1.13421 | 2.739877 | | 5.087412 | -3.33949 | 0.988144 | | 5.865196 | -2.56493 | -0.40244 | | 6.243897 | -2.02485 | 1.24258 | | 5.116122 | -0.2955 | -1.15438 | | 3.83196 | 0.551262 | -0.3072 | | 5.402861 | 0.971347 | 1.624073 | | 6.700756 | 0.208327 | 0.726637 | | 5.165388 | 2.751834 | -0.11416 | | 7.526688 | 3.848849 | -2.3004 | | 6.271653 | 4.435693 | -1.1985 | | 7.981584 | 4.553854 | -0.75818 | | 8.423557 | 0.806715 | -0.19208 | | 8.825919 | 1.676021 | -1.67397 | | 9.264597 | 2.356465 | -0.11789 | | -5.98365 | 1.48754 | 1.279252 | | -5.44891 | -0.0091 | -1.86099 | | -6.48029 | -1.85647 | -0.7293 | | -6.62366 | -0.96452 | 0.782301 | | -8.86735 | -0.50923 | -0.03012 | | -8.36591 | -0.64054 | -1.70938 | | -6.07708 | 2.299966 | -1.01885 | | | 4.524188 | -0.09468 | -0.19691 | | --- | --- | --- | | 4.432131 | -1.41109 | 0.323805 | | 3.211142 | -1.95494 | 0.713136 | | 2.071328 | -1.16985 | 0.578119 | | 2.120452 | 0.142752 | 0.046459 | | 3.356697 | 0.657215 | -0.34102 | | 0.752391 | -1.47059 | 0.879244 | | -0.05381 | -0.38139 | 0.57034 | | 0.760119 | 0.64524 | 0.041952 | | -1.43973 | -0.23556 | 0.709532 | | -1.9848 | 0.999152 | 0.319307 | | -1.20859 | 2.042351 | -0.22544 | | 0.164768 | 1.845715 | -0.35649 | | -1.86998 | 3.326586 | -0.65579 | | -2.35244 | -1.27376 | 1.163997 | | -3.63326 | -0.98428 | 1.419474 | | -4.14163 | 0.436468 | 1.326119 | | -3.33464 | 1.20697 | 0.383963 | | -4.05921 | 1.122282 | 2.699056 | | -5.57514 | 0.507444 | 0.77631 | | -5.7735 | -0.12391 | -0.61235 | | -7.16922 | 0.087298 | -1.13184 | | -8.10562 | -0.82621 | -1.42026 | | -9.45984 | -0.40921 | -1.94035 | | -7.93154 | -2.31714 | -1.27481 | | 5.534716 | -2.20669 | 0.480326 | | 5.854048 | 0.540248 | -0.53407 | | 6.740463 | 0.92837 | 0.680905 | | 8.169816 | 0.92074 | 0.120459 | | 8.112742 | -0.02194 | -1.07935 | | 6.776219 | -0.34292 | -1.28272 | | 9.037122 | -0.42309 | -1.7501 | | 3.185252 | -2.96949 | 1.095496 | | 3.424594 | 1.66163 | -0.75076 | | 0.447276 | -2.31322 | 1.335532 | | 0.773334 | 2.645099 | -0.76975 | | -2.58995 | 3.160205 | -1.46292 | | -2.42534 | 3.789549 | 0.164922 | | -1.12745 | 4.04453 | -1.01072 | | -1.99365 | -2.29479 | 1.262863 | | -4.33363 | -1.74848 | 1.742225 | | -3.02728 | 1.144789 | 3.056783 | | -4.43014 | 2.148899 | 2.636134 | | -4.65956 | 0.581038 | 3.435642 | | -6.24132 | 0.023897 | 1.499844 | | -5.86857 | 1.562259 | 0.745312 | | -5.05919 | 0.338504 | -1.30257 | | -5.51605 | -1.18498 | -0.577 | | -7.43531 | 1.134494 | -1.2836 | | -9.54786 | 0.675561 | -2.03061 | | -9.65679 | -0.84642 | -2.92681 | | -10.2636 | -0.75999 | -1.28169 | | -6.95747 | -2.60565 | -0.87996 | | -8.69802 | -2.73313 | -0.61002 | | -8.0592 | -2.81937 | -2.24125 | | 6.257879 | -1.86007 | -0.06916 | | 5.656629 | 1.441654 | -1.12866 | | 6.433703 | 1.888682 | 1.097006 | | 6.628869 | 0.177844 | 1.466781 | | 8.935456 | 0.595823 | 0.82637 | | 8.475687 | 1.90405 | -0.25353 | | 6.516326 | -0.69334 | -2.19692 | | | 4.07348 | 0.090179 | -0.01488 | | --- | --- | --- | | 4.122561 | -0.67691 | -1.20727 | | 2.99446 | -1.32714 | -1.70014 | | 1.805275 | -1.20508 | -0.98996 | | 1.710935 | -0.42889 | 0.191856 | | 2.856792 | 0.21299 | 0.658898 | | 0.559076 | -1.74993 | -1.2566 | | -0.34677 | -1.32892 | -0.29006 | | 0.333141 | -0.50891 | 0.637768 | | -1.70938 | -1.62501 | -0.16306 | | -2.38377 | -1.03714 | 0.920532 | | -1.73484 | -0.23635 | 1.882438 | | -0.37372 | 0.017555 | 1.722731 | | -2.50706 | 0.303339 | 3.058885 | | -2.45337 | -2.54335 | -1.01198 | | -3.78552 | -2.62102 | -0.91791 | | -4.55023 | -1.69536 | 0.002008 | | -3.7087 | -1.30377 | 1.131603 | | -5.73698 | -2.41642 | 0.64005 | | -4.96636 | -0.43119 | -0.78795 | | -5.78053 | 0.621715 | -0.01242 | | -6.09369 | 1.819326 | -0.86814 | | -5.67361 | 3.083439 | -0.72782 | | -6.09701 | 4.151948 | -1.70609 | | -4.76803 | 3.575374 | 0.372723 | | 5.278474 | -0.82914 | -1.92379 | | 5.311688 | 0.719368 | 0.582237 | | 6.320024 | -0.26779 | 1.231952 | | 7.667053 | 0.465037 | 1.155056 | | 7.512666 | 1.444167 | -0.00627 | | 6.181429 | 1.401623 | -0.40208 | | 8.366427 | 2.143981 | -0.50295 | | 3.07611 | -1.89936 | -2.6178 | | 2.814444 | 0.812868 | 1.564274 | | 0.326936 | -2.26586 | -2.08815 | | 0.134883 | 0.63817 | 2.455059 | | -3.35331 | 0.920002 | 2.742554 | | -2.92351 | -0.50255 | 3.670657 | | -1.86464 | 0.913668 | 3.697224 | | -1.91418 | -3.19893 | -1.69066 | | -4.35809 | -3.32334 | -1.51585 | | -6.21293 | -1.79196 | 1.396931 | | -6.48296 | -2.66957 | -0.11845 | | -5.40563 | -3.33914 | 1.120381 | | -4.05596 | 0.034482 | -1.17886 | | -5.53379 | -0.76177 | -1.66671 | | -6.72623 | 0.177441 | 0.320826 | | -5.23919 | 0.902487 | 0.892185 | | -6.74257 | 1.604508 | -1.71862 | | -6.62702 | 4.966581 | -1.19789 | | -5.22768 | 4.60729 | -2.19562 | | -6.75391 | 3.758645 | -2.48496 | | -3.86205 | 4.02826 | -0.04677 | | -5.26096 | 4.360202 | 0.958941 | | -4.45542 | 2.792104 | 1.062529 | | 5.906737 | -0.13347 | -1.66623 | | 4.989398 | 1.444023 | 1.341223 | | 6.014496 | -0.5256 | 2.246645 | | 6.348992 | -1.19325 | 0.65212 | | 8.530575 | -0.18257 | 0.996895 | | 7.871299 | 1.057134 | 2.053904 | | 5.829694 | 2.189303 | -0.93298 | |

**Table S9 Coordinates of (3ʹ*S*, 5ʹʹ*S*)-2**

|  | 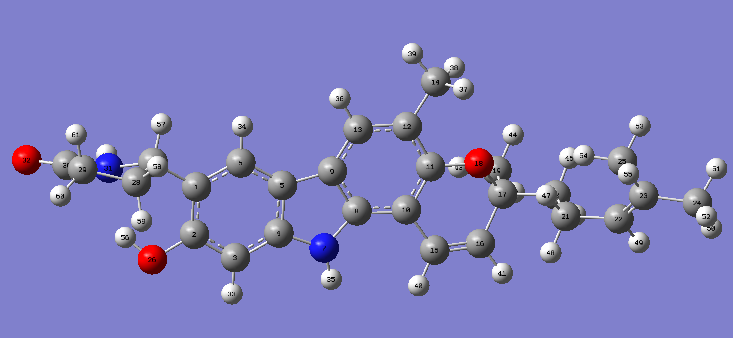  Conformer (3ʹ*S*, 5ʹʹ*S*)-**2** (13) | 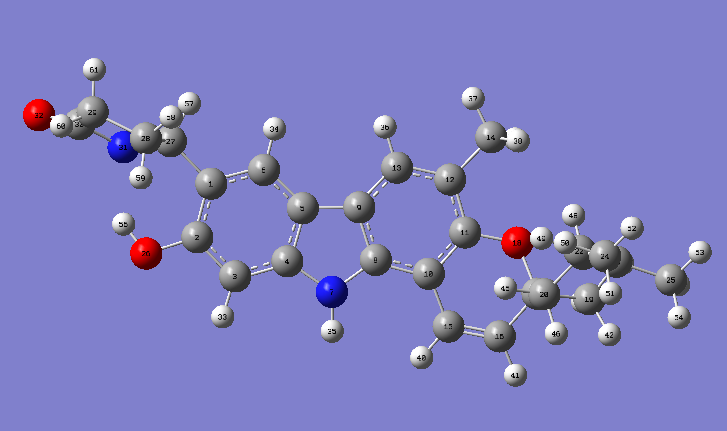  Conformer (3ʹ*S*, 5ʹʹ*S*)-**2** (23) | 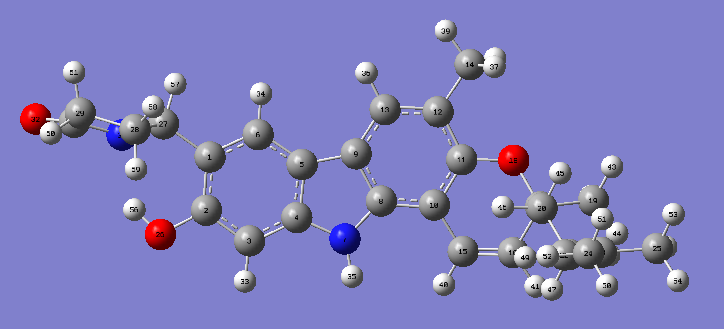  **Conformer (3ʹ*S*, 5ʹʹ*S*)-2 (34)** |
| --- | --- | --- | --- |
| | 1 | | --- | | 2 | | 3 | | 4 | | 5 | | 6 | | 7 | | 8 | | 9 | | 10 | | 11 | | 12 | | 13 | | 14 | | 15 | | 16 | | 17 | | 18 | | 19 | | 20 | | 21 | | 22 | | 23 | | 24 | | 25 | | 26 | | 27 | | 28 | | 29 | | 30 | | 31 | | 32 | | 33 | | 34 | | 35 | | 36 | | 37 | | 38 | | 39 | | 40 | | 41 | | 42 | | 43 | | 44 | | 45 | | 46 | | 47 | | 48 | | 49 | | 50 | | 51 | | 52 | | 53 | | 54 | | 55 | | 56 | | 57 | | 58 | | 59 | | 60 | | 61 | | 62 | | | 4.487539 | 0.130116 | -0.07376 | | --- | --- | --- | | 4.50176 | -1.11171 | -0.75957 | | 3.329239 | -1.82236 | -1.00051 | | 2.131009 | -1.28207 | -0.54713 | | 2.078224 | -0.05562 | 0.160921 | | 3.269547 | 0.631222 | 0.389746 | | 0.841977 | -1.77801 | -0.66045 | | -0.04419 | -0.91415 | -0.02784 | | 0.685726 | 0.177417 | 0.493177 | | -1.43394 | -1.01526 | 0.111447 | | -2.07204 | 0.015638 | 0.821552 | | -1.38148 | 1.128655 | 1.342993 | | -0.00032 | 1.191833 | 1.167555 | | -2.13926 | 2.215058 | 2.062358 | | -2.26202 | -2.06304 | -0.4663 | | -3.55366 | -2.16479 | -0.13356 | | -4.1603 | -1.25328 | 0.908877 | | -3.43179 | 0.011768 | 0.966343 | | -4.08935 | -1.9107 | 2.296049 | | -5.60904 | -0.86674 | 0.571391 | | -5.79793 | -0.16127 | -0.78236 | | -7.24754 | 0.109322 | -1.07923 | | -7.86875 | 1.28882 | -1.21168 | | -9.34683 | 1.355992 | -1.50981 | | -7.1997 | 2.634659 | -1.08909 | | 5.660979 | -1.66631 | -1.23045 | | 5.741261 | 0.954544 | 0.110631 | | 6.292095 | 1.637107 | -1.17136 | | 7.78977 | 1.820789 | -0.88717 | | 8.123531 | 0.752718 | 0.151742 | | 6.922455 | 0.174097 | 0.542487 | | 9.215386 | 0.450928 | 0.578631 | | 3.386738 | -2.77017 | -1.52446 | | 3.259384 | 1.574858 | 0.92894 | | 0.606927 | -2.67656 | -1.04618 | | 0.542467 | 2.041893 | 1.5713 | | -2.86237 | 2.707255 | 1.404847 | | -2.70861 | 1.818654 | 2.907932 | | -1.45595 | 2.978207 | 2.441113 | | -1.83115 | -2.74261 | -1.19675 | | -4.19208 | -2.92796 | -0.56789 | | -4.63095 | -2.86082 | 2.298772 | | -3.05234 | -2.11371 | 2.572871 | | -4.53224 | -1.25693 | 3.052166 | | -6.21377 | -1.7812 | 0.602763 | | -5.98572 | -0.22214 | 1.371877 | | -5.20422 | 0.753055 | -0.79176 | | -5.3849 | -0.79691 | -1.57656 | | -7.85943 | -0.78737 | -1.19103 | | -9.79432 | 0.363246 | -1.59427 | | -9.88459 | 1.903415 | -0.72629 | | -9.53779 | 1.892475 | -2.44714 | | -7.68157 | 3.235894 | -0.30899 | | -6.13812 | 2.573163 | -0.85148 | | -7.30107 | 3.203525 | -2.02113 | | 6.417199 | -1.258 | -0.7763 | | 5.528523 | 1.729176 | 0.85878 | | 5.763872 | 2.569325 | -1.375 | | 6.139187 | 0.978422 | -2.02935 | | 8.435357 | 1.718765 | -1.76063 | | 8.012904 | 2.794008 | -0.43635 | | 6.91072 | -0.32227 | 1.425323 | | | 4.303575 | 0.123057 | 0.009249 | | --- | --- | --- | | 4.338416 | -0.57905 | -1.2229 | | 3.200011 | -1.1862 | -1.74609 | | 2.01453 | -1.08603 | -1.0262 | | 1.943285 | -0.40666 | 0.215399 | | 3.101729 | 0.184238 | 0.717259 | | 0.760081 | -1.59809 | -1.31734 | | -0.12726 | -1.25482 | -0.30419 | | 0.572579 | -0.51683 | 0.676241 | | -1.48933 | -1.55402 | -0.17877 | | -2.14134 | -1.05739 | 0.962464 | | -1.47383 | -0.33504 | 1.972502 | | -0.11427 | -0.07403 | 1.810346 | | -2.2265 | 0.122374 | 3.195586 | | -2.25456 | -2.38778 | -1.09368 | | -3.58522 | -2.46751 | -0.98249 | | -4.32855 | -1.63363 | 0.036796 | | -3.46236 | -1.34213 | 1.176903 | | -5.4982 | -2.41575 | 0.633369 | | -4.77005 | -0.30179 | -0.61629 | | -5.58048 | 0.651858 | 0.281688 | | -5.7447 | 2.006903 | -0.3508 | | -6.8705 | 2.628729 | -0.72547 | | -6.82429 | 4.002544 | -1.34915 | | -8.26122 | 2.064976 | -0.57556 | | 5.484054 | -0.68549 | -1.96408 | | 5.50869 | 0.864015 | 0.542213 | | 5.884131 | 2.168334 | -0.21293 | | 7.383397 | 2.34505 | 0.065706 | | 7.891734 | 0.937473 | 0.369254 | | 6.77844 | 0.109313 | 0.446304 | | 9.038198 | 0.582419 | 0.526691 | | 3.274559 | -1.7224 | -2.68582 | | 3.076226 | 0.711406 | 1.667411 | | 0.516369 | -2.06321 | -2.17503 | | 0.408963 | 0.485763 | 2.580444 | | -1.56554 | 0.661442 | 3.877727 | | -3.05725 | 0.78567 | 2.936867 | | -2.66028 | -0.72082 | 3.741197 | | -1.73259 | -2.97824 | -1.84204 | | -4.17307 | -3.1068 | -1.63385 | | -6.25996 | -2.60456 | -0.12831 | | -5.1538 | -3.37586 | 1.022647 | | -5.95921 | -1.86316 | 1.452962 | | -3.86665 | 0.207612 | -0.9691 | | -5.34957 | -0.53884 | -1.51626 | | -6.54956 | 0.209701 | 0.520972 | | -5.05077 | 0.764805 | 1.234884 | | -4.80555 | 2.534363 | -0.5234 | | -5.80295 | 4.379046 | -1.437 | | -7.27051 | 3.9983 | -2.35102 | | -7.40002 | 4.725121 | -0.75809 | | -8.87676 | 2.713952 | 0.058897 | | -8.76632 | 2.01862 | -1.54764 | | -8.28273 | 1.064782 | -0.14303 | | 6.249412 | -0.48804 | -1.3982 | | 5.314799 | 1.109788 | 1.594381 | | 5.270407 | 3.00473 | 0.123622 | | 5.702528 | 2.035772 | -1.28194 | | 7.951032 | 2.788381 | -0.75366 | | 7.572704 | 2.957284 | 0.954321 | | 6.886568 | -0.76973 | 0.93784 | | | 4.357016 | 0.123736 | 0.023942 | | --- | --- | --- | | 4.255593 | 0.473602 | -1.34702 | | 3.052474 | 0.361605 | -2.03851 | | 1.939785 | -0.10343 | -1.34644 | | 2.004265 | -0.47571 | 0.019386 | | 3.225097 | -0.3611 | 0.682287 | | 0.64539 | -0.31148 | -1.79611 | | -0.13596 | -0.79231 | -0.75217 | | 0.675235 | -0.91665 | 0.397619 | | -1.4925 | -1.13847 | -0.74643 | | -2.02263 | -1.59154 | 0.473543 | | -1.24265 | -1.74549 | 1.637607 | | 0.106832 | -1.40172 | 1.579328 | | -1.87186 | -2.27725 | 2.899788 | | -2.36583 | -1.14195 | -1.91007 | | -3.68507 | -1.3146 | -1.76623 | | -4.30225 | -1.42825 | -0.38979 | | -3.32846 | -1.98983 | 0.546673 | | -5.45363 | -2.43234 | -0.37751 | | -4.72376 | -0.04783 | 0.16906 | | -5.76325 | 0.721668 | -0.66737 | | -6.01036 | 2.100434 | -0.11749 | | -7.14721 | 2.620663 | 0.363183 | | -7.1906 | 4.038747 | 0.878185 | | -8.46579 | 1.893425 | 0.44386 | | 5.326121 | 0.948189 | -2.05564 | | 5.633812 | 0.324565 | 0.807998 | | 5.994812 | 1.798795 | 1.137123 | | 7.519077 | 1.781143 | 1.318443 | | 7.993199 | 0.574633 | 0.511859 | | 6.857822 | -0.11206 | 0.099044 | | 9.133328 | 0.248071 | 0.269372 | | 3.022818 | 0.628899 | -3.08915 | | 3.304353 | -0.64096 | 1.72952 | | 0.307218 | -0.05106 | -2.70674 | | 0.715892 | -1.51018 | 2.472403 | | -2.71052 | -1.65531 | 3.226219 | | -2.27047 | -3.28627 | 2.758018 | | -1.1415 | -2.31423 | 3.710988 | | -1.93207 | -1.05394 | -2.90281 | | -4.34373 | -1.36892 | -2.62678 | | -5.10113 | -3.41584 | -0.69406 | | -5.87061 | -2.52266 | 0.628142 | | -6.25131 | -2.1209 | -1.05546 | | -5.10768 | -0.19948 | 1.183579 | | -3.81962 | 0.563139 | 0.265592 | | -5.39197 | 0.817832 | -1.696 | | -6.69308 | 0.152345 | -0.72855 | | -5.12838 | 2.742162 | -0.11217 | | -7.91915 | 4.640214 | 0.321086 | | -7.50406 | 4.06944 | 1.92862 | | -6.21954 | 4.532689 | 0.802588 | | -8.83372 | 1.873937 | 1.476517 | | -9.2314 | 2.41313 | -0.14454 | | -8.4199 | 0.863727 | 0.089629 | | 6.145975 | 0.721683 | -1.58503 | | 5.543903 | -0.23473 | 1.748275 | | 5.449078 | 2.147563 | 2.014643 | | 5.71252 | 2.438845 | 0.298018 | | 8.028742 | 2.687045 | 0.987291 | | 7.812838 | 1.611001 | 2.360122 | | 6.972975 | -1.08209 | -0.16891 | |

|  | 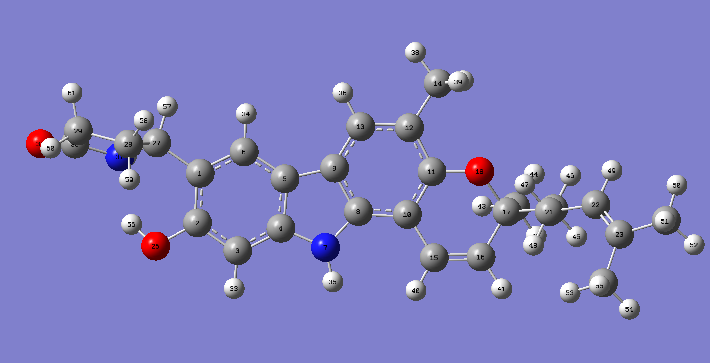  Conformer (3ʹ*S*, 5ʹʹ*S*)-**2** (43) | 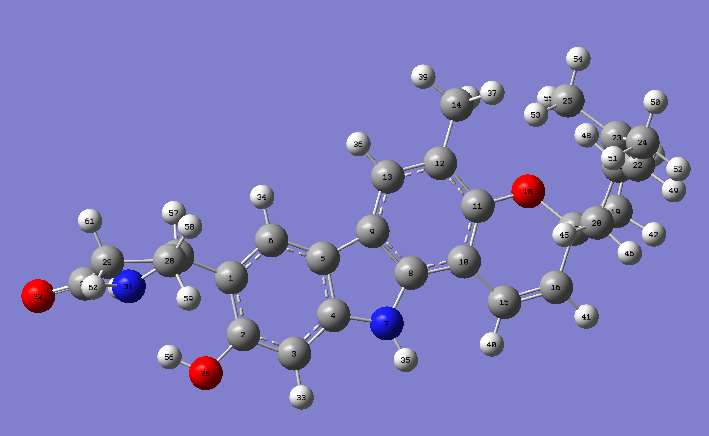  Conformer (3ʹ*S*, 5ʹʹ*S*)-**2** (58) | 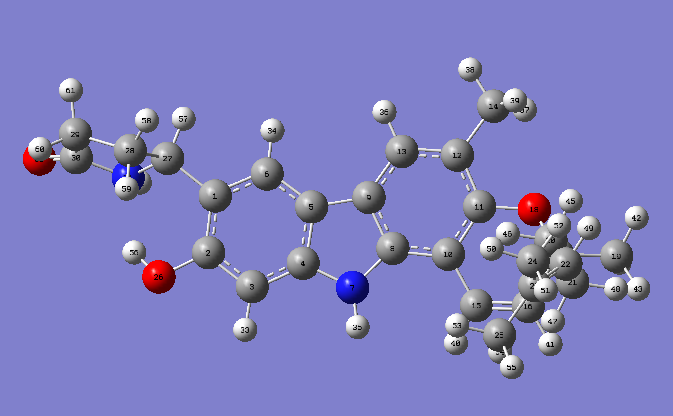  **Conformer (3ʹ*S*, 5ʹʹ*S*)-2 (75)** |
| --- | --- | --- | --- |
| | 1 | | --- | | 2 | | 3 | | 4 | | 5 | | 6 | | 7 | | 8 | | 9 | | 10 | | 11 | | 12 | | 13 | | 14 | | 15 | | 16 | | 17 | | 18 | | 19 | | 20 | | 21 | | 22 | | 23 | | 24 | | 25 | | 26 | | 27 | | 28 | | 29 | | 30 | | 31 | | 32 | | 33 | | 34 | | 35 | | 36 | | 37 | | 38 | | 39 | | 40 | | 41 | | 42 | | 43 | | 44 | | 45 | | 46 | | 47 | | 48 | | 49 | | 50 | | 51 | | 52 | | 53 | | 54 | | 55 | | 56 | | 57 | | 58 | | 59 | | 60 | | 61 | | 62 | | | -4.52605 | -0.0451 | 0.126785 | | --- | --- | --- | | -4.43984 | -1.40604 | -0.26387 | | -3.21877 | -1.99582 | -0.57927 | | -2.07332 | -1.21117 | -0.50361 | | -2.12145 | 0.157431 | -0.13923 | | -3.35911 | 0.720458 | 0.167616 | | -0.75361 | -1.55035 | -0.75632 | | 0.05422 | -0.43316 | -0.58008 | | -0.75959 | 0.653526 | -0.1898 | | 1.441488 | -0.3096 | -0.72615 | | 1.989049 | 0.962057 | -0.48684 | | 1.212825 | 2.067179 | -0.08232 | | -0.1623 | 1.892346 | 0.061165 | | 1.875636 | 3.393404 | 0.188918 | | 2.352586 | -1.39819 | -1.04653 | | 3.636146 | -1.14627 | -1.32644 | | 4.151862 | 0.273169 | -1.39843 | | 3.340379 | 1.15556 | -0.56434 | | 4.087699 | 0.788699 | -2.84466 | | 5.580306 | 0.400953 | -0.8449 | | 5.755253 | -0.04295 | 0.617113 | | 7.150469 | 0.212507 | 1.117798 | | 8.057953 | -0.6662 | 1.5637 | | 9.417478 | -0.20517 | 2.030254 | | 7.843195 | -2.15575 | 1.661652 | | -5.54464 | -2.21058 | -0.33681 | | -5.83069 | 0.580121 | 0.565113 | | -6.36059 | 0.125668 | 1.952785 | | -7.87637 | 0.354181 | 1.869545 | | -8.19762 | 0.300305 | 0.377808 | | -6.98521 | 0.246721 | -0.2991 | | -9.28927 | 0.310033 | -0.14515 | | -3.19972 | -3.03816 | -0.87821 | | -3.42526 | 1.766824 | 0.453999 | | -0.44905 | -2.44316 | -1.10498 | | -0.77083 | 2.738935 | 0.366411 | | 2.418645 | 3.760397 | -0.68693 | | 1.135451 | 4.14622 | 0.468528 | | 2.606637 | 3.321916 | 0.999892 | | 1.989555 | -2.42233 | -1.02645 | | 4.334278 | -1.94622 | -1.55309 | | 4.693589 | 0.160799 | -3.50392 | | 3.059703 | 0.770818 | -3.2138 | | 4.46208 | 1.814258 | -2.90179 | | 6.248029 | -0.18101 | -1.49039 | | 5.887086 | 1.447337 | -0.94887 | | 5.042827 | 0.519351 | 1.230892 | | 5.478482 | -1.09451 | 0.721424 | | 7.443495 | 1.263458 | 1.105798 | | 9.536504 | 0.876741 | 1.940434 | | 9.589488 | -0.47686 | 3.078832 | | 10.21853 | -0.68219 | 1.452599 | | 6.863983 | -2.47872 | 1.308773 | | 8.602462 | -2.69577 | 1.083407 | | 7.949712 | -2.49459 | 2.699146 | | -6.34143 | -1.65518 | -0.37801 | | -5.69469 | 1.669288 | 0.579297 | | -5.87361 | 0.679037 | 2.756602 | | -6.13628 | -0.93333 | 2.09961 | | -8.47832 | -0.37243 | 2.41709 | | -8.16826 | 1.347208 | 2.228642 | | -6.98597 | 0.517556 | -1.27517 | | | 4.08071 | 0.009763 | 0.040616 | | --- | --- | --- | | 4.137149 | -0.80172 | -1.12153 | | 3.007366 | -1.45331 | -1.60879 | | 1.808451 | -1.28621 | -0.92463 | | 1.714899 | -0.49488 | 0.247119 | | 2.86544 | 0.136956 | 0.716216 | | 0.558026 | -1.82027 | -1.19307 | | -0.3493 | -1.3786 | -0.23734 | | 0.333895 | -0.55604 | 0.685893 | | -1.7165 | -1.65601 | -0.11714 | | -2.39145 | -1.04624 | 0.953937 | | -1.73969 | -0.24234 | 1.911318 | | -0.37438 | -0.00732 | 1.758833 | | -2.51406 | 0.321505 | 3.07499 | | -2.46583 | -2.57527 | -0.96035 | | -3.79949 | -2.63535 | -0.87527 | | -4.55937 | -1.68898 | 0.027434 | | -3.7213 | -1.29295 | 1.157887 | | -5.75888 | -2.38786 | 0.666311 | | -4.95505 | -0.43067 | -0.78216 | | -5.75788 | 0.645161 | -0.02634 | | -6.05618 | 1.830094 | -0.90492 | | -5.60449 | 3.086743 | -0.79986 | | -6.01469 | 4.141892 | -1.79807 | | -4.67315 | 3.582012 | 0.277475 | | 5.295983 | -0.97588 | -1.82857 | | 5.277219 | 0.795684 | 0.526563 | | 5.665638 | 2.027315 | -0.33691 | | 7.161228 | 2.228403 | -0.0546 | | 7.662853 | 0.854845 | 0.384818 | | 6.547752 | 0.036677 | 0.519816 | | 8.806298 | 0.51612 | 0.592308 | | 3.098456 | -2.07282 | -2.49427 | | 2.823153 | 0.748655 | 1.613661 | | 0.328123 | -2.35368 | -2.01411 | | 0.136661 | 0.615416 | 2.487663 | | -3.34889 | 0.946412 | 2.744577 | | -2.94704 | -0.47138 | 3.692213 | | -1.86839 | 0.929908 | 3.711911 | | -1.93013 | -3.24573 | -1.62717 | | -4.37639 | -3.33786 | -1.46879 | | -6.50374 | -2.64004 | -0.0936 | | -5.44197 | -3.30927 | 1.158751 | | -6.23112 | -1.74925 | 1.413669 | | -4.03703 | 0.017245 | -1.17599 | | -5.52328 | -0.76636 | -1.65845 | | -6.70882 | 0.218208 | 0.314325 | | -5.21388 | 0.936437 | 0.873332 | | -6.72042 | 1.610802 | -1.74226 | | -6.5177 | 4.981403 | -1.30307 | | -5.14104 | 4.563609 | -2.30959 | | -6.6913 | 3.746561 | -2.55885 | | -3.76234 | 4.003377 | -0.16388 | | -5.13975 | 4.391547 | 0.851521 | | -4.3699 | 2.807356 | 0.981052 | | 6.051295 | -0.72583 | -1.26998 | | 5.06556 | 1.134358 | 1.549074 | | 5.048443 | 2.890587 | -0.08521 | | 5.49806 | 1.799563 | -1.39218 | | 7.740893 | 2.59362 | -0.90355 | | 7.339376 | 2.920158 | 0.776059 | | 6.64801 | -0.7937 | 1.091202 | | | 4.160753 | 0.056731 | 0.007546 | | --- | --- | --- | | 4.009242 | 0.725377 | -1.23426 | | 2.783334 | 0.767376 | -1.89267 | | 1.698926 | 0.130731 | -1.29889 | | 1.814968 | -0.56359 | -0.06897 | | 3.056875 | -0.59278 | 0.563445 | | 0.390707 | 0.01878 | -1.74246 | | -0.34834 | -0.71918 | -0.82551 | | 0.504864 | -1.1085 | 0.231044 | | -1.70104 | -1.0788 | -0.85973 | | -2.18137 | -1.82951 | 0.226621 | | -1.35775 | -2.25491 | 1.28842 | | -0.01485 | -1.88231 | 1.272948 | | -1.93491 | -3.09687 | 2.397222 | | -2.61968 | -0.8032 | -1.95412 | | -3.93198 | -1.02143 | -1.80808 | | -4.49305 | -1.48415 | -0.48119 | | -3.48119 | -2.25379 | 0.243101 | | -5.64314 | -2.47063 | -0.67592 | | -4.88772 | -0.2931 | 0.423968 | | -5.95787 | 0.656831 | -0.14724 | | -6.29643 | 1.759134 | 0.820106 | | -6.0888 | 3.076292 | 0.693767 | | -6.49596 | 4.031283 | 1.789212 | | -5.45252 | 3.739932 | -0.50159 | | 5.052602 | 1.371483 | -1.84064 | | 5.462499 | 0.083315 | 0.775361 | | 5.833455 | 1.444682 | 1.424692 | | 7.363167 | 1.397167 | 1.548483 | | 7.810447 | 0.412312 | 0.470504 | | 6.661961 | -0.1665 | -0.05513 | | 8.942154 | 0.157561 | 0.124411 | | 2.714923 | 1.281616 | -2.84505 | | 3.174882 | -1.11767 | 1.507845 | | 0.021417 | 0.47584 | -2.55863 | | 0.627969 | -2.19822 | 2.089715 | | -2.33113 | -4.04431 | 2.01976 | | -1.17418 | -3.32451 | 3.147108 | | -2.76525 | -2.59108 | 2.898703 | | -2.22579 | -0.46385 | -2.90865 | | -4.62488 | -0.8649 | -2.62841 | | -6.01742 | -2.81582 | 0.290563 | | -6.46928 | -2.00848 | -1.22077 | | -5.30331 | -3.33902 | -1.2435 | | -5.22993 | -0.70507 | 1.380249 | | -3.98301 | 0.284361 | 0.638227 | | -5.61906 | 1.062346 | -1.10304 | | -6.87305 | 0.090037 | -0.35968 | | -6.76131 | 1.415272 | 1.744995 | | -5.63285 | 4.593797 | 2.164951 | | -7.21254 | 4.774993 | 1.420116 | | -6.95432 | 3.51389 | 2.634647 | | -4.57206 | 4.318218 | -0.19782 | | -5.13641 | 3.038324 | -1.27331 | | -6.14493 | 4.454202 | -0.96301 | | 5.889118 | 1.044842 | -1.46859 | | 5.404741 | -0.67892 | 1.56312 | | 5.317018 | 1.577333 | 2.376117 | | 5.524275 | 2.259131 | 0.765541 | | 7.862095 | 2.358644 | 1.418967 | | 7.690749 | 0.993801 | 2.512971 | | 6.767506 | -1.04613 | -0.54614 | |

**Table S10 Coordinates of (5ʹʹ*R*)-3**

|  | 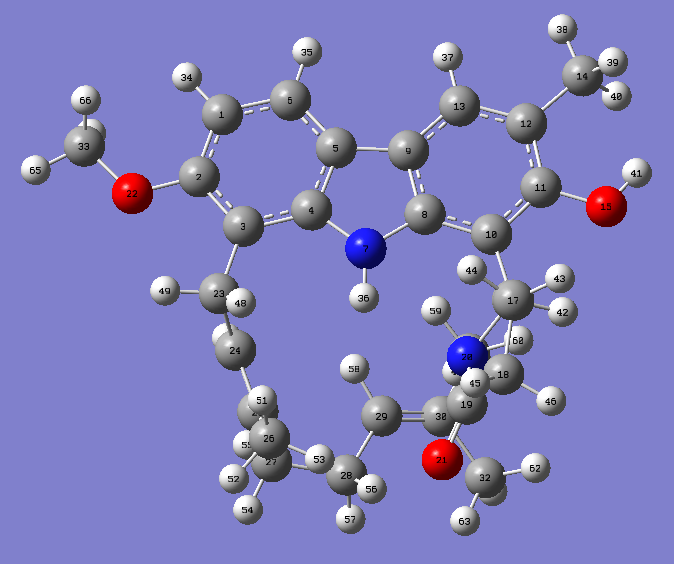  Conformer (5ʹʹ*R*)-**3** (1) | 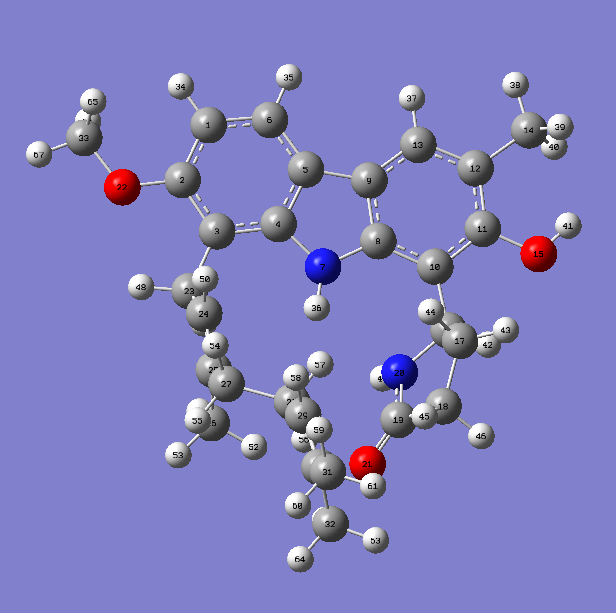  Conformer (5ʹʹ*R*)-**3** (16) | 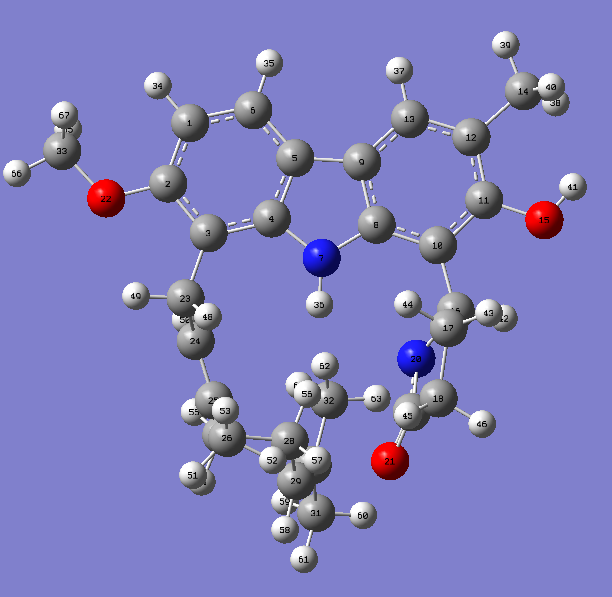  **Conformer (5ʹʹ*R*)-3 (23)** |
| --- | --- | --- | --- |
| | 1 | | --- | | 2 | | 3 | | 4 | | 5 | | 6 | | 7 | | 8 | | 9 | | 10 | | 11 | | 12 | | 13 | | 14 | | 15 | | 16 | | 17 | | 18 | | 19 | | 20 | | 21 | | 22 | | 23 | | 24 | | 25 | | 26 | | 27 | | 28 | | 29 | | 30 | | 31 | | 32 | | 33 | | 34 | | 35 | | 36 | | 37 | | 38 | | 39 | | 40 | | 41 | | 42 | | 43 | | 44 | | 45 | | 46 | | 47 | | 48 | | 49 | | 50 | | 51 | | 52 | | 53 | | 54 | | 55 | | 56 | | 57 | | 58 | | 59 | | 60 | | 61 | | 62 | | 63 | | 64 | | 65 | | 66 | | 67 | | | 4.056565 | 2.200699 | 0.409343 | | --- | --- | --- | | 4.07833 | 0.891671 | -0.10739 | | 2.903211 | 0.234148 | -0.50769 | | 1.716135 | 0.953567 | -0.34835 | | 1.664881 | 2.26796 | 0.178112 | | 2.855134 | 2.88733 | 0.551729 | | 0.421471 | 0.557911 | -0.6657 | | -0.47062 | 1.56314 | -0.315 | | 0.269628 | 2.656251 | 0.201264 | | -1.86856 | 1.592725 | -0.41684 | | -2.49316 | 2.770638 | 0.013891 | | -1.78963 | 3.880532 | 0.528364 | | -0.40219 | 3.805101 | 0.614145 | | -2.53515 | 5.118013 | 0.969005 | | -3.86714 | 2.807968 | -0.10916 | | -2.71262 | 0.483751 | -1.00105 | | -2.85268 | 0.527677 | -2.5468 | | -3.14159 | -0.92885 | -2.93009 | | -2.53693 | -1.7498 | -1.79368 | | -2.18103 | -0.87443 | -0.79618 | | -2.4011 | -2.95964 | -1.75909 | | 5.238161 | 0.171991 | -0.25475 | | 2.912538 | -1.1772 | -1.05781 | | 2.33485 | -2.18105 | -0.08257 | | 1.51519 | -3.21178 | -0.33477 | | 1.029602 | -3.60306 | -1.70884 | | 1.038303 | -4.1059 | 0.793345 | | -0.44507 | -3.90991 | 1.188614 | | -0.70154 | -2.59422 | 1.888604 | | -1.78713 | -2.25229 | 2.598779 | | -1.88319 | -0.91624 | 3.294582 | | -2.97904 | -3.15648 | 2.789221 | | 6.471738 | 0.769006 | 0.105707 | | 4.977127 | 2.686585 | 0.704898 | | 2.858596 | 3.89516 | 0.955152 | | 0.139951 | -0.38355 | -0.88718 | | 0.152438 | 4.651644 | 1.008832 | | -1.841 | 5.872156 | 1.342981 | | -3.09632 | 5.586299 | 0.150279 | | -3.24459 | 4.918147 | 1.782942 | | -4.21336 | 3.622931 | 0.27043 | | -3.7102 | 0.551687 | -0.55622 | | -3.62806 | 1.231152 | -2.8511 | | -1.90815 | 0.860816 | -2.98499 | | -2.72901 | -1.23976 | -3.89102 | | -4.21517 | -1.14693 | -2.95576 | | -2.00581 | -1.24408 | 0.134205 | | 2.369259 | -1.19763 | -2.00791 | | 3.944526 | -1.44919 | -1.29308 | | 2.655393 | -2.02964 | 0.947711 | | 1.332749 | -2.9035 | -2.48911 | | 1.437207 | -4.58438 | -1.98158 | | -0.0592 | -3.69555 | -1.74636 | | 1.172477 | -5.15358 | 0.496053 | | 1.665729 | -3.95326 | 1.677885 | | -1.07718 | -3.99694 | 0.297237 | | -0.73491 | -4.74196 | 1.840056 | | 0.099337 | -1.8594 | 1.814569 | | -1.00197 | -0.29618 | 3.117786 | | -2.76548 | -0.35251 | 2.967318 | | -1.99021 | -1.04747 | 4.378399 | | -3.90252 | -2.65964 | 2.46859 | | -2.89922 | -4.09147 | 2.234848 | | -3.11667 | -3.40145 | 3.84971 | | 7.235485 | 0.021639 | -0.10413 | | 6.673781 | 1.666952 | -0.48792 | | 6.500601 | 1.023384 | 1.170613 | | | -4.02098 | 2.842595 | -0.65634 | | --- | --- | --- | | -2.82086 | 3.325056 | -0.10043 | | -1.79827 | 2.458213 | 0.320293 | | -2.04025 | 1.094742 | 0.132328 | | -3.24268 | 0.58203 | -0.41338 | | -4.23516 | 1.47662 | -0.8066 | | -1.21645 | 0.024098 | 0.457011 | | -1.84484 | -1.16891 | 0.123733 | | -3.1211 | -0.86098 | -0.41406 | | -1.40065 | -2.49177 | 0.265548 | | -2.28742 | -3.49025 | -0.15878 | | -3.56429 | -3.2216 | -0.69429 | | -3.96588 | -1.89406 | -0.81395 | | -4.46265 | -4.35448 | -1.13116 | | -1.83426 | -4.78809 | -0.03899 | | -0.04608 | -2.90223 | 0.796304 | | 1.062452 | -3.0364 | -0.28277 | | 2.363489 | -2.79627 | 0.493537 | | 1.944293 | -1.93901 | 1.684613 | | 0.569078 | -1.93666 | 1.720203 | | 2.671185 | -1.36625 | 2.474421 | | -2.57358 | 4.6638 | 0.075992 | | -0.52673 | 2.962065 | 0.970946 | | 0.719291 | 2.71965 | 0.145253 | | 1.949294 | 2.411958 | 0.58192 | | 2.323328 | 2.246124 | 2.035262 | | 3.085881 | 2.234678 | -0.40493 | | 3.537948 | 0.76456 | -0.58172 | | 4.662831 | 0.62522 | -1.56951 | | 5.914853 | 0.198399 | -1.3547 | | 6.917114 | 0.143587 | -2.48208 | | 6.455591 | -0.25629 | -0.02218 | | -3.56428 | 5.604877 | -0.2995 | | -4.79394 | 3.532356 | -0.96848 | | -5.17085 | 1.124061 | -1.22921 | | -0.25792 | 0.100173 | 0.755351 | | -4.94557 | -1.67056 | -1.22654 | | -5.40781 | -3.96858 | -1.51614 | | -4.01982 | -4.95818 | -1.93362 | | -4.71749 | -5.03538 | -0.30833 | | -2.5265 | -5.4076 | -0.29432 | | -0.16113 | -3.86661 | 1.301868 | | 1.010951 | -4.00533 | -0.77911 | | 0.92082 | -2.26321 | -1.04278 | | 3.153055 | -2.30814 | -0.07839 | | 2.782201 | -3.72625 | 0.894916 | | 0.109388 | -1.6652 | 2.579231 | | -0.63737 | 4.034257 | 1.151184 | | -0.43035 | 2.499331 | 1.959068 | | 0.579551 | 2.850117 | -0.92752 | | 1.495126 | 2.441796 | 2.716863 | | 2.698268 | 1.240842 | 2.251355 | | 3.128301 | 2.943294 | 2.296696 | | 2.792883 | 2.635168 | -1.38118 | | 3.951718 | 2.823265 | -0.07592 | | 3.803156 | 0.338807 | 0.38704 | | 2.671668 | 0.193651 | -0.94271 | | 4.410383 | 0.932582 | -2.58573 | | 6.488917 | 0.48016 | -3.42881 | | 7.791334 | 0.769499 | -2.2654 | | 7.296344 | -0.8755 | -2.62627 | | 5.722381 | -0.22591 | 0.783185 | | 6.833861 | -1.28359 | -0.08796 | | 7.308619 | 0.363735 | 0.279012 | | -3.78 | 5.556609 | -1.37216 | | -4.49182 | 5.460699 | 0.264755 | | -3.14895 | 6.583137 | -0.06203 | | | -2.14536 | 4.04954 | 0.745869 | | --- | --- | --- | | -1.02598 | 3.964483 | -0.10291 | | -0.57622 | 2.735664 | -0.61438 | | -1.29572 | 1.608072 | -0.21333 | | -2.42347 | 1.664462 | 0.642041 | | -2.84424 | 2.904911 | 1.115552 | | -1.08241 | 0.280331 | -0.57255 | | -2.01641 | -0.52628 | 0.065916 | | -2.8829 | 0.302057 | 0.82059 | | -2.16957 | -1.91926 | 0.019302 | | -3.23326 | -2.44943 | 0.761101 | | -4.11733 | -1.65576 | 1.523412 | | -3.92698 | -0.27667 | 1.539731 | | -5.2454 | -2.29864 | 2.295013 | | -3.39947 | -3.81717 | 0.695392 | | -1.30608 | -2.83712 | -0.81287 | | -1.66403 | -2.87701 | -2.32326 | | -0.34813 | -3.27606 | -3.00456 | | 0.740325 | -2.82519 | -2.03306 | | 0.114771 | -2.44809 | -0.86659 | | 1.940098 | -2.80542 | -2.23066 | | -0.30253 | 5.064947 | -0.48938 | | 0.62122 | 2.634199 | -1.53606 | | 1.854427 | 2.101278 | -0.83773 | | 2.766162 | 1.235261 | -1.302 | | 2.745395 | 0.619831 | -2.67944 | | 3.936922 | 0.826265 | -0.4299 | | 3.760941 | -0.56273 | 0.234351 | | 4.996917 | -1.00494 | 0.9673 | | 5.180671 | -1.16643 | 2.284474 | | 6.51277 | -1.61892 | 2.831661 | | 4.126469 | -0.93257 | 3.337556 | | -0.71527 | 6.349526 | -0.05597 | | -2.47384 | 5.009555 | 1.12185 | | -3.70543 | 2.99094 | 1.770822 | | -0.23299 | -0.07441 | -0.98223 | | -4.60211 | 0.345283 | 2.120574 | | -4.88976 | -3.01075 | 3.051445 | | -5.82393 | -1.54418 | 2.830275 | | -5.95027 | -2.83257 | 1.645055 | | -4.10327 | -4.09608 | 1.291267 | | -1.38918 | -3.84726 | -0.40239 | | -2.48856 | -3.56499 | -2.51234 | | -1.97909 | -1.88182 | -2.64737 | | -0.19125 | -2.83529 | -3.99013 | | -0.25256 | -4.36155 | -3.11924 | | 0.670219 | -2.38395 | -0.02343 | | 0.35831 | 2.011578 | -2.39744 | | 0.83267 | 3.629599 | -1.93546 | | 2.006726 | 2.50632 | 0.162395 | | 3.602041 | 0.980372 | -3.26215 | | 2.831478 | -0.46975 | -2.63902 | | 1.846965 | 0.863197 | -3.24893 | | 4.853496 | 0.812095 | -1.03395 | | 4.097978 | 1.570387 | 0.356232 | | 2.894388 | -0.51786 | 0.899946 | | 3.520957 | -1.30399 | -0.53655 | | 5.851435 | -1.20558 | 0.319399 | | 6.92525 | -0.88248 | 3.532237 | | 6.413609 | -2.55641 | 3.392675 | | 7.24751 | -1.77834 | 2.039528 | | 3.178157 | -0.57922 | 2.932723 | | 3.927516 | -1.85282 | 3.900427 | | 4.469616 | -0.19313 | 4.071008 | | -0.67703 | 6.438562 | 1.034964 | | -0.00963 | 7.052163 | -0.49667 | | -1.726 | 6.586611 | -0.40485 | |

|  | 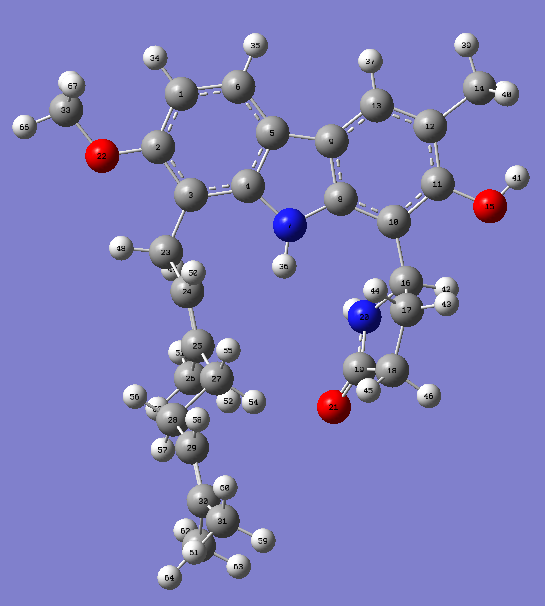  Conformer (5ʹʹ*R*)-**3** (25) | 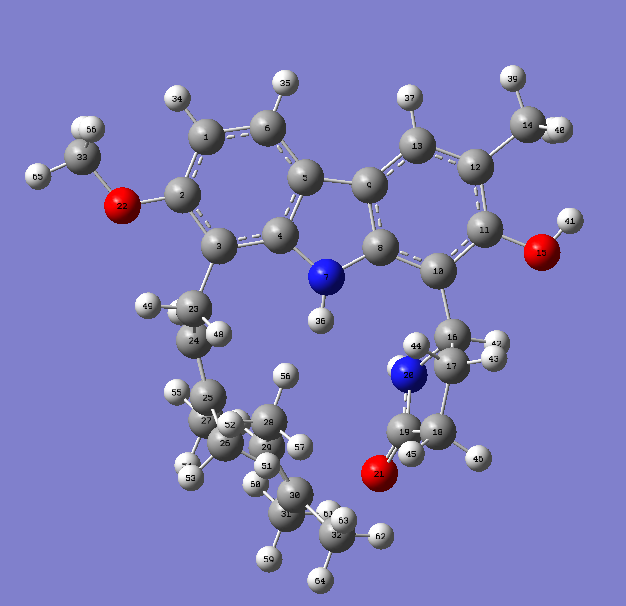  Conformer (5ʹʹ*R*)-**3** (28) | 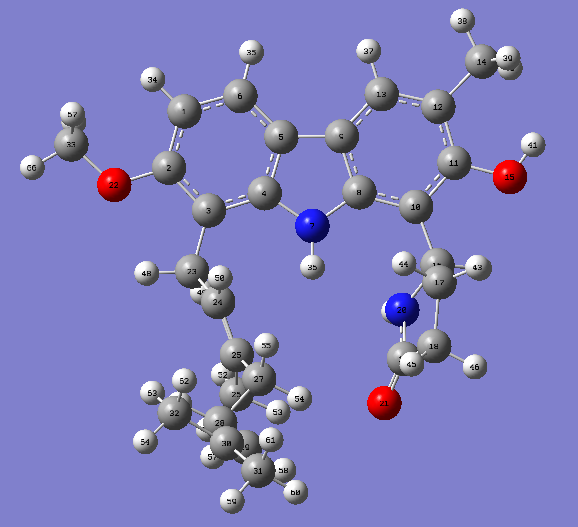  **Conformer (5ʹʹ*R*)-3 (30)** |
| --- | --- | --- | --- |
| | 1 | | --- | | 2 | | 3 | | 4 | | 5 | | 6 | | 7 | | 8 | | 9 | | 10 | | 11 | | 12 | | 13 | | 14 | | 15 | | 16 | | 17 | | 18 | | 19 | | 20 | | 21 | | 22 | | 23 | | 24 | | 25 | | 26 | | 27 | | 28 | | 29 | | 30 | | 31 | | 32 | | 33 | | 34 | | 35 | | 36 | | 37 | | 38 | | 39 | | 40 | | 41 | | 42 | | 43 | | 44 | | 45 | | 46 | | 47 | | 48 | | 49 | | 50 | | 51 | | 52 | | 53 | | 54 | | 55 | | 56 | | 57 | | 58 | | 59 | | 60 | | 61 | | 62 | | 63 | | 64 | | 65 | | 66 | | 67 | | | -3.10721 | 3.782252 | -0.494 | | --- | --- | --- | | -1.79147 | 3.826477 | 0.004325 | | -1.08324 | 2.658216 | 0.334273 | | -1.7668 | 1.455629 | 0.131426 | | -3.0945 | 1.383119 | -0.35976 | | -3.75924 | 2.56658 | -0.67125 | | -1.3225 | 0.162588 | 0.376089 | | -2.31632 | -0.74582 | 0.044619 | | -3.44676 | -0.02082 | -0.41048 | | -2.30863 | -2.14689 | 0.112499 | | -3.48439 | -2.79141 | -0.29278 | | -4.63195 | -2.10318 | -0.74178 | | -4.59358 | -0.71258 | -0.79557 | | -5.86411 | -2.86948 | -1.16124 | | -3.46586 | -4.17029 | -0.25017 | | -1.11847 | -2.97374 | 0.539042 | | -0.0728 | -3.24042 | -0.57793 | | 1.239473 | -3.45658 | 0.187728 | | 1.045536 | -2.70241 | 1.50169 | | -0.27866 | -2.34054 | 1.568934 | | 1.887992 | -2.46549 | 2.346553 | | -1.11883 | 5.005783 | 0.209525 | | 0.316118 | 2.702603 | 0.915226 | | 1.324419 | 1.8646 | 0.160616 | | 2.340852 | 1.145438 | 0.660777 | | 2.653982 | 1.011328 | 2.130904 | | 3.28398 | 0.413963 | -0.27327 | | 4.708187 | 1.019677 | -0.33656 | | 5.567116 | 0.337536 | -1.36519 | | 6.668356 | -0.40305 | -1.18174 | | 7.390821 | -1.01927 | -2.35508 | | 7.296721 | -0.69964 | 0.156517 | | -1.75652 | 6.230205 | -0.11009 | | -3.62863 | 4.697973 | -0.7397 | | -4.77673 | 2.554168 | -1.04943 | | -0.39162 | -0.08248 | 0.672173 | | -5.46768 | -0.17076 | -1.14544 | | -6.28492 | -3.4737 | -0.34668 | | -6.65322 | -2.18546 | -1.47722 | | -5.67458 | -3.54264 | -2.00708 | | -4.33366 | -4.52222 | -0.47659 | | -1.48929 | -3.93316 | 0.911777 | | -0.36986 | -4.08477 | -1.20042 | | 0.000164 | -2.36013 | -1.22198 | | 2.132732 | -3.10548 | -0.33047 | | 1.405301 | -4.51048 | 0.437793 | | -0.6614 | -2.06128 | 2.46237 | | 0.647734 | 3.743781 | 0.92344 | | 0.273484 | 2.399327 | 1.967403 | | 1.210074 | 1.891747 | -0.92309 | | 1.907481 | 1.479391 | 2.773359 | | 2.732266 | -0.04274 | 2.415821 | | 3.616352 | 1.477801 | 2.369966 | | 3.374784 | -0.63286 | 0.042547 | | 2.863003 | 0.403508 | -1.28451 | | 4.615432 | 2.082949 | -0.59359 | | 5.17046 | 0.983055 | 0.651701 | | 5.219795 | 0.463255 | -2.39197 | | 7.43625 | -2.11131 | -2.26342 | | 6.908069 | -0.78069 | -3.3053 | | 8.429549 | -0.67097 | -2.40815 | | 6.759621 | -0.26178 | 0.997395 | | 7.351558 | -1.78143 | 0.326316 | | 8.328867 | -0.3305 | 0.190734 | | -2.65983 | 6.38283 | 0.490067 | | -1.03457 | 7.010731 | 0.125494 | | -2.01287 | 6.285994 | -1.17344 | | | 3.572487 | 3.187131 | -0.67881 | | --- | --- | --- | | 2.406336 | 3.511095 | 0.039292 | | 1.519598 | 2.521768 | 0.496799 | | 1.856315 | 1.204931 | 0.176279 | | 3.021948 | 0.851343 | -0.5466 | | 3.882028 | 1.862248 | -0.96859 | | 1.172995 | 0.037573 | 0.50802 | | 1.84512 | -1.05633 | -0.0245 | | 3.014412 | -0.59168 | -0.6748 | | 1.516312 | -2.4182 | 0.034244 | | 2.413859 | -3.29831 | -0.58489 | | 3.589352 | -2.87198 | -1.23991 | | 3.874931 | -1.50989 | -1.27352 | | 4.512651 | -3.8812 | -1.88051 | | 2.104175 | -4.6402 | -0.50712 | | 0.307634 | -2.96523 | 0.754665 | | 0.439212 | -3.03681 | 2.299764 | | -1.01144 | -2.96635 | 2.795867 | | -1.76416 | -2.24413 | 1.680973 | | -0.90699 | -2.14104 | 0.611253 | | -2.91101 | -1.83747 | 1.71307 | | 2.057217 | 4.802895 | 0.343781 | | 0.269457 | 2.859569 | 1.282199 | | -0.99306 | 2.726496 | 0.457548 | | -2.17671 | 2.215585 | 0.824013 | | -2.49445 | 1.681255 | 2.198947 | | -3.32018 | 2.154278 | -0.17007 | | -3.48359 | 0.762147 | -0.82964 | | -4.65195 | 0.709776 | -1.77447 | | -5.72597 | -0.09026 | -1.72896 | | -6.81366 | 0.019541 | -2.77027 | | -5.97125 | -1.14964 | -0.68364 | | 2.91503 | 5.860741 | -0.04782 | | 4.242058 | 3.967985 | -1.01496 | | 4.786425 | 1.632266 | -1.52315 | | 0.210325 | 0.004293 | 0.804288 | | 4.777296 | -1.16896 | -1.77303 | | 4.023019 | -4.45876 | -2.67587 | | 5.36607 | -3.38267 | -2.34259 | | 4.921138 | -4.59769 | -1.15666 | | 2.728294 | -5.15643 | -1.02881 | | 0.109304 | -3.96907 | 0.369521 | | 0.969973 | -3.93821 | 2.60748 | | 1.009576 | -2.17508 | 2.655635 | | -1.14496 | -2.44987 | 3.747537 | | -1.46192 | -3.95938 | 2.903368 | | -1.29371 | -1.93398 | -0.30045 | | 0.223721 | 2.223995 | 2.172354 | | 0.359705 | 3.885969 | 1.647129 | | -0.90397 | 3.117734 | -0.55571 | | -2.92463 | 0.676651 | 2.152834 | | -1.62838 | 1.646442 | 2.862011 | | -3.24352 | 2.319328 | 2.683599 | | -4.26028 | 2.413772 | 0.331687 | | -3.17126 | 2.903084 | -0.95547 | | -2.55911 | 0.554773 | -1.38716 | | -3.55916 | -0.00666 | -0.05959 | | -4.60593 | 1.434257 | -2.58964 | | -7.78294 | 0.242916 | -2.3079 | | -6.60245 | 0.801169 | -3.50346 | | -6.94162 | -0.92589 | -3.31166 | | -6.10108 | -2.13146 | -1.15554 | | -5.17222 | -1.24016 | 0.051113 | | -6.90398 | -0.94756 | -0.14273 | | 2.443667 | 6.773745 | 0.312836 | | 3.907192 | 5.763665 | 0.405705 | | 3.016618 | 5.914921 | -1.13696 | | | -2.3789 | 4.104917 | -0.5232 | | --- | --- | --- | | -1.11995 | 3.935293 | 0.082957 | | -0.63513 | 2.66701 | 0.447025 | | -1.48035 | 1.589673 | 0.163979 | | -2.75735 | 1.734055 | -0.43447 | | -3.19792 | 3.009962 | -0.77682 | | -1.26883 | 0.240033 | 0.416533 | | -2.36297 | -0.49536 | -0.01421 | | -3.32292 | 0.405225 | -0.54196 | | -2.58427 | -1.88025 | 0.021888 | | -3.81187 | -2.32312 | -0.48699 | | -4.7951 | -1.45575 | -1.00941 | | -4.5307 | -0.08923 | -1.03039 | | -6.09606 | -2.0106 | -1.53947 | | -4.01728 | -3.68745 | -0.47273 | | -1.5786 | -2.89531 | 0.512951 | | -0.52569 | -3.33018 | -0.54276 | | 0.685249 | -3.75947 | 0.295975 | | 0.539619 | -2.98489 | 1.604156 | | -0.71095 | -2.41505 | 1.600228 | | 1.360062 | -2.88654 | 2.497127 | | -0.28982 | 4.991483 | 0.367676 | | 0.697256 | 2.490964 | 1.148781 | | 1.608838 | 1.467417 | 0.509774 | | 2.428913 | 0.59758 | 1.119516 | | 2.565363 | 0.451492 | 2.615191 | | 3.316815 | -0.31302 | 0.295224 | | 4.819718 | 0.0587 | 0.344975 | | 5.665031 | -0.90237 | -0.44358 | | 6.406529 | -0.66895 | -1.53466 | | 7.186395 | -1.78118 | -2.19302 | | 6.553397 | 0.672114 | -2.20869 | | -0.69558 | 6.303726 | 0.019626 | | -2.72596 | 5.093663 | -0.79268 | | -4.16874 | 3.161905 | -1.23816 | | -0.41221 | -0.15126 | 0.774202 | | -5.27581 | 0.589622 | -1.43529 | | -6.73815 | -1.20629 | -1.90184 | | -5.94913 | -2.69843 | -2.38199 | | -6.67174 | -2.54608 | -0.77298 | | -4.90893 | -3.89277 | -0.77435 | | -2.12708 | -3.78012 | 0.849673 | | -0.92 | -4.11406 | -1.18976 | | -0.27138 | -2.47408 | -1.17319 | | 1.653197 | -3.55974 | -0.16556 | | 0.660523 | -4.82647 | 0.544385 | | -1.09666 | -2.08076 | 2.473233 | | 1.201177 | 3.460495 | 1.163872 | | 0.513206 | 2.240621 | 2.199685 | | 1.609073 | 1.482321 | -0.58028 | | 3.567209 | 0.744944 | 2.947928 | | 1.854945 | 1.062401 | 3.173058 | | 2.425509 | -0.59237 | 2.913641 | | 3.204363 | -1.34481 | 0.652023 | | 2.993454 | -0.30074 | -0.75056 | | 4.94241 | 1.087182 | -0.00303 | | 5.156226 | 0.045623 | 1.388832 | | 5.648195 | -1.9252 | -0.06422 | | 8.25932 | -1.55412 | -2.21474 | | 7.055953 | -2.73465 | -1.67666 | | 6.879085 | -1.91715 | -3.23717 | | 6.240962 | 0.613899 | -3.25823 | | 5.974237 | 1.463954 | -1.7343 | | 7.603535 | 0.988291 | -2.21869 | | -1.61144 | 6.595154 | 0.544768 | | 0.118076 | 6.957585 | 0.330155 | | -0.84744 | 6.406 | -1.0602 | |

**The NMR data for (*R*)-MPA ester (8r).** ^1^H NMR (CDCl_3_, 500 MHz): *δ* 1.58 (3H, s, H-9), 1.68 (3H, s, H-10′), 1.99 (3H, s, 3-CH_3_), 2.10 (4H, m, H-5′, H-6′), 2.88 (1H, dd, *J* = 14.1, 6.4 Hz, H-1′a), 3.34 (1H, dd, *J* = 14.1, 6.4 Hz, H-1′b), 3.82 (3H, s, -OCH_3_), 4.66 (1H, s, H-4′a), 5.08 (1H, m, H-2′), 5.10 (1H, s, H-4′b), 5.39 (1H, t, *J* = 6.3 Hz, H-7′), 6.74 (1H, d, *J* = 8.5 Hz, H-6), 7.06 (1H, s, H-1), 7.68 (1H, s, H-4), 7.74 (1H, d, *J* = 8.5 Hz, H-5), 8.98 (1H, br s, H-9).

**The NMR data for (*S*)-MPA ester (8s).** ^1^H NMR (CDCl_3_, 500 MHz): *δ* 1.56 (3H, s, H-9), 1.66 (3H, s, H-10′), 2.03 (3H, s, 3-CH_3_), 2.06 (4H, m, H-5′, H-6′), 2.96 (1H, dd, *J* = 14.1, 6.4 Hz, H-1′a), 3.49 (1H, m, H-1′b), 3.86 (3H, s, -OCH_3_), 4.59 (1H, s, H-4′a), 5.03 (1H, m, H-2′), 5.10 (1H, s, H-4′b), 5.36 (1H, t, *J* = 6.6 Hz, H-7′), 6.77 (1H, d, *J* = 8.5 Hz, H-6), 7.07 (1H, s, H-1), 7.70 (1H, s, H-4), 7.76 (1H, d, *J* = 8.5 Hz, H-5), 8.85 (1H, br s, H-9).

**Determinations of ECD data of the *in situ* formed transition metal complexes** **of 4 and 9.**

A 1:2 mixture of the secondary alcohol–Rh_2_(OCOCF_3_)_4_ for **4** was subjected to ECD measurement at a concentration of 0.1 mg/mL in anhydrous CH_2_Cl_2_. The first ECD spectrum was recorded immediately after mixing, and its time evolution was monitored until stationary (about 10 min after mixing). The initial ECD was subtracted, and the observed sign of the E band at about 350 nm in the induced ECD spectrum was correlated to the absolute configuration of the secondary alcohol.

An 1:2 mixture of **9** and Mo_2_(OAc)_4_ was subjected to ECD measurement at a concentration of 0.6 mg/mL anhydrous DMSO. The initial ECD spectrum was recorded immediately after mixing. About 30 min after mixing, its time evolution was monitored until stationary, and the inherent ECD was subtracted. The observed sign of the E band at ca. 310 nm in the induced ECD spectrum was used to define the absolute configuration of the vicinal diol unit.

**Figure S1**. The key HMBC correlations of compounds **8**–**15**.


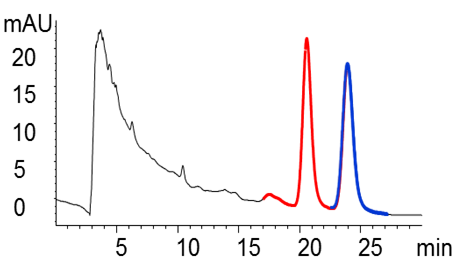
 **
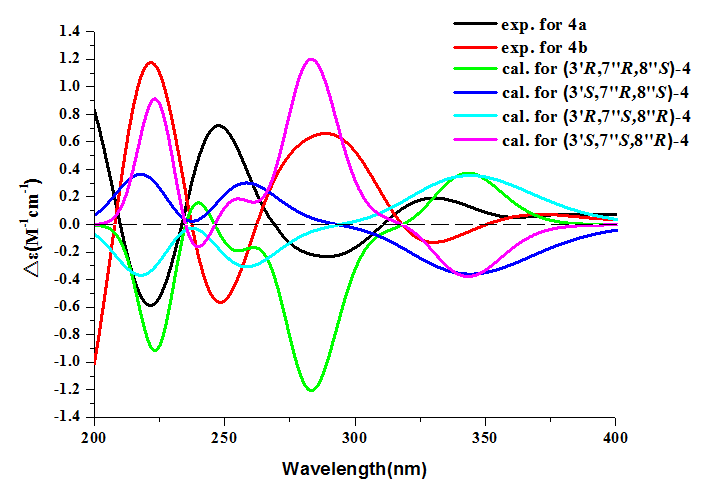
**

**4a**

**4b**

**Figure S2**. The chiral HPLC separation and experimental and calculated ECD data of compounds **4a** and **4b**.


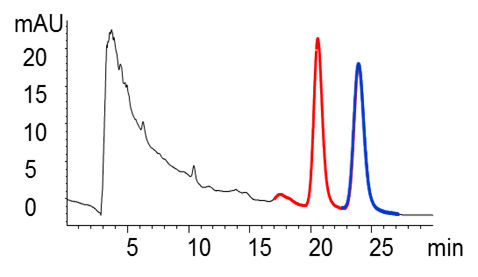

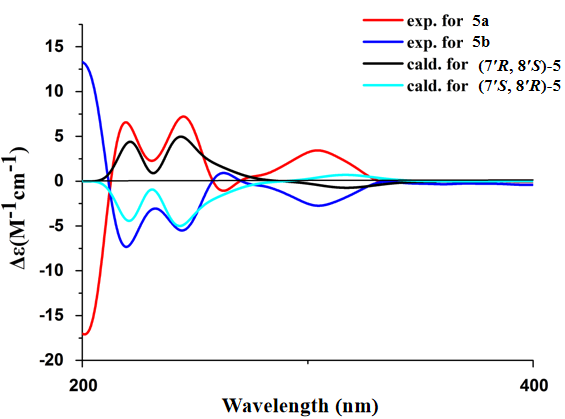


**5b**

**5a**

**Figure S3.** The chiral HPLC separation and experimental and calculated ECD data of compounds **5a** and **5b**.


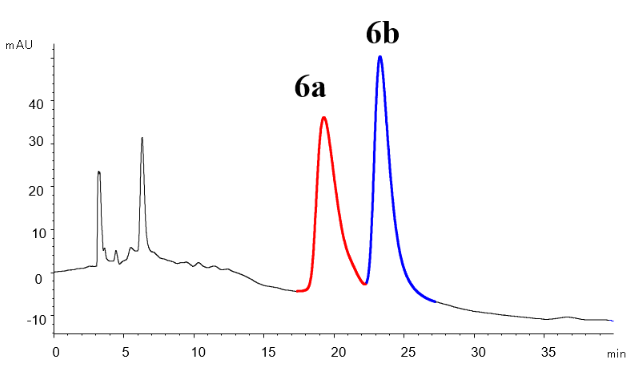


**Figure S4.** The chiral HPLC analysis of compound **6**


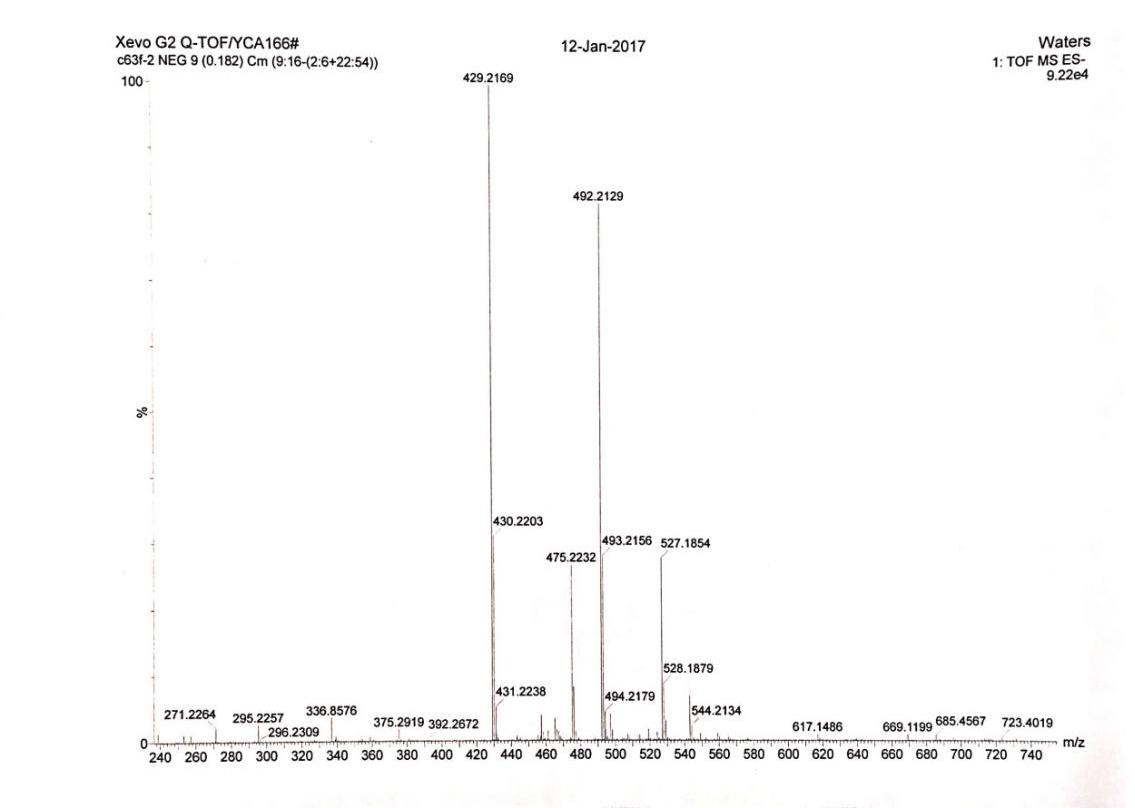


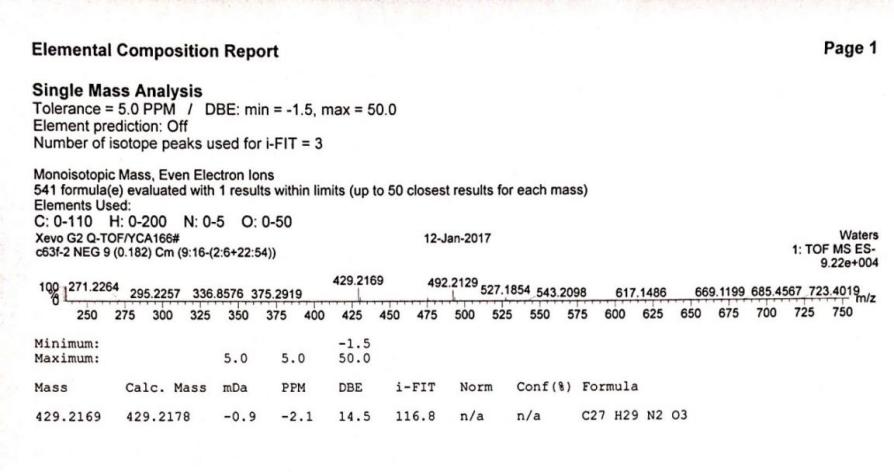


**Figure S5.** HRESIMS spectrum for **1**.


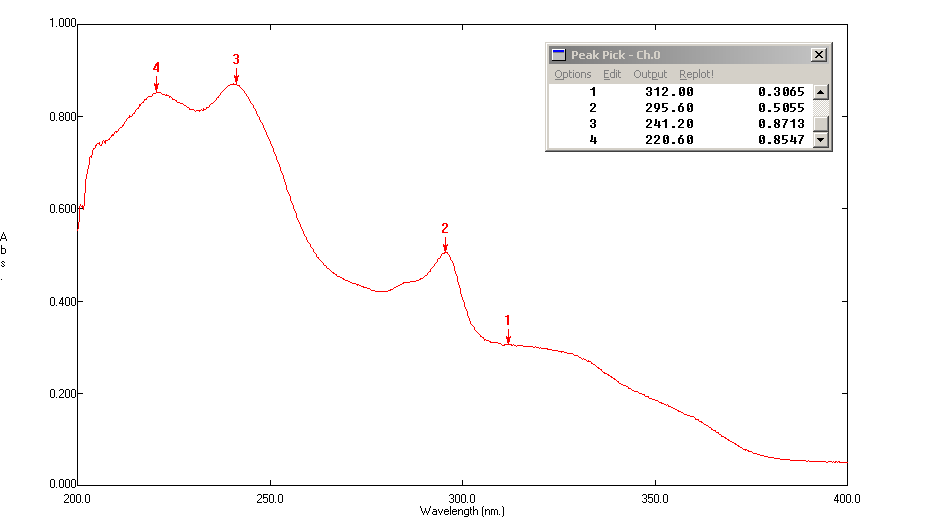


**Figure S6.** UV spectrum for **1**.


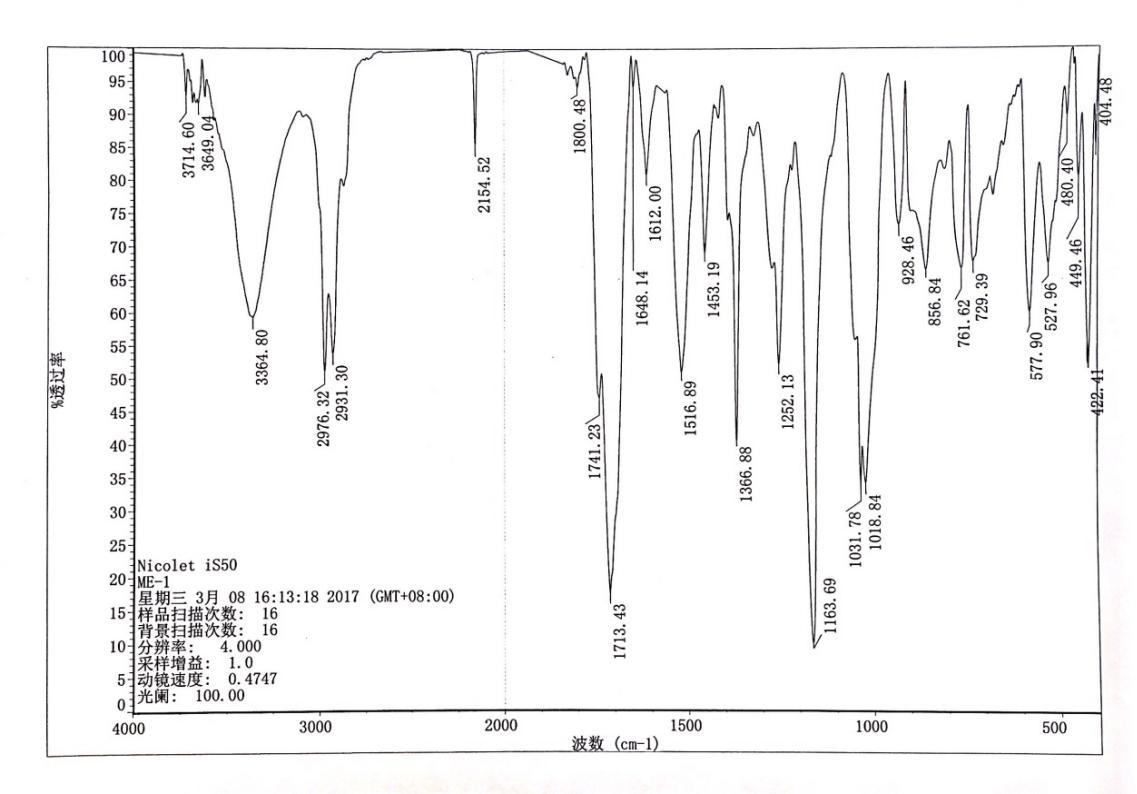


**Figure S7.** IR spectrum for **1**.


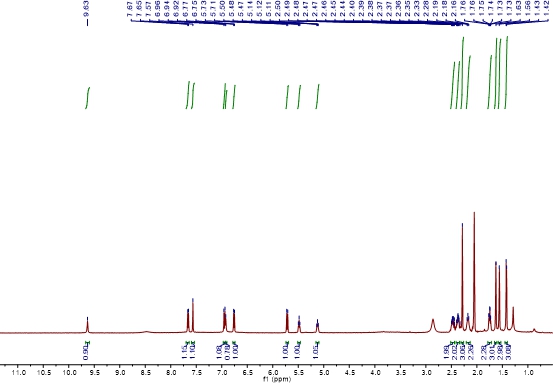


**Figure S8.** ^1^H NMR spectrum for **1** in acetone-*d*_6_.


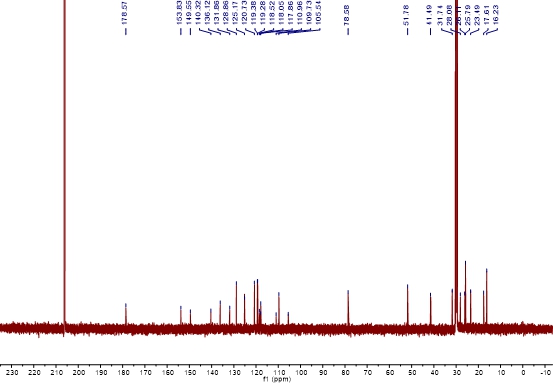


**Figure S9.** ^13^C NMR spectrum for **1** in acetone-*d*_6_.


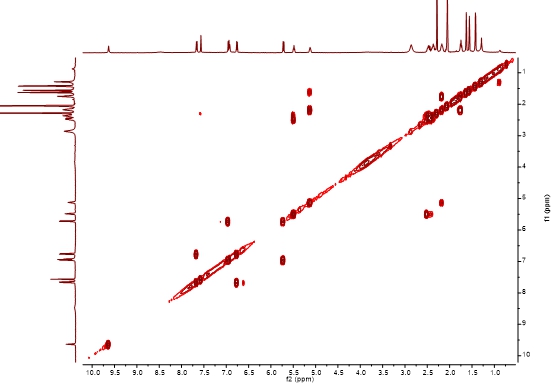


**Figure S10.** ^1^H-^1^H gCOSY spectrum for **1** in acetone-*d*_6_.


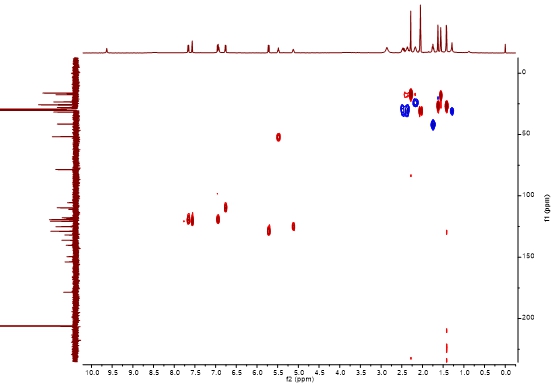


**Figure S11.** HSQC spectrum for **1** in acetone-*d*_6_.


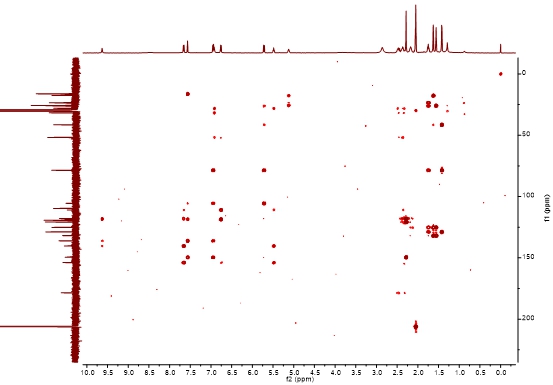


**Figure S12.** HMBC spectrum for **1** in acetone-*d*_6_.


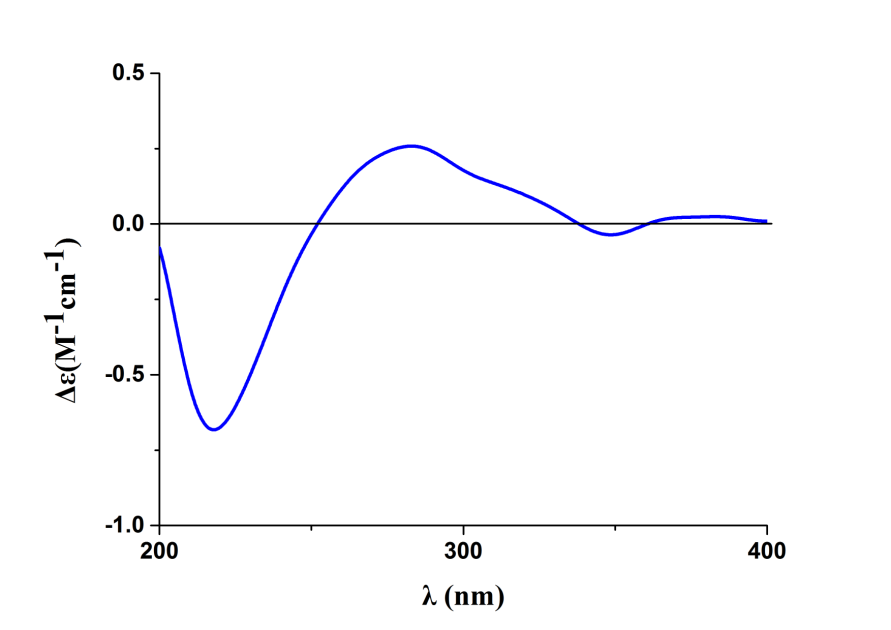

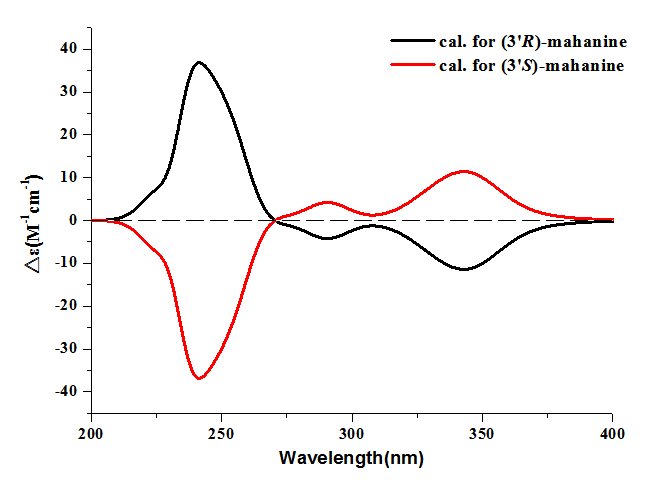


**Figure S13**. ECD spectrum for **1** and its precursor of mahanine in MeOH.


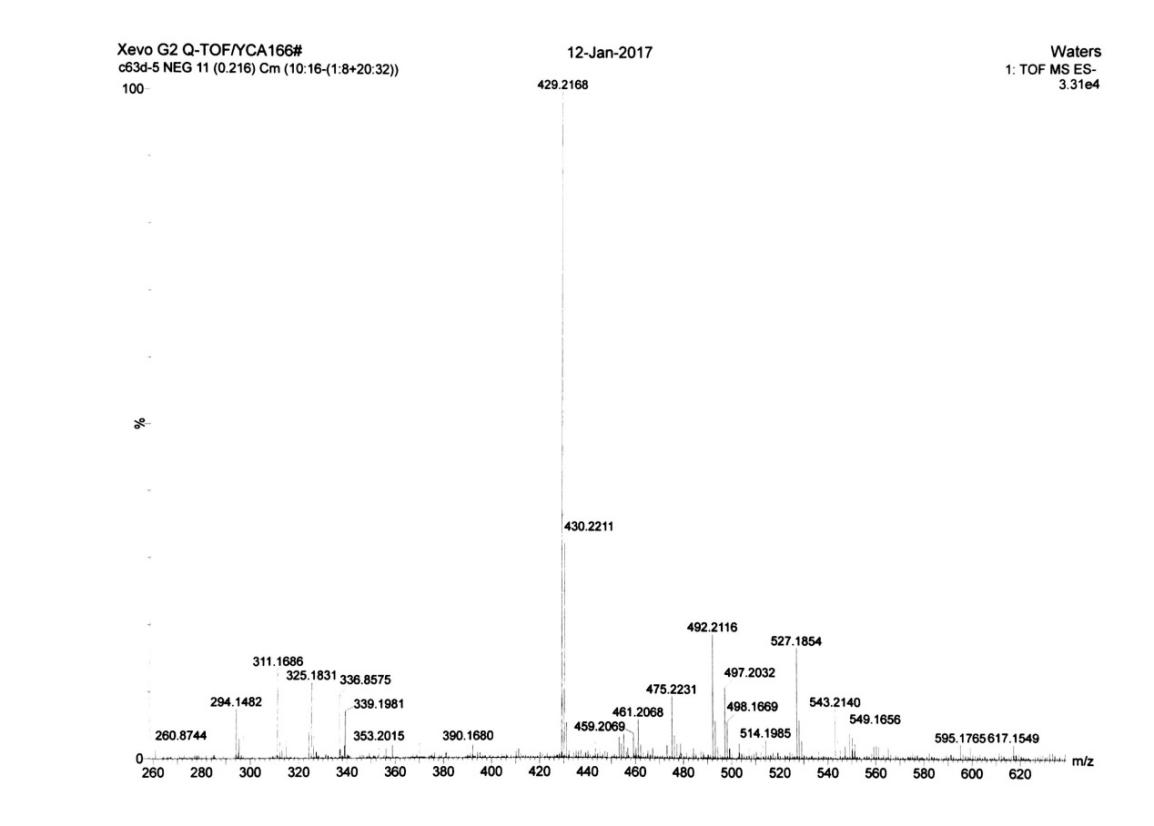


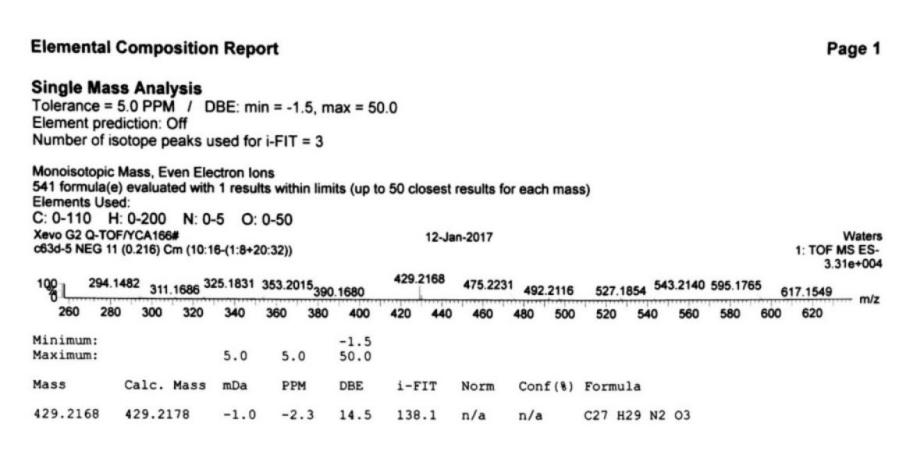


**Figure S14.** HRESIMS spectrum for **2**.


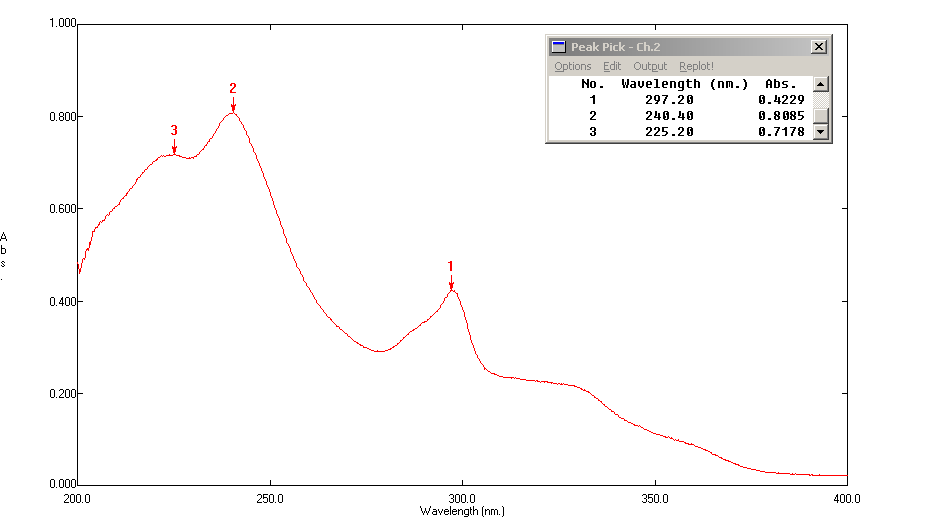


**Figure S15.** UV spectrum for **2**.


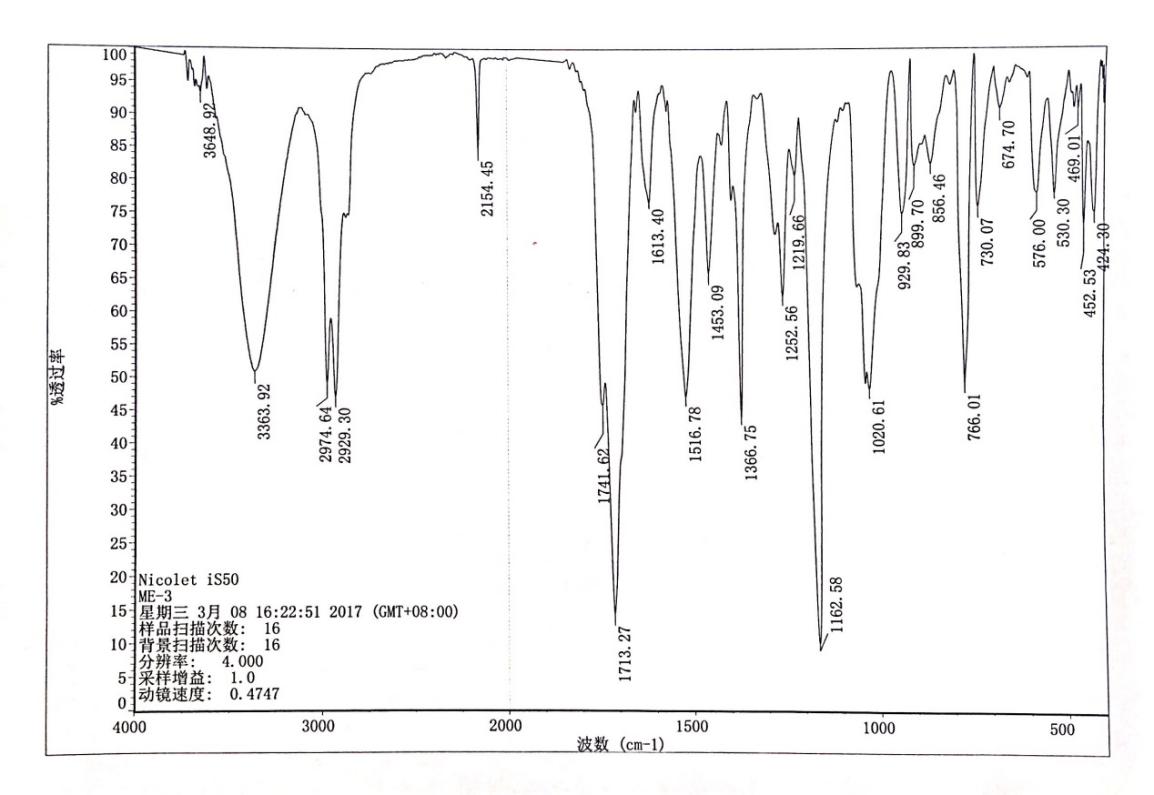


**Figure S16.** IR spectrum for **2**.


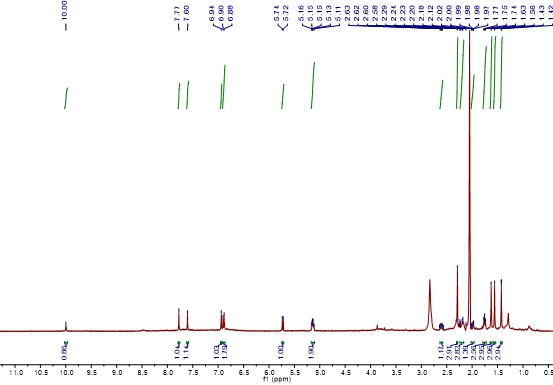


**Figure S17.** ^1^H NMR spectrum for **2** in acetone-*d*_6_.


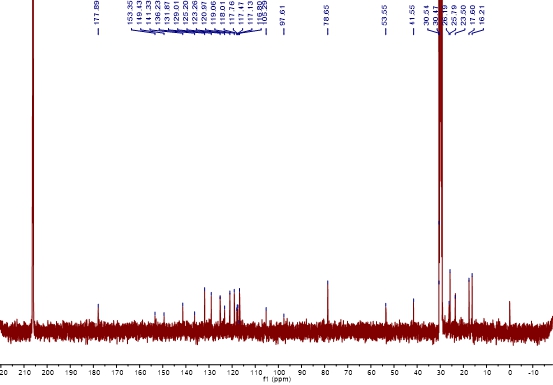


**Figure S18.** ^13^C NMR spectrum for **2** in acetone-*d*_6_.

**Figure S19.** ^1^H-^1^H gCOSY spectrum for **2** in acetone-*d*_6_


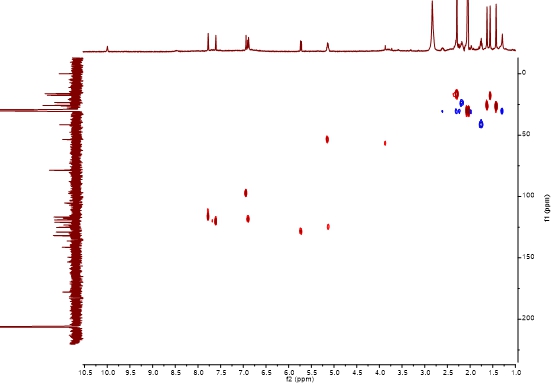


**Figure S20.** HSQC spectrum for **2** in acetone-*d*_6_.


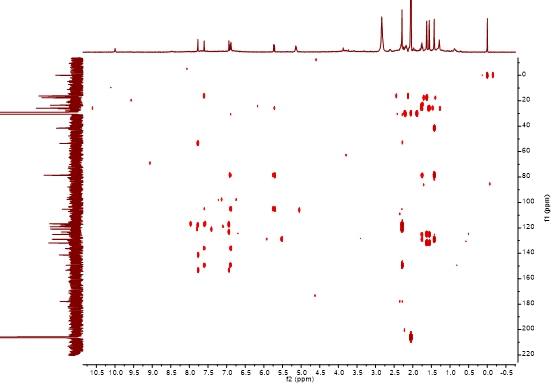


**Figure S21.** HMBC spectrum for **2** in acetone-*d*_6_.


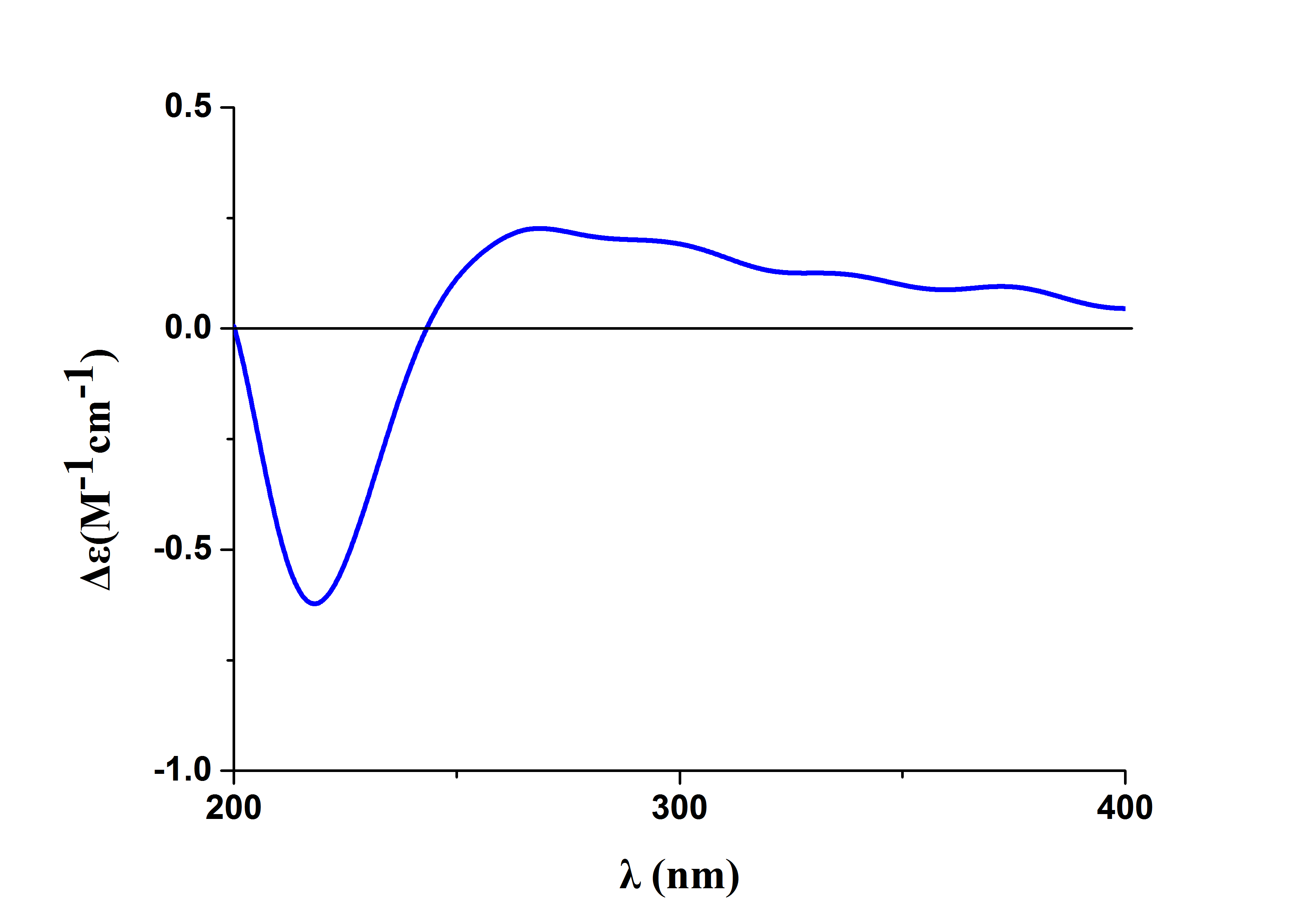


**Figure S22.** ECD spectrum for **2** in MeOH.


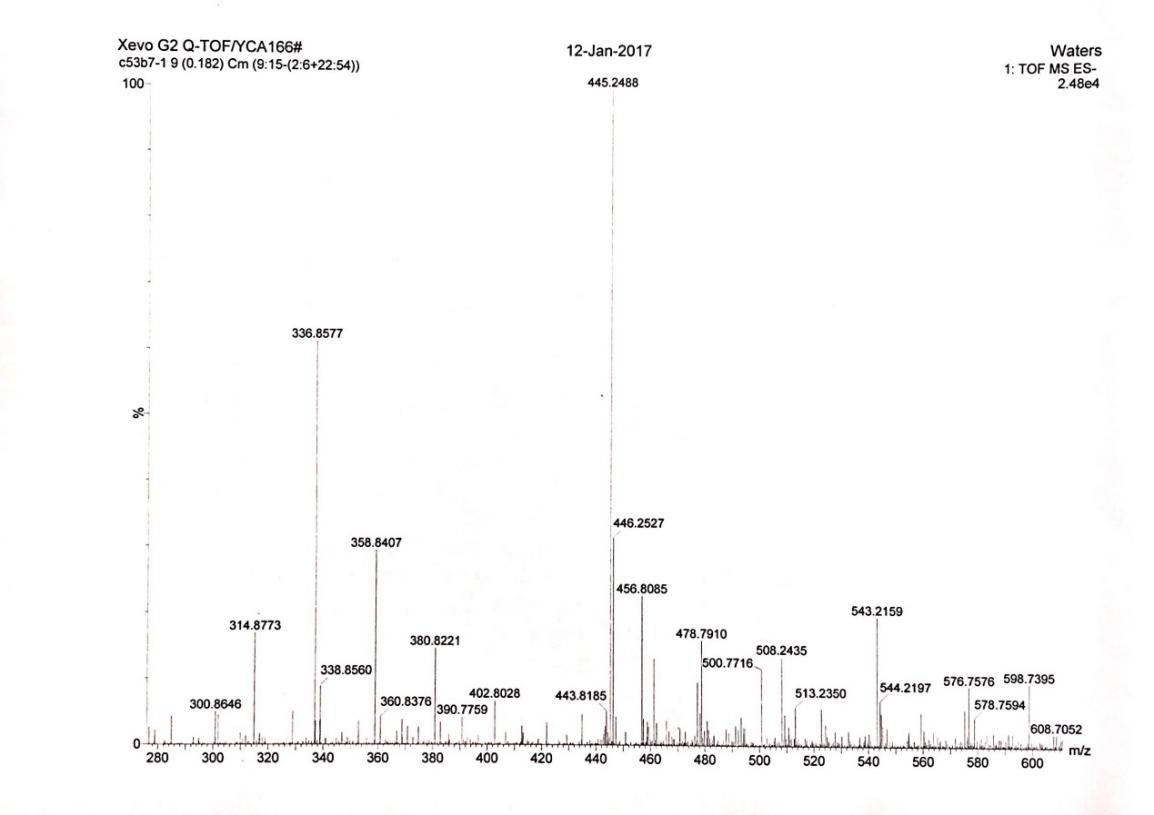


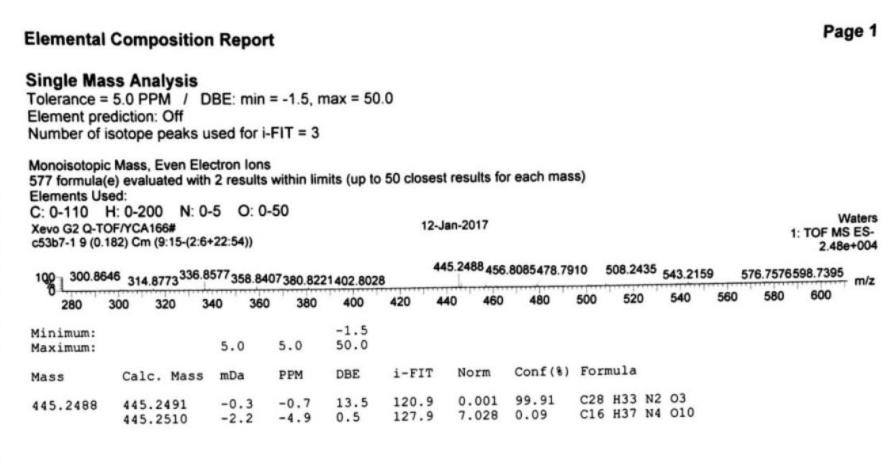


**Figure S23.** HRESIMS spectrum for **3**.


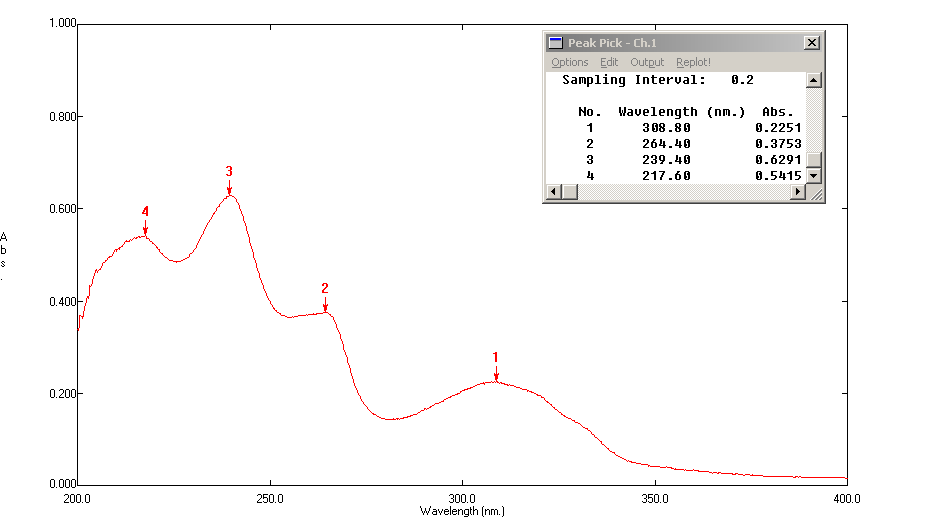


**Figure S24.** UV spectrum for **3**.


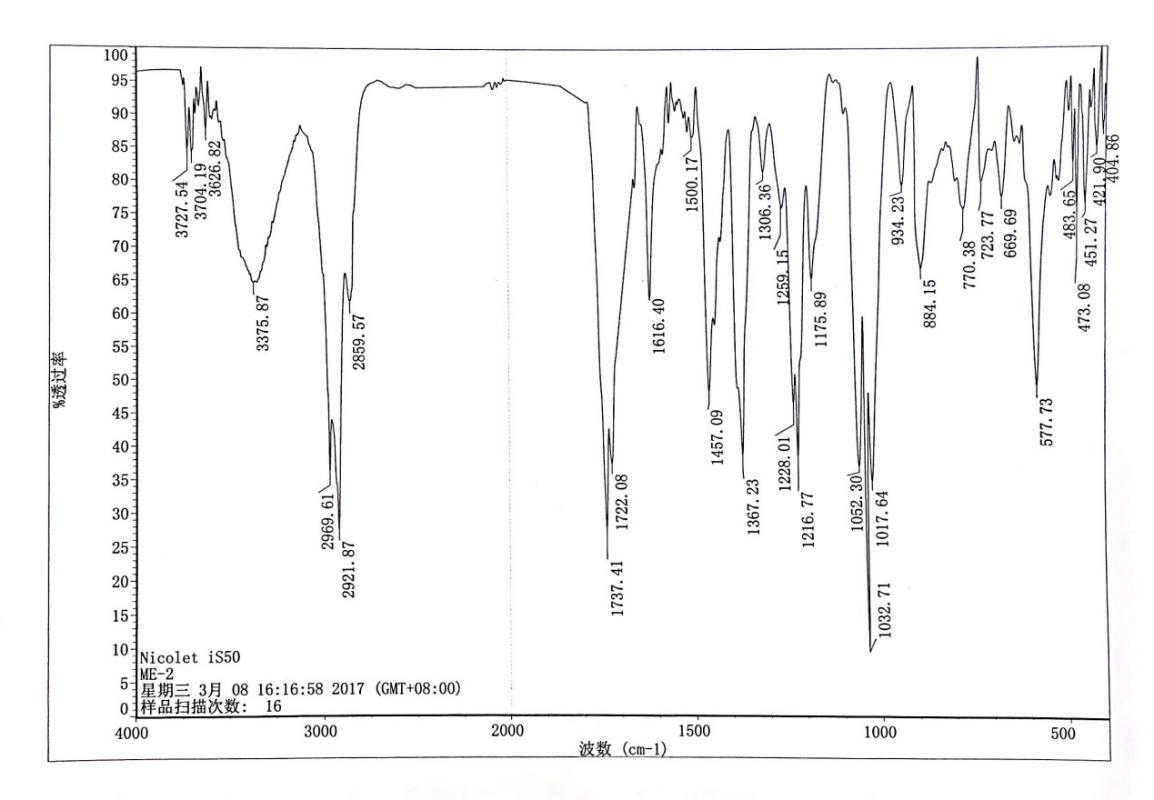


**Figure S25.** IR spectrum for **3**.


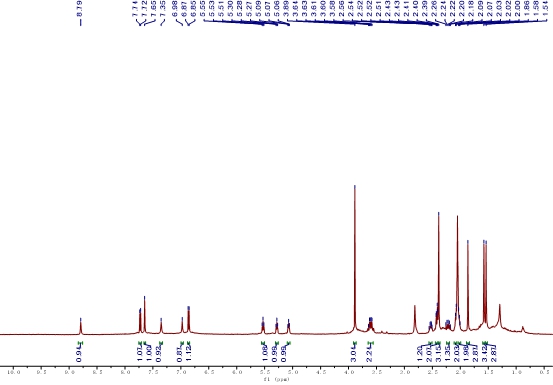


**Figure S26.** ^1^H NMR spectrum for **3** in acetone-*d*_6_.


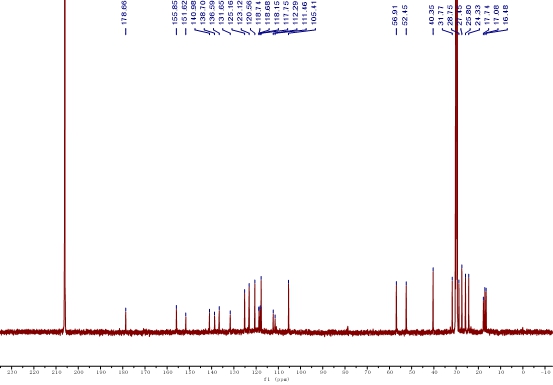


**Figure S27.** ^13^C NMR spectrum for **3** in acetone-*d*_6_.


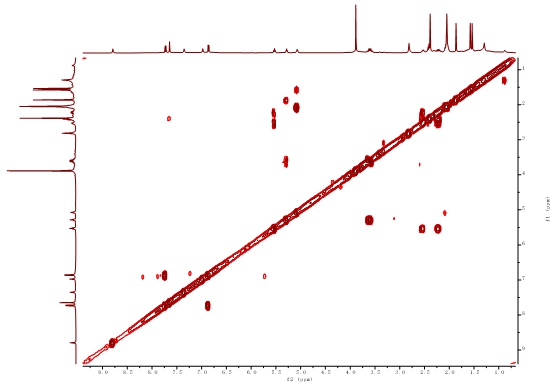


**Figure S28.** ^1^H-^1^H gCOSY spectrum for **3** in acetone-*d*_6_.


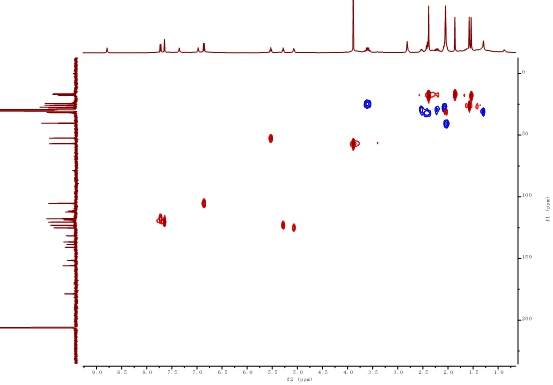


**Figure S29.** HSQC spectrum for **3** in acetone-*d*_6_.


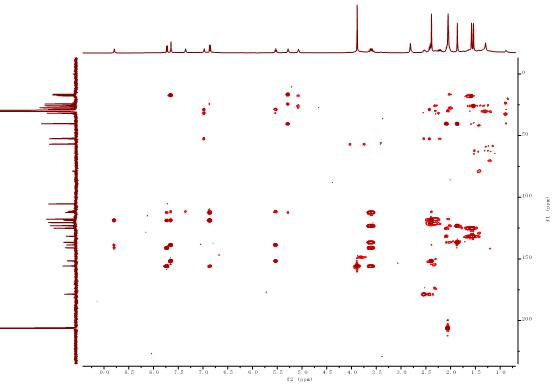


**Figure S30.** HMBC spectrum for **3** in acetone-*d*_6_.


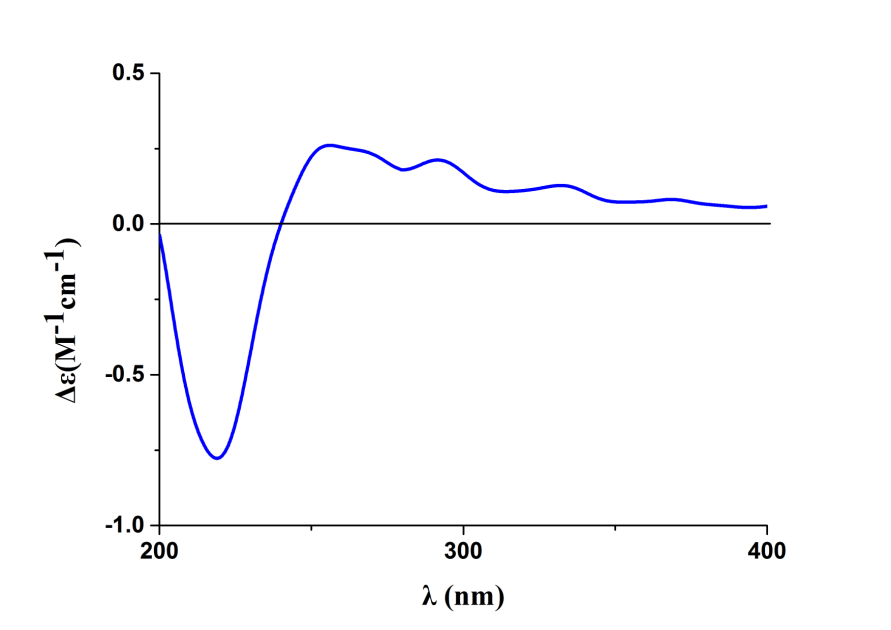


**Figure S31.** ECD spectrum for **3** in MeOH.


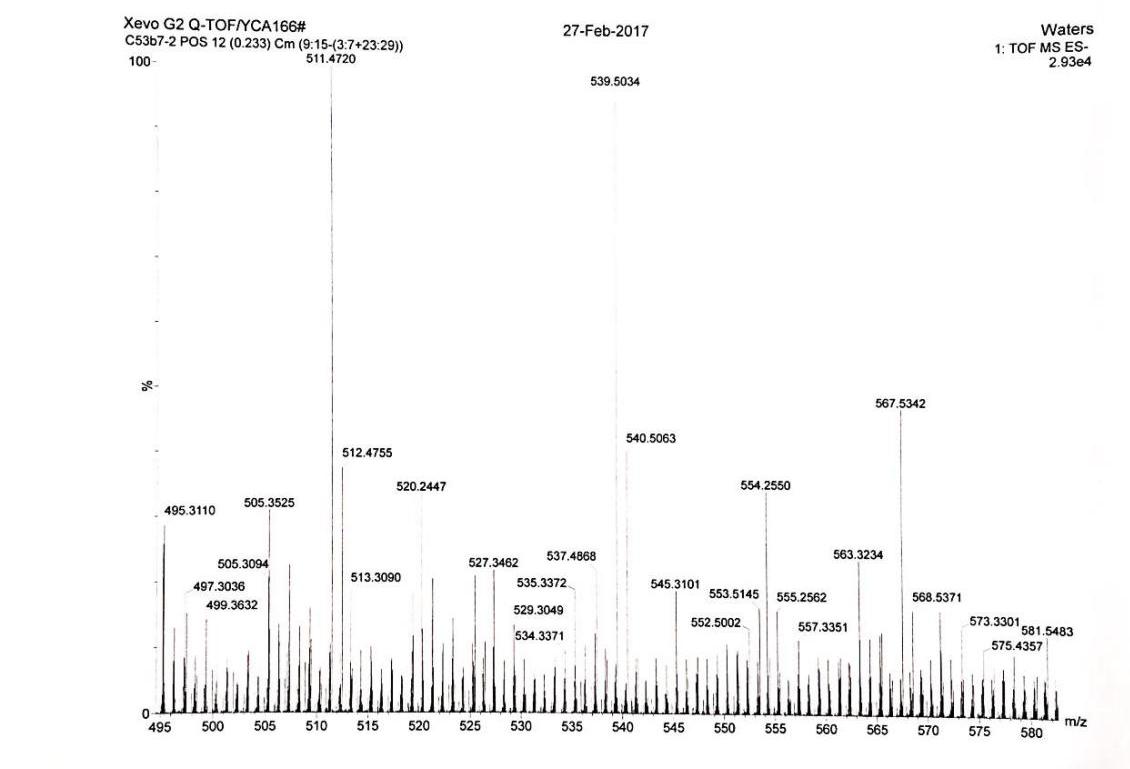


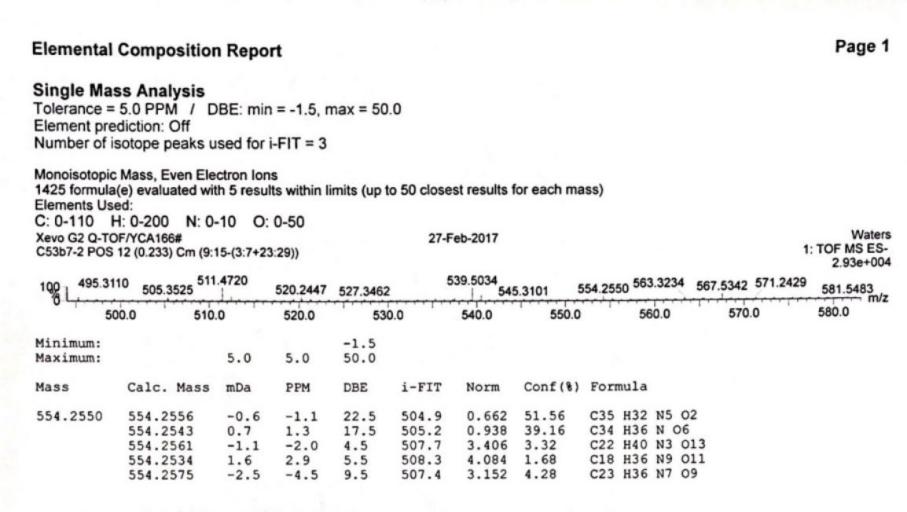


**Figure S32.** HRESIMS spectrum for **4**.


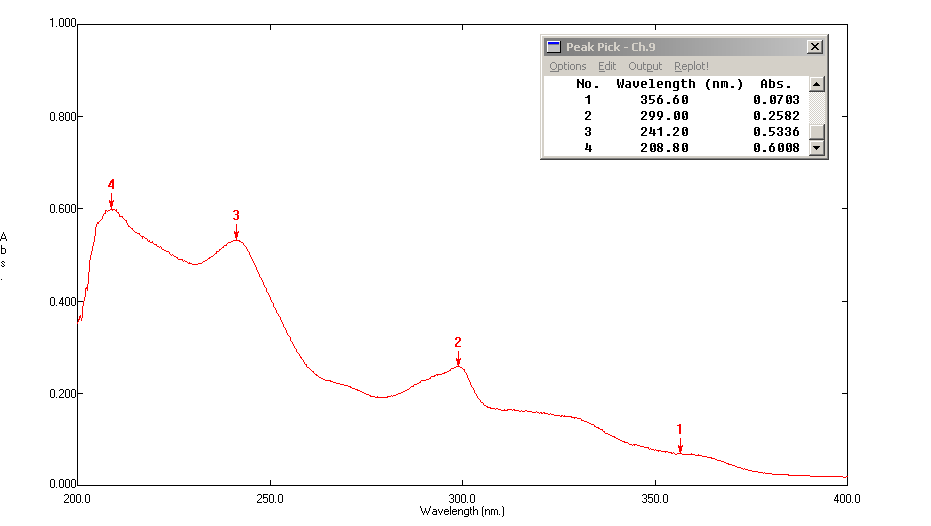


**Figure S33.** UV spectrum for **4**.


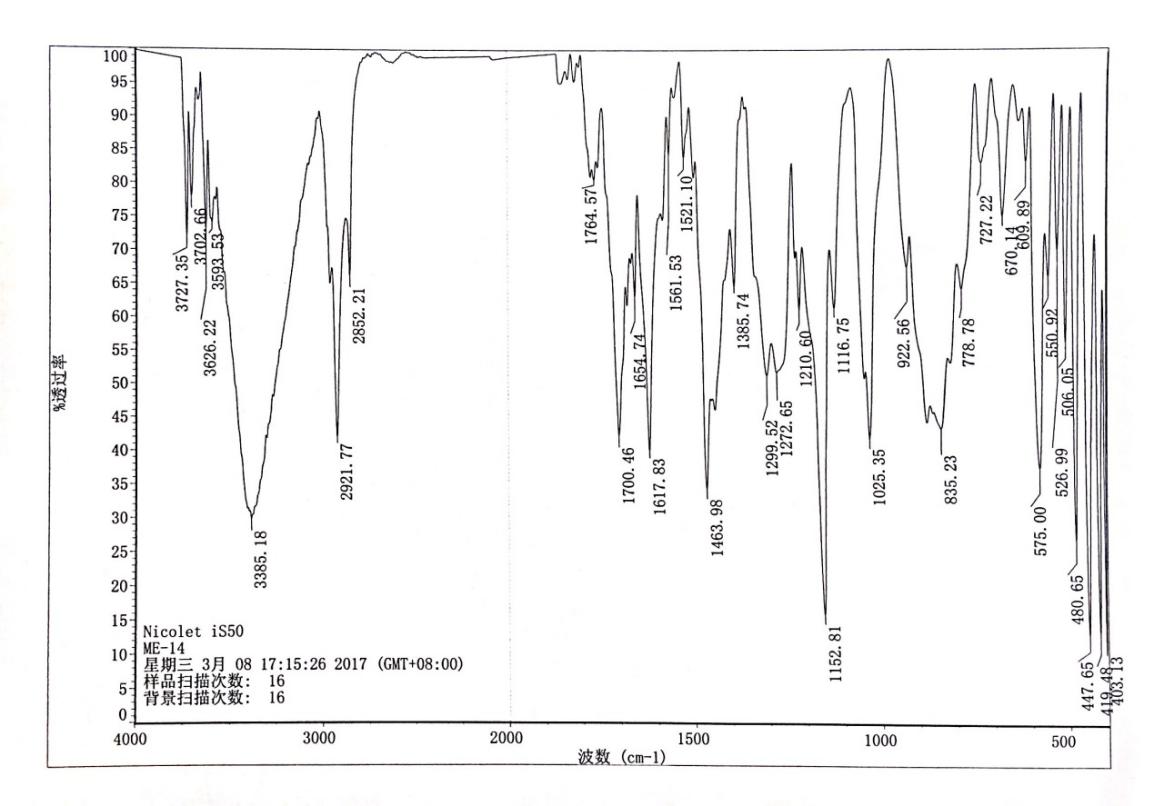


**Figure S34.** IR spectrum for **4**.


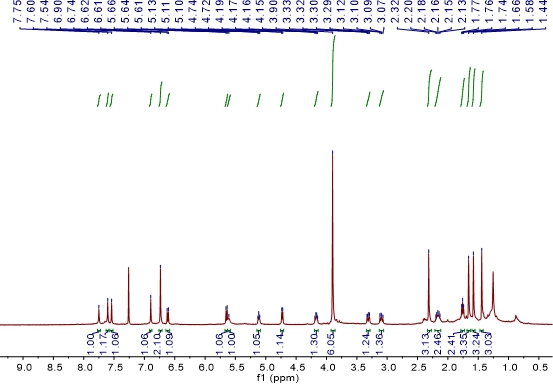


**Figure S35.** ^1^H NMR spectrum for **4** in CDCl_3_.


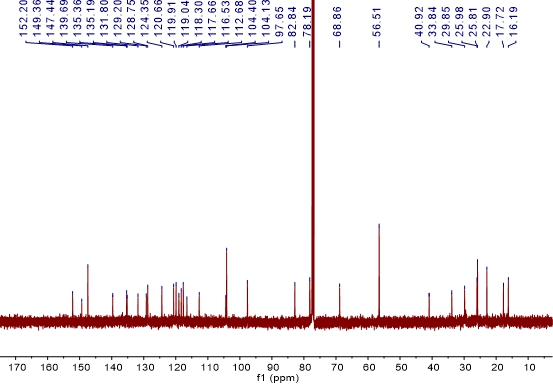


**Figure S36.** ^13^C NMR spectrum for **4** in CDCl_3_.


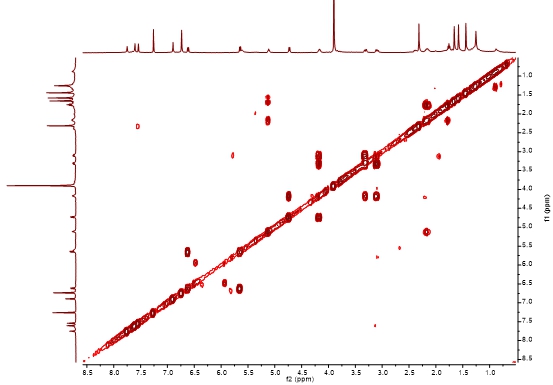


**Figure S37.** ^1^H-^1^H gCOSY spectrum for **4** in CDCl_3_.


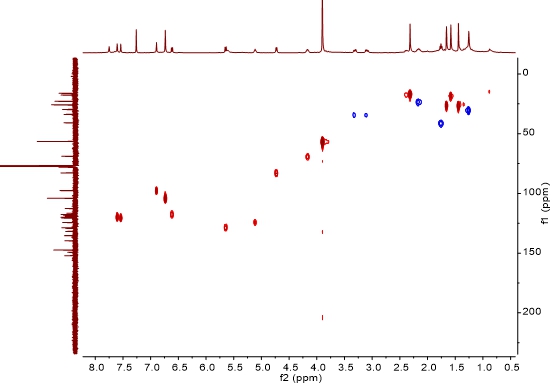


**Figure S38.** HSQC spectrum for **4** in CDCl_3_.


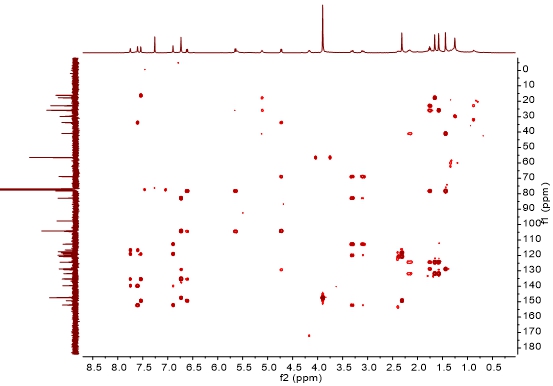


**Figure S39.** HMBC spectrum for **4** in CDCl_3_.


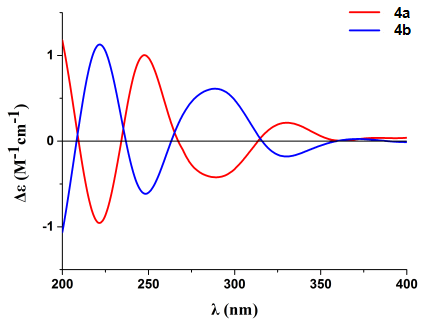


**Figure S40.** ECD spectra for **4a** and **4b** in MeOH.


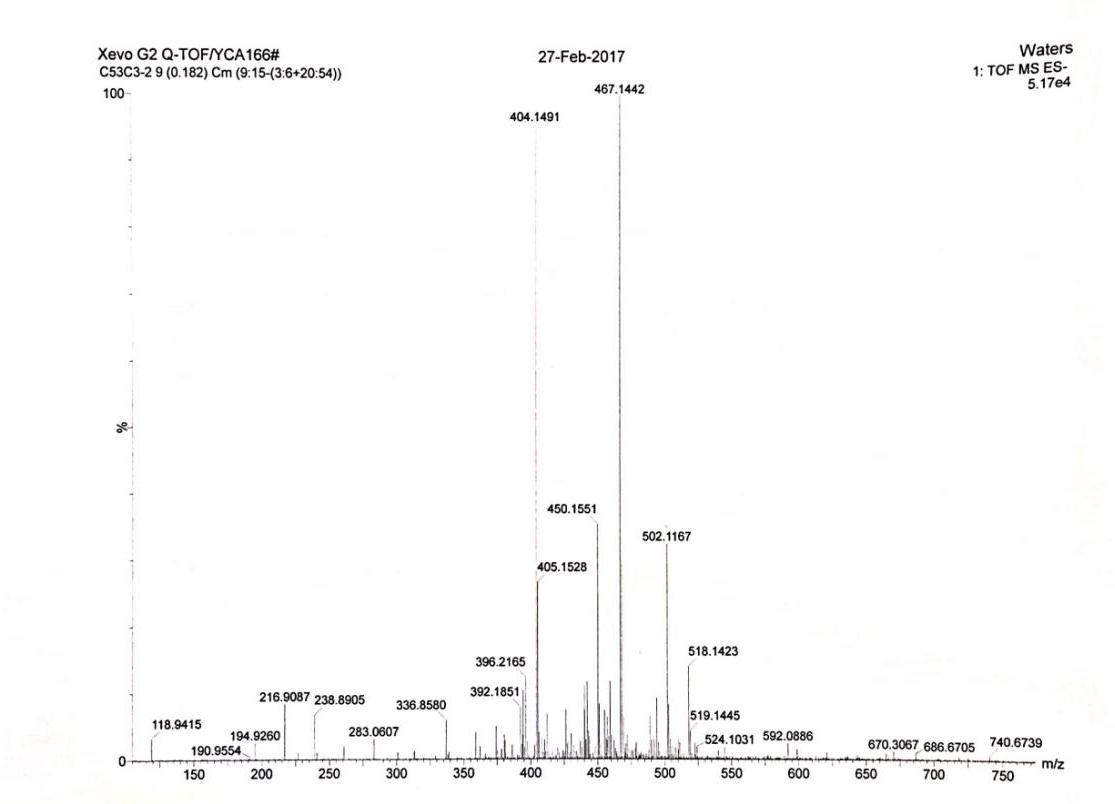


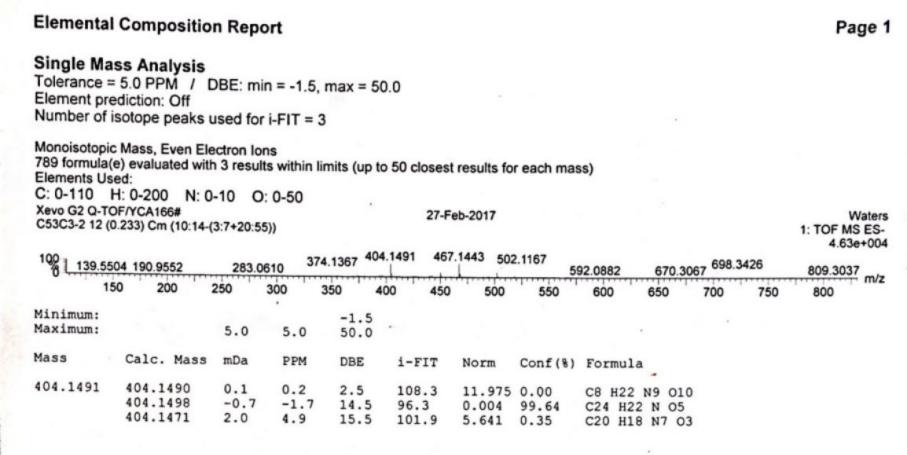


**Figure S41.** HRESIMS spectrum for **5**.


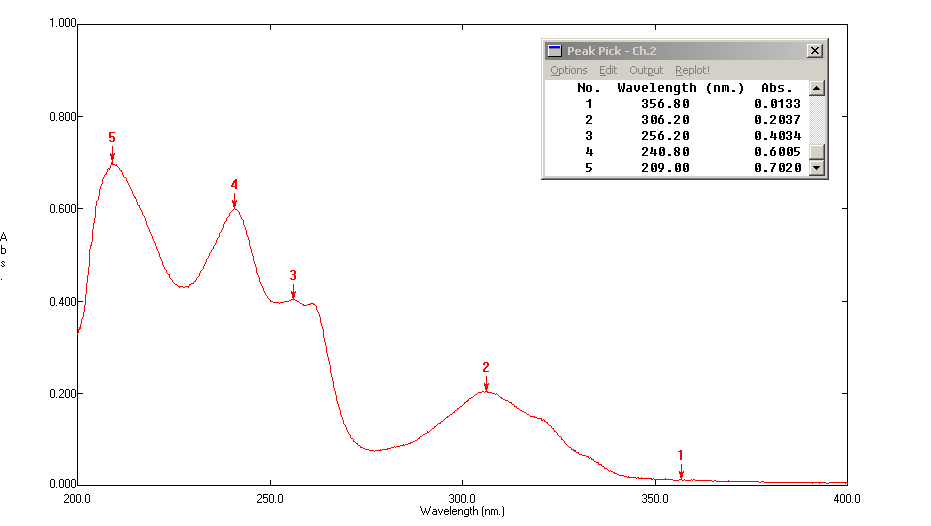


**Figure S42.** UV spectrum for **5**.


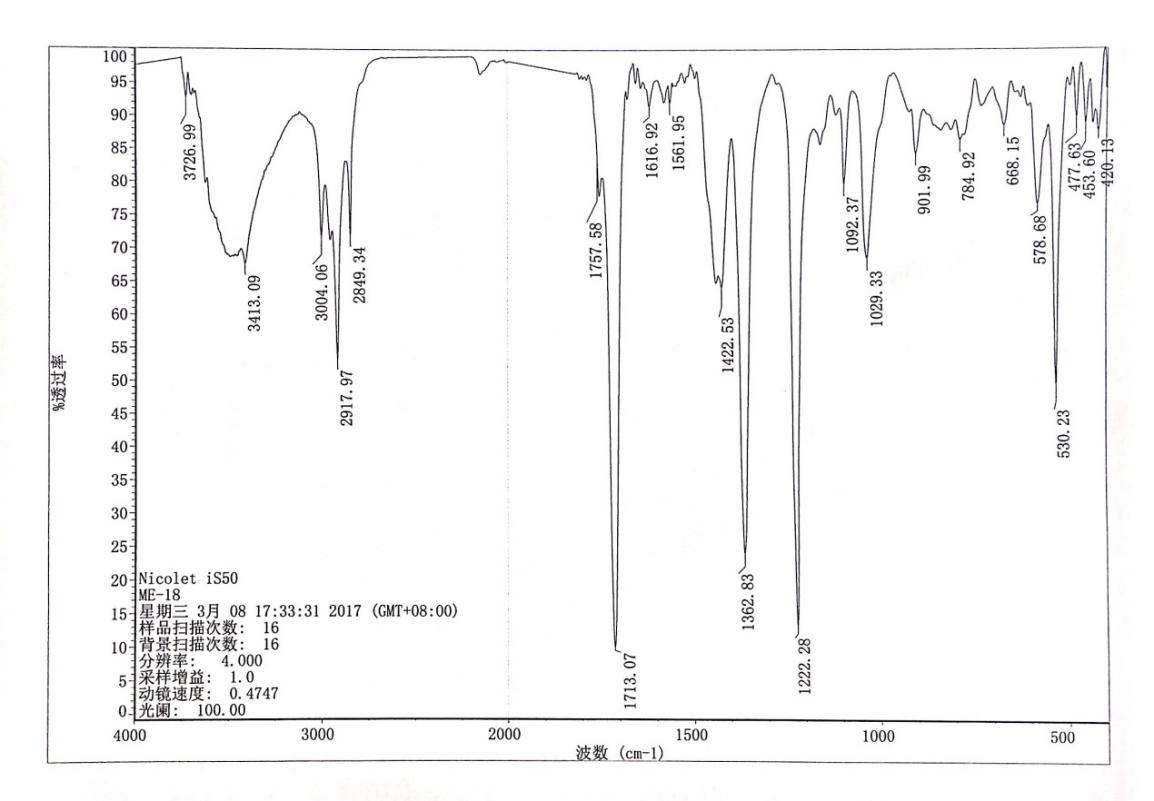


**Figure S43.** IR spectrum for **5**.


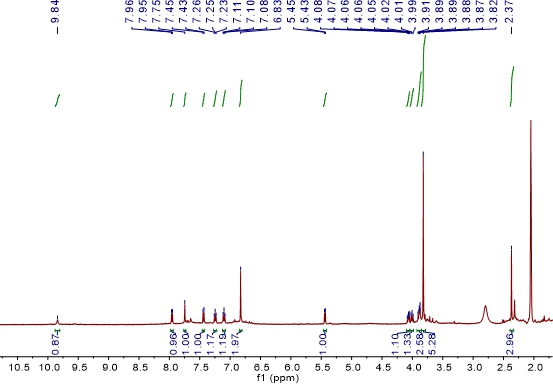


**Figure S44.** ^1^H NMR spectrum for **5** in acetone-*d*_6_.


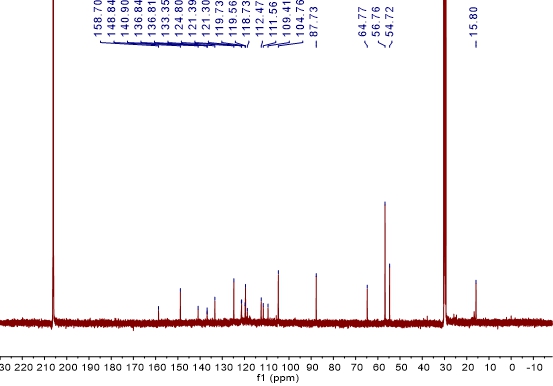


**Figure S45.** ^13^C NMR spectrum for **5** in acetone-*d*_6_.


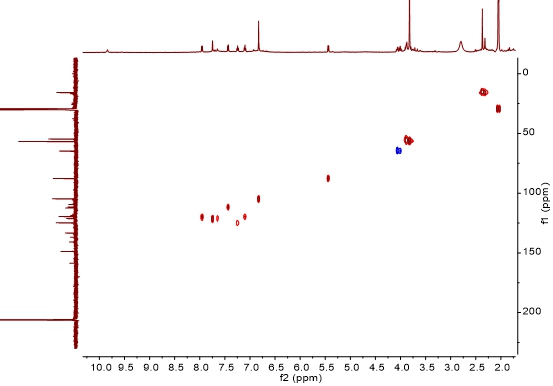


**Figure S46.** HSQC spectrum for **5** in acetone-*d*_6_.


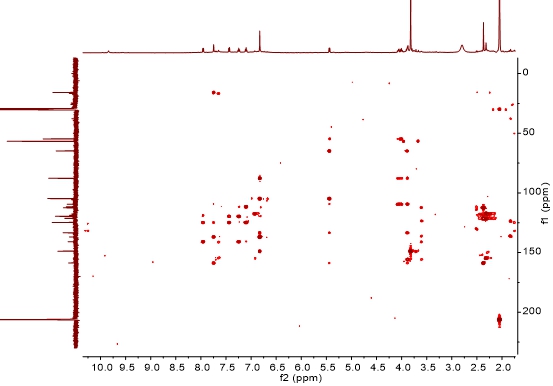


**Figure S47.** HMBC spectrum for **5** in acetone-*d*_6_.


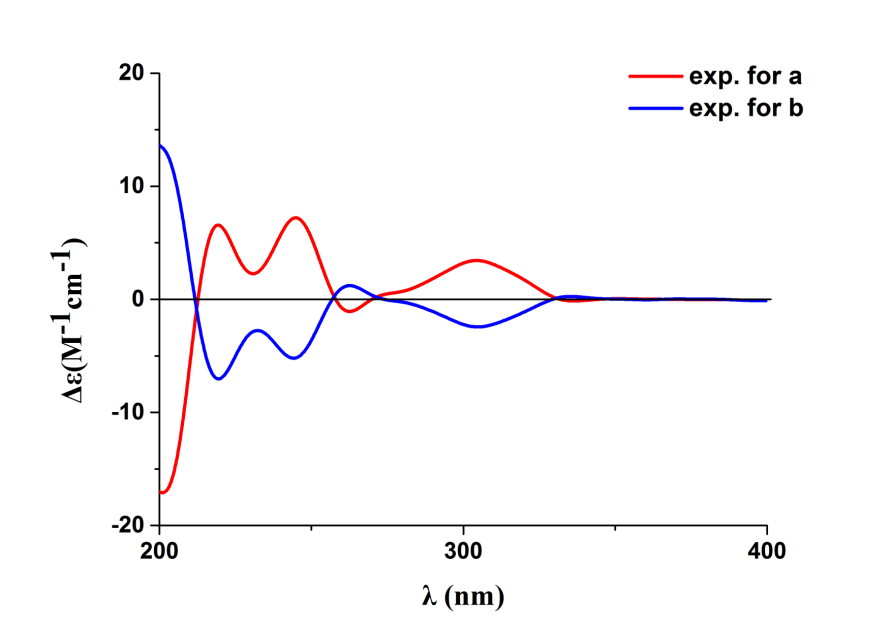


**Figure S48.** ECD spectra for **5a** and **5b** in MeOH.


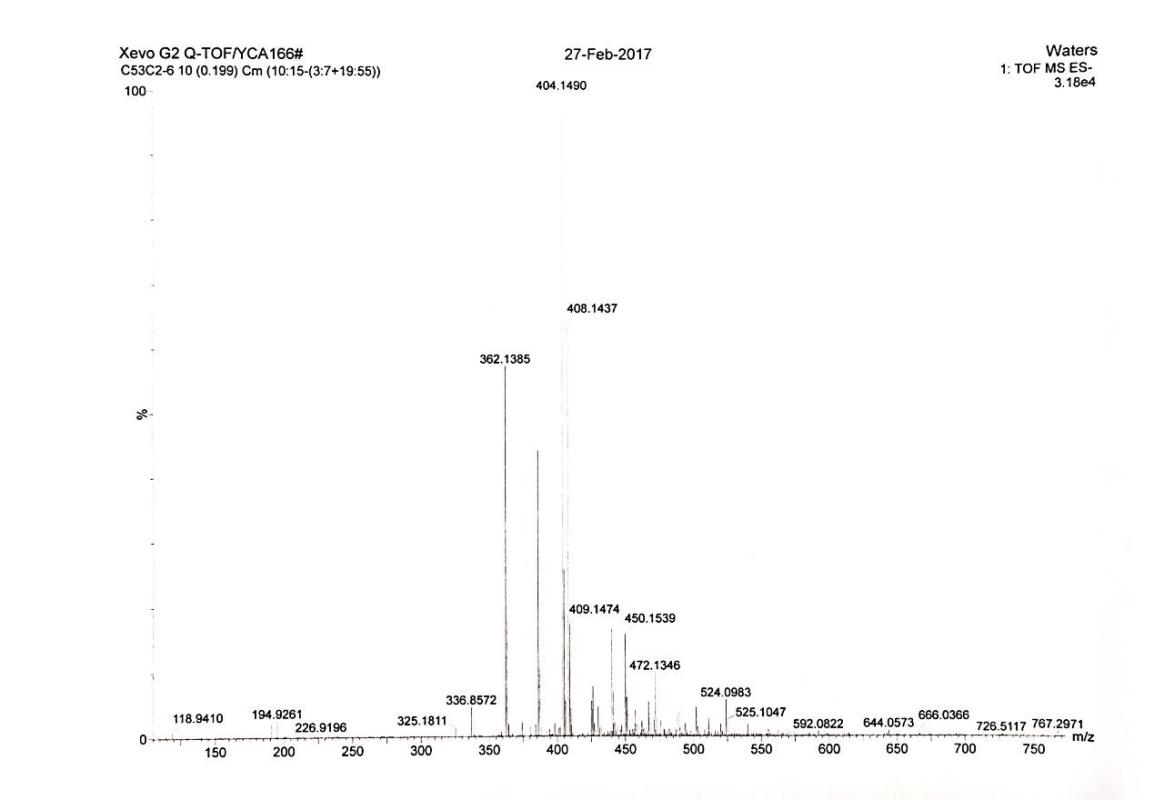


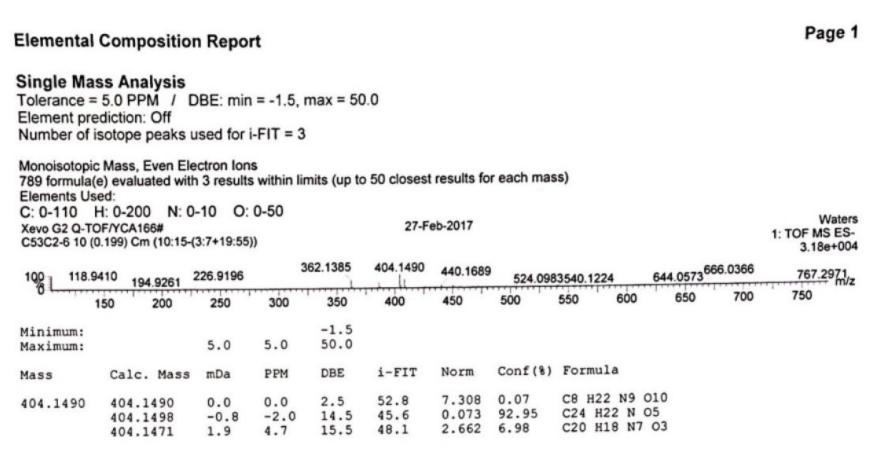


**Figure S49.** HRESIMS spectrum for **6**.


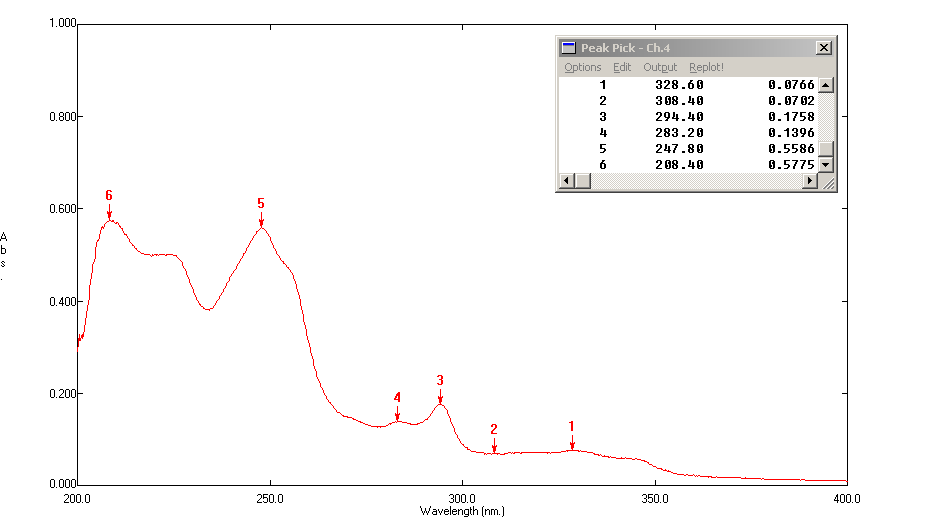


**Figure S50.** UV spectrum for **6**.


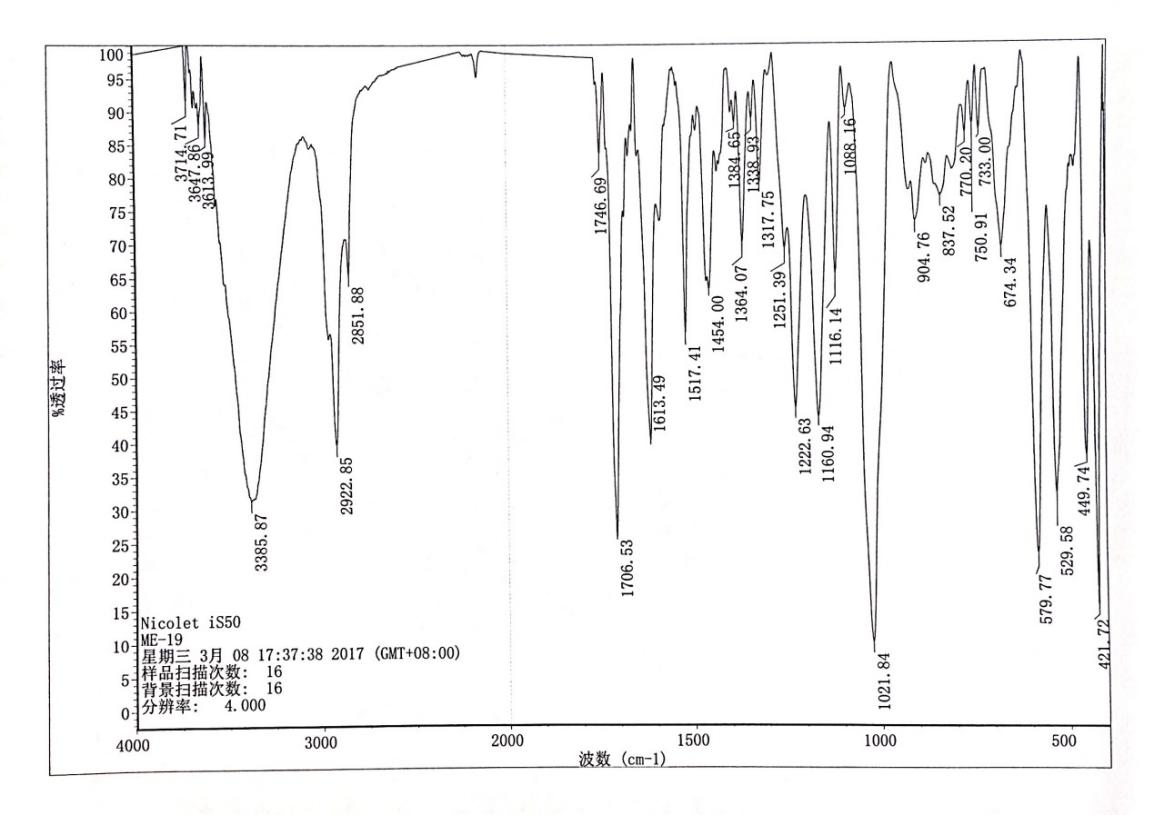


**Figure S51.** IR spectrum for **6**.


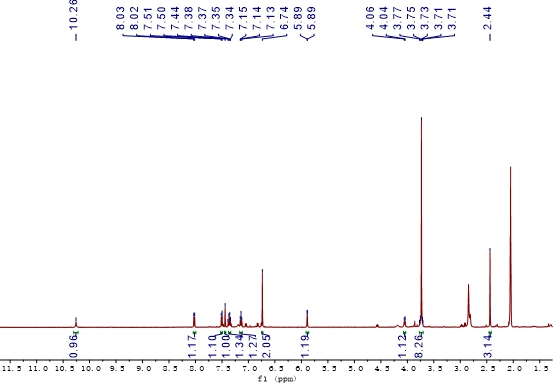


**Figure S52.** ^1^H NMR spectrum for **6** in acetone-*d*_6_.


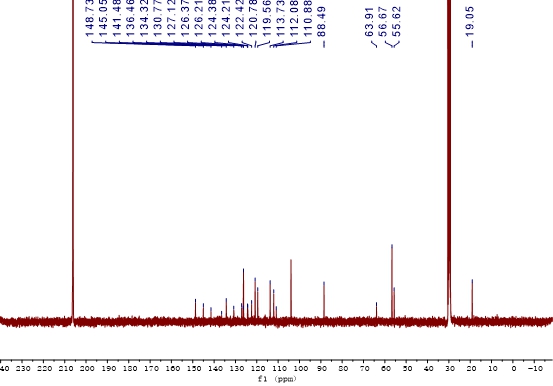


**Figure S53.** ^13^C NMR spectrum for **6** in acetone-*d*_6_.


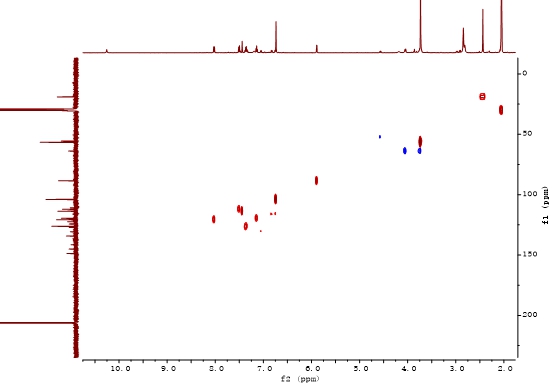


**Figure S54.** HSQC spectrum for **6** in acetone-*d*_6_.


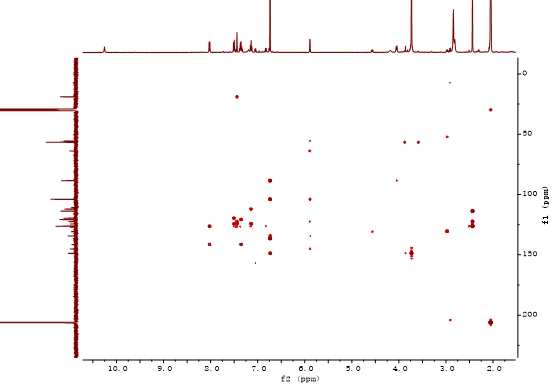


**Figure S55.** HMBC spectrum for **6** in acetone-*d*_6_.


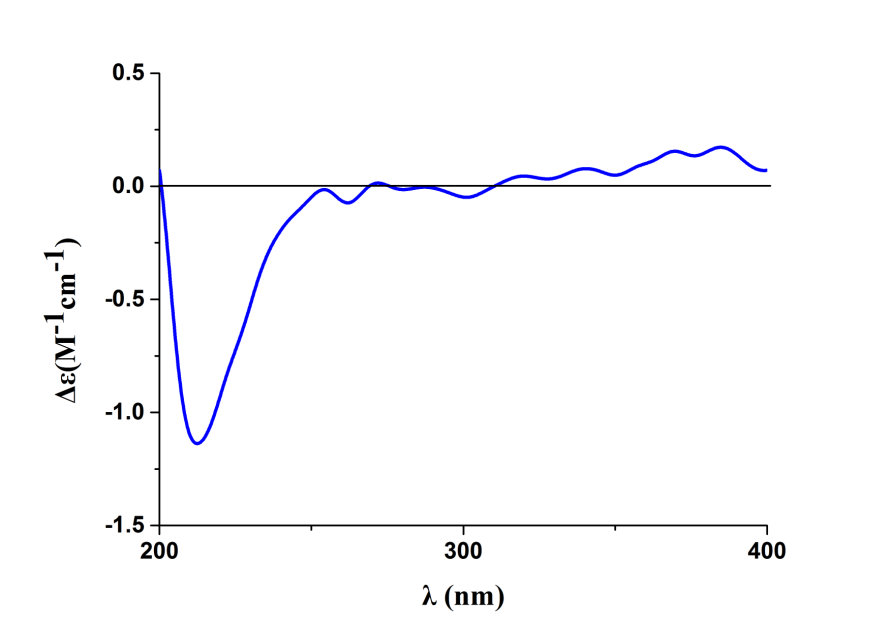


**Figure S56.** ECD spectrum for **6** in MeOH.


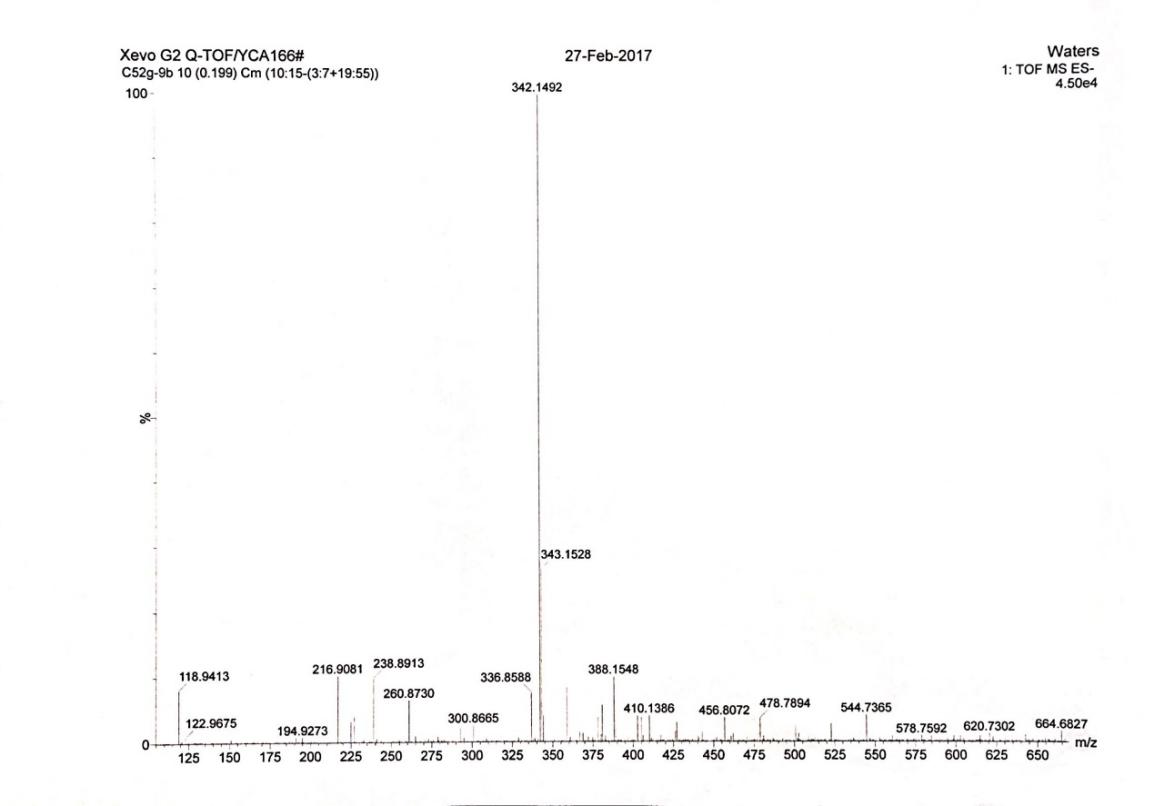


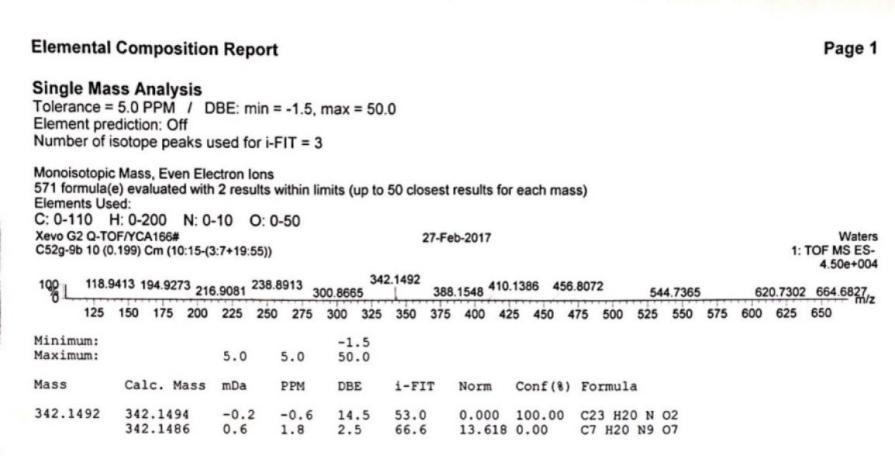


**Figure S57.** HRESIMS spectrum for **7**.


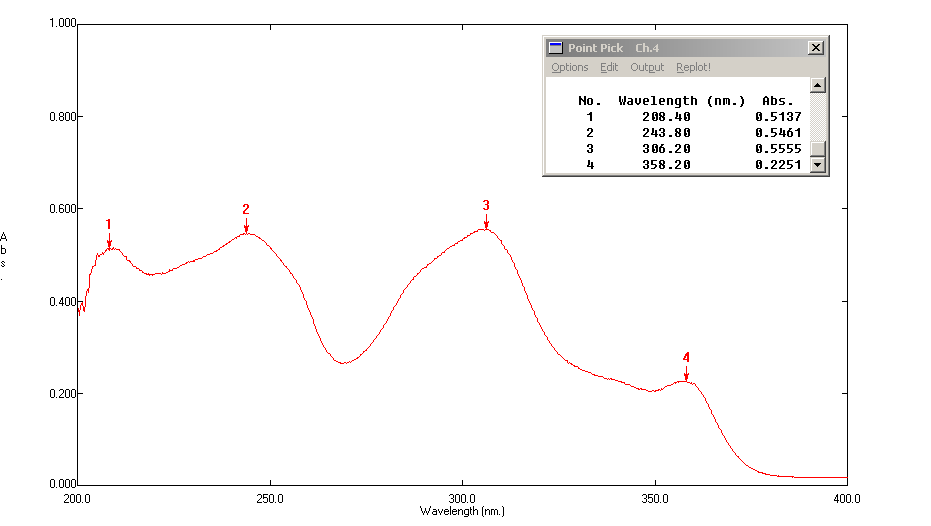


**Figure S58.** UV spectrum for **7**.


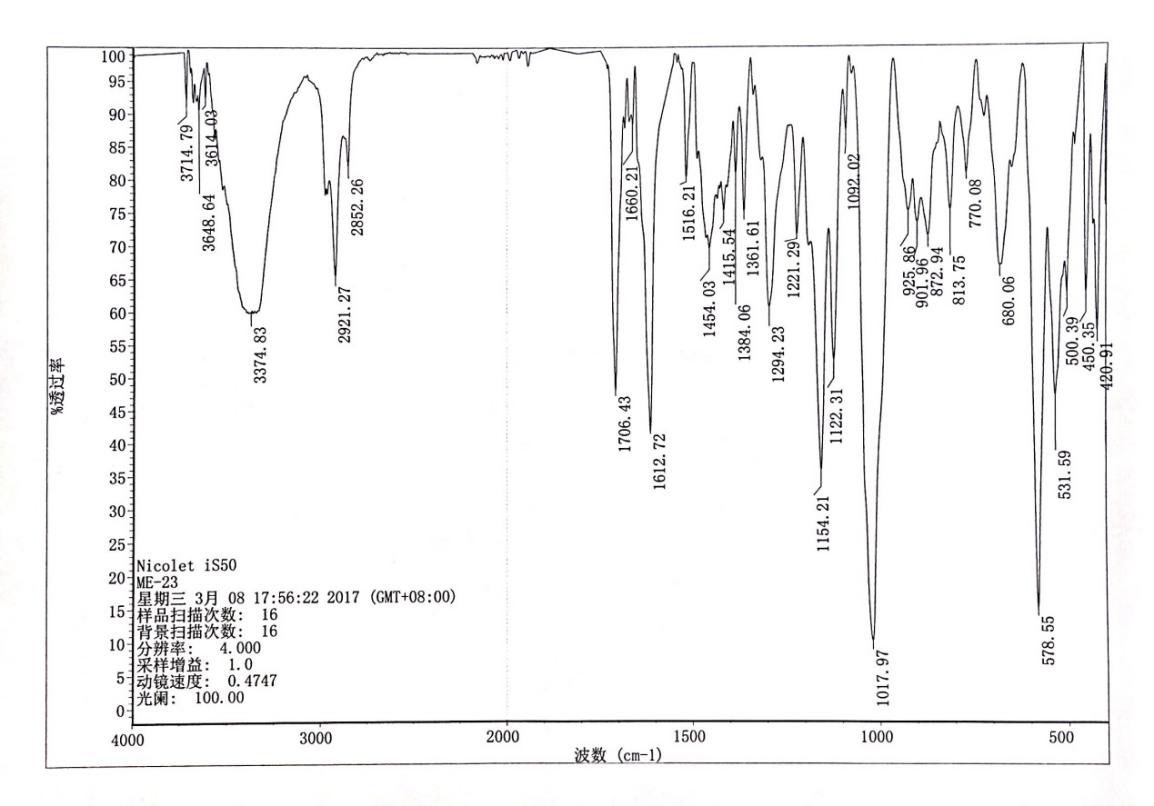


**Figure S59.** IR spectrum for **7**.


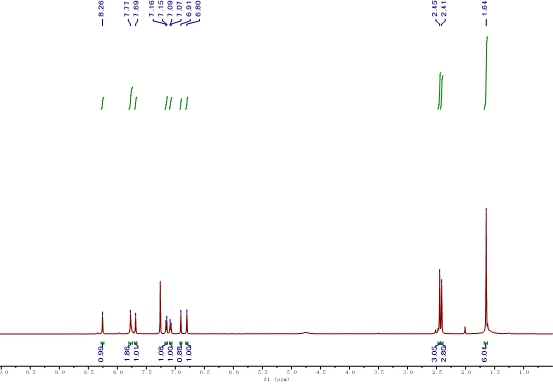


**Figure S60.** ^1^H NMR spectrum for **7** in CDCl_3_.


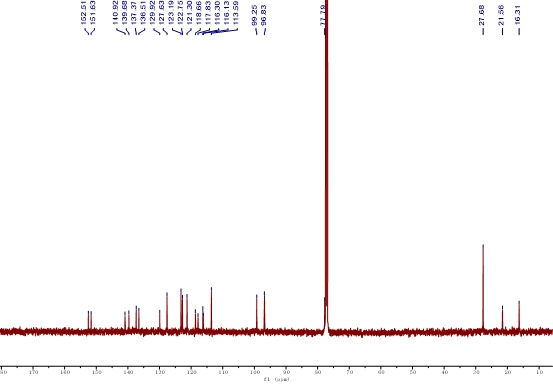


**Figure S61.** ^13^C NMR spectrum for **7** in CDCl_3_.

**Figure S62.** ^1^H-^1^H gCOSY spectrum for **7** in CDCl_3_.


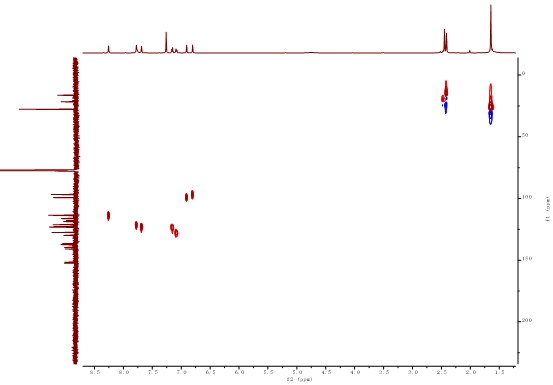


**Figure S63.** HSQC spectrum for **7** in CDCl_3_.


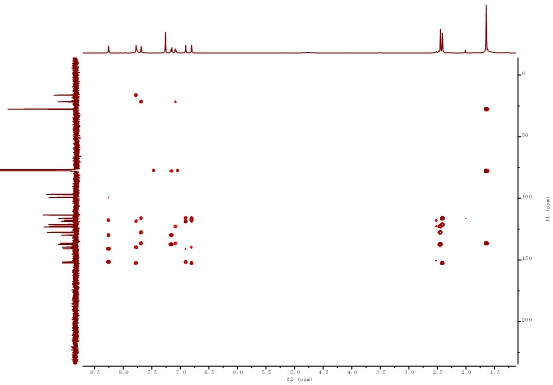


**Figure S64.** HMBC spectrum for **7** in CDCl_3_.

**Elemental Composition Report**

**Single Mass Analysis**

Tolerance = 5.0 PPM / DBE: min = -1.5, max = 50.0

Element prediction: Off

Number of isotope peaks used for i-FIT = 3

Monoisotopic Mass, Even Electron Ions

422 formula(e) evaluated with 2 results within limits (up to 50 closest results for each mass)

Elements Used:

C: 0-110 H: 0-200 N: 0-5 O: 0-50

| Minimum: |  |  |  | -1.5 |  |  |  |  |
| --- | --- | --- | --- | --- | --- | --- | --- | --- |
| Maximum: |  | 5 | 5 | 50 |  |  |  |  |
| Mass | Calc. Mass | mDa | PPM | DBE | i-FIT | Norm | Conf(%) | Formula |
| 378.2072 | 378.2069 | 0.3 | 0.8 | 11.5 | 269.9 | 0 | 100 | C24 H28 N O3 |
|  | 378.2088 | -1.6 | -4.2 | -1.5 | 285.2 | 15.336 | 0 | C12 H32 N3 O10 |

**Figure S65.** HRESIMS spectrum for **8**.


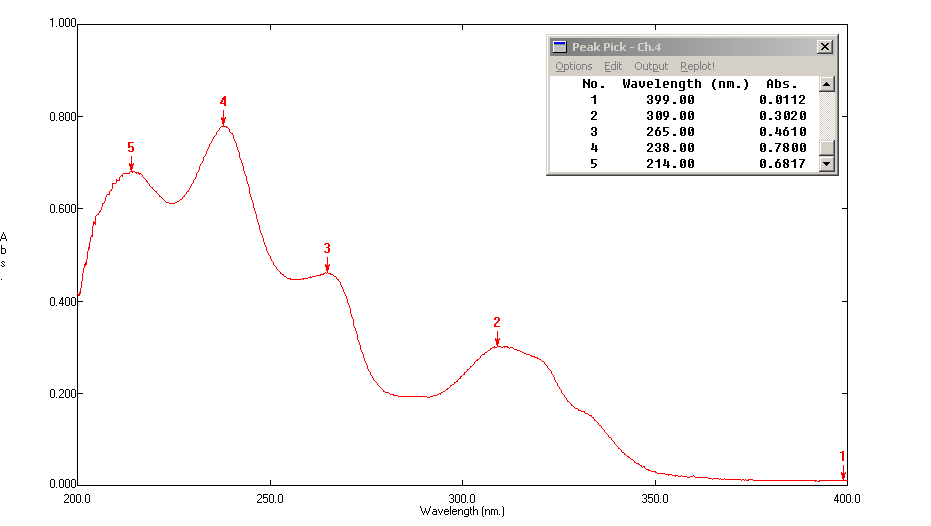


**Figure S66.** UV spectrum for **8**.


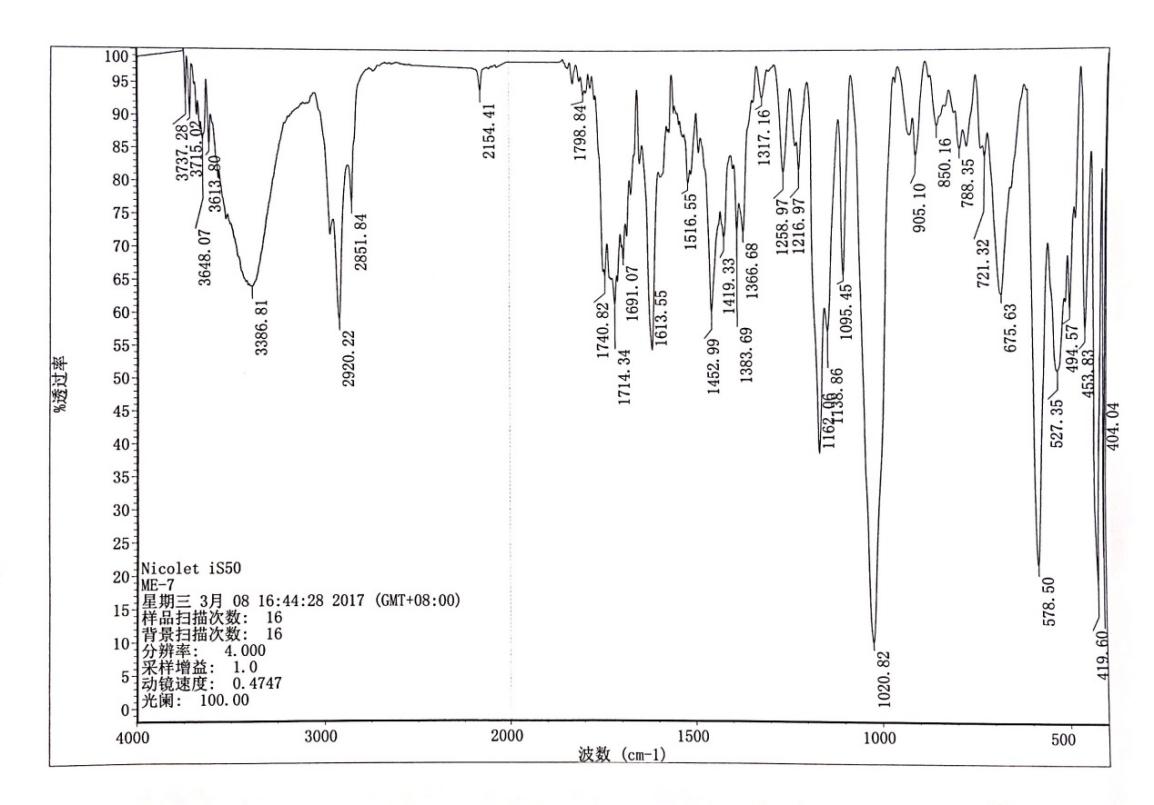


**Figure S67.** IR spectrum for **8**.

**Figure S68.** ^1^H NMR spectrum for **8** in CDCl_3_.

**Figure S69.** ^13^C NMR spectrum for **8** in CDCl_3_.

**Figure S70.** HSQC spectrum for **8** in CDCl_3_.

**Figure S71.** HMBC spectrum for **8** in CDCl_3_.

**Figure S72.** ECD spectrum for **8** in MeOH.

**Figure S73.** HRESIMS spectrum for **9**.

**Figure S74.** UV spectrum for **9**.

**Figure S75.** IR spectrum for **9**.

**Figure S76.** ^1^H NMR spectrum for **9** in acetone-*d*_6_.

**Figure S77.** ^13^C NMR spectrum for **9** in acetone-*d*_6_.

**Figure S78.** HSQC spectrum for **9** in acetone-*d*_6_.

**Figure S79.** HMBC spectrum for **9** in acetone-*d*_6_.

**Figure S80.** ECD spectrum for **9** in MeOH.

**Figure S81.** HRESIMS spectrum for **10**.

**Figure S82.** UV spectrum for **10**.

**Figure S83.** IR spectrum for **10**.

**Figure S84.** ^1^H NMR spectrum for 1**0** in CDCl_3_.

**Figure S85.** ^13^C NMR spectrum for **10** in CDCl_3_.

**Figure S86.** HSQC spectrum for **10** in CDCl_3_.

**Figure S87** HMBC spectrum for **10** in CDCl_3_.

**Figure S88.** HRESIMS spectrum for **11**.

**Figure S89.** UV spectrum for **11***.

**Figure S90.** IR spectrum for **11**.

**Figure S91.** ^1^H NMR spectrum for **11** in CDCl_3_.

**Figure S92.** ^13^C NMR spectrum for **11** in CDCl_3_.

**Figure S93.** HSQC spectrum for **11** in CDCl_3_.

**Figure S94.** HMBC spectrum for **11** in CDCl_3_.

**Figure S95.** ECD spectrum for **11** in MeOH.

**Figure S96.** HRESIMS spectrum for **12**.

**Figure S97.** UV spectrum for **12**.

**Figure S98.** IR spectrum for **12**.

**Figure S99.** ^1^H NMR spectrum for **12** in CDCl_3_.

**Figure S100.** ^13^C NMR spectrum for **12** in CDCl_3_.

**Figure S101.** HSQC spectrum for **12** in CDCl_3_.

**Figure S102.** HMBC spectrum for **12** in CDCl_3_.

**Figure S103.** HRESIMS spectrum for **13**.

**Figure S104.** UV spectrum for **13**.

**Figure S105.** IR spectrum for **13**.

**Figure S106.** ^1^H NMR spectrum for **13** in acetone-*d*_6_.

**Figure S107.** ^13^C NMR spectrum for **13** in acetone-*d*_6_.

**Figure S108.** HSQC spectrum for **13** in acetone-*d*_6_.

**Figure S109.** HMBC spectrum for **13** in acetone-*d*_6_.

**Figure S110.** HRESIMS spectrum for **14**.

**Figure S111.** UV spectrum for **14**.

**Figure S112.** IR spectrum for **14**.

**Figure S113.** ^1^H NMR spectrum for **14** in acetone-*d*_6_.

**Figure S114.** ^13^C NMR spectrum for **14** in acetone-*d*_6_.

**Figure S115.** HSQC spectrum for **14** in acetone-*d*_6_.

**Figure S116.** HMBC spectrum for **14** in acetone-*d*_6_.

**Figure S117.** HRESIMS spectrum for **15**.

**Figure S118.** UV spectrum for **15**.

**Figure S119.** IR spectrum for **15**.

**Figure S120.** ^1^H NMR spectrum for **15** in acetone-*d*_6_.

**Figure S121.** ^13^C NMR spectrum for **15** in acetone-*d*_6_.

**Figure S122.** HSQC spectrum for **15** in acetone-*d*_6_

**Figure S123.** HMBC spectrum for **15** in acetone-*d*_6_.
